# Simple Synthetic Routes to Carbene‐M‐Amido (M=Cu, Ag, Au) Complexes for Luminescence and Photocatalysis Applications

**DOI:** 10.1002/chem.202101476

**Published:** 2021-06-28

**Authors:** Nikolaos V. Tzouras, Ekaterina A. Martynova, Xinyuan Ma, Thomas Scattolin, Benjamin Hupp, Hendrik Busen, Marina Saab, Ziyun Zhang, Laura Falivene, Gianmarco Pisanò, Kristof Van Hecke, Luigi Cavallo, Catherine S. J. Cazin, Andreas Steffen, Steven P. Nolan

**Affiliations:** ^1^ Department of Chemistry and Centre for Sustainable Chemistry Ghent University Krijgslaan 281,S-3 9000 Ghent Belgium; ^2^ Faculty of Chemistry and Chemical Biology TU Dortmund University Otto-Hahn-Str. 6 44227 Dortmund Germany; ^3^ KAUST Catalysis Center Physical Sciences and Engineering Division King Abdullah University of Science and Technology Thuwal 23955-6900 Saudi Arabia

**Keywords:** carbene-metal-amides, coinage metal-N-heterocyclic carbene complexes, mechanochemistry, photocatalysis, synthetic methods

## Abstract

The development of novel and operationally simple synthetic routes to carbene‐metal‐amido (CMA) complexes of copper, silver and gold relevant for photonic applications are reported. A mild base and sustainable solvents allow all reactions to be conducted in air and at room temperature, leading to high yields of the targeted compounds even on multigram scales. The effect of various mild bases on the N−H metallation was studied in silico and experimentally, while a mechanochemical, solvent‐free synthetic approach was also developed. Our photophysical studies on [M(NHC)(Cbz)] (Cbz=carbazolyl) indicate that the occurrence of fluorescent or phosphorescent states is determined primarily by the metal, providing control over the excited state properties. Consequently, we demonstrate the potential of the new CMAs beyond luminescence applications by employing a selected CMA as a photocatalyst. The exemplified synthetic ease is expected to accelerate the applications of CMAs in photocatalysis and materials chemistry.

## Graphical abstract

**N−H metallation routes**: Interest in carbene‐metal‐amido (CMA) complexes of the coinage metals has inspired comprehensive efforts towards simplifying their synthesis. We now showcase simple and sustainable routes to various CMAs, using mild conditions for the N−H metallation of carbazole and other amines. The action of a mild base is studied computationally and the photophysical properties of some new CMA complexes are examined.

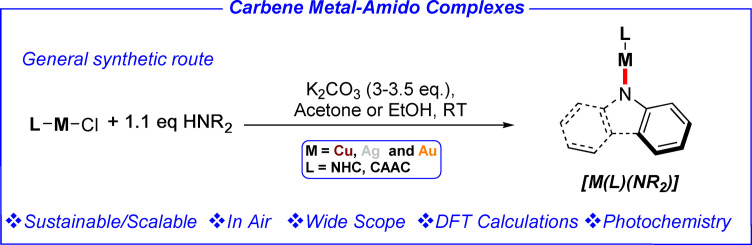

## Introduction

In the course of the last five years, carbene‐metal‐amido (CMA) complexes of the coinage metals have emerged as highly promising candidates for application in organic light‐emitting diodes (OLEDS) and other next‐generation photonic applications.[^1^, ^2^, ^3^, ^4^, ^5^, ^6^, ^7^, ^8^, ^9^, ^10^, ^11^, ^12^, ^13^, ^14^] Some examples have already been demonstrated in highly luminescent OLEDS.[^4^, ^6^, ^7^, ^8^, ^9^, ^11^, ^12^, ^13^] The excited state properties of these donor‐bridge‐acceptor organometallic emitters have been investigated thoroughly and the structural features which modulate those properties have been largely established.[^15^, ^16^, ^17^, ^18^] The key structural components needed are: i) a simple amide donor such as the carbazole framework, ii) a linear, two‐coordinate d^10^ coinage metal (Cu, Ag, Au), and iii) an acceptor ligand of the carbene type.^[17]^ For the latter, the most frequently encountered ones are those of the cyclic (alkyl)(amino) carbene and monoamido‐ or diamido‐carbene (MAC and DAC, respectively) families,[^3^, ^4^, ^5^, ^6^, ^7^, ^8^, ^9^, ^10^, ^11^, ^12^, ^13^, ^14^] while examples bearing the more common (benz)imidazolylidene framework and a mesoionic carbene have been reported only very recently.[^19^, ^20^] The peculiar combination of electronic properties of the single components enables the population of ligand‐to‐ligand (LL)CT states with a small energy gap between the singlet and triplet excited states, which is highly beneficial for emission via Thermally Activated Delayed Fluoresence (TADF) with extraordinarily high radiative rate constants *k*_r_.[^3^, ^4^, ^5^, ^6^, ^7^, ^8^, ^9^, ^10^, ^11^, ^12^, ^13^, ^14^, ^17^, ^18^, ^19^, ^21^] However, variations of the general formula, specifically to make it more suitable for other photophysical, ‐chemical or ‐catalytic applications, has been of comparatively lower priority. A rare exception is a recent study by Li et al., where the Cu‐carbazolyl moiety was combined with common imidazolylidenes as weak π‐acceptors.^[19a]^ The authors postulate that the balance of LLCT and ligand centered (LC) ππ* states localized at the carbazolyl was tipped towards the latter, resulting in dual fluorescence and remarkably long‐lived phosphorescence on the millisecond timescale in the solid state, at room temperature. Although this study was confined to CMAs of Cu and is, from our point of view, not fully consistent in the interpretation of the photophysical properties (see below), there is clear indication of great potential of this class of photoactive compounds beyond TADF. Importantly, while the origins of their photochemical behaviour are rigorously being studied,[^15^, ^16^, ^17^, ^18^] an aspect of the chemistry of CMAs (and of metal‐amido complexes in general) which has remained largely stagnant deals with their synthesis.

The fundamental point of focus in the case of metal‐amido complexes, regardless of the ancillary ligand employed, is the formation of the M−N bond. Synthetic routes leading to the vast majority of CMAs reported thus far make use of the well‐defined [MCl(L)] complexes in combination with carbazole and a strong base such as KO^*t*^Bu, NaO^*t*^Bu, or KHMDS in THF as the solvent, in order to achieve N−H metallation.[^3^, ^4^, ^5^, ^6^, ^7^, ^8^, ^9^, ^10^, ^11^, ^12^, ^13^, ^14^, ^19^] Other approaches include the early use of NaOH and NBu_4_Cl as a phase transfer catalyst in a biphasic DCM/H_2_O solvent system,^[22]^ the use of lithium amides and reaction of the latter with a [AuCl(L)] complex or a cationic gold precursor,^[23]^ as well as the use of the well‐defined [Au(OH)(IPr)] synthon and more recently a new, commercially available Au‐Aryl synthon for N−H auration in THF and benzene, respectively.^[24]^ In the case of Cu, amido complexes bearing phosphines have been synthesized using lithium amides or by using KHMDS as the base,^[25]^ while NHC‐bearing complexes have been synthesized by addition of amines to a [CuMe(NHC)] complex (Me=methyl) and later by using CsOH.^[26]^ In the case of Ag, the amine lithiation approach was recently described for the synthesis of phosphine‐stabilized Ag‐amido complexes.^[27]^ All of these synthetic routes have been described in the literature as straightforward and practical on a small scale, yet in reality these have important flaws with regard to sustainability, atom‐ and step‐economy and operational simplicity. For example, the use of strong bases requires strictly inert conditions, excluding air and moisture, regardless of the stability of the initial or final organometallic materials. Furthermore, many protocols are carried out in toxic solvents and require either two synthetic steps or the presence of a phase transfer catalyst and long reaction times, without being necessarily applicable to all ligand types and all coinage metals.

Therefore, we were intrigued by the possibility of providing a simplified and general synthetic protocol to access photoactive CMAs of Cu, Ag and Au bearing various carbene‐type ligands, studying the excited state properties of new complexes and showcasing their application in photocatalysis. As a starting point, the use of a mild, cost‐efficient and robust base should be sought to render the synthesis amenable to operationally simpler and milder conditions, as we have demonstrated in a number of recent reports.[^24b^, ^28^, ^29^]

## Results and Discussion

We began our studies by utilising the optimal reaction conditions developed for the transmetallation of organoboranes to gold,^[24b]^ [MCl(NHC)] complexes **1 a**–**3 a** and carbazole as the most frequently encountered amido framework (Entries 1, 7 and 11, Table 1). In these initial experiments, full conversion was not achieved in the case of gold, while in the case of silver the reaction also led to unidentified species which could only be removed from the final material by filtration through basic alumina. Increasing the temperature in the case of gold led to full conversion, while using acetone as the solvent proved to be the optimal choice as it leads to full conversion at ambient temperature. Using NEt_3_ as the base, or using a biphasic system, led to incomplete conversion (Entries 4–6, Table 1). DFT calculations were used to investigate the effect of different weak bases on the metallation step and a concerted N−H deprotonation/metallation with K_2_CO_3_ has the lowest kinetic barrier and was more thermodynamically favoured, thus supporting the fact that this base was experimentally optimal (see Supporting Information for details). In the case of silver, acetone also proved to be the superior solvent, leading to full conversion (Entry 9, Table 1). To provide a comparative test, the synthesis of **5 a** was performed in a glovebox using a strong base and led to a slightly lower isolated yield (Entry 10, Table 1). Finally, in the case of copper, the use of ethanol (Entry 11, Table 1) proved best while other sets of conditions proved inferior (Entries 12–16, Table 1). Note here that solvents used are reagent grade solvents and used as received.

**Table 1:** Optimization of the synthetic protocols.

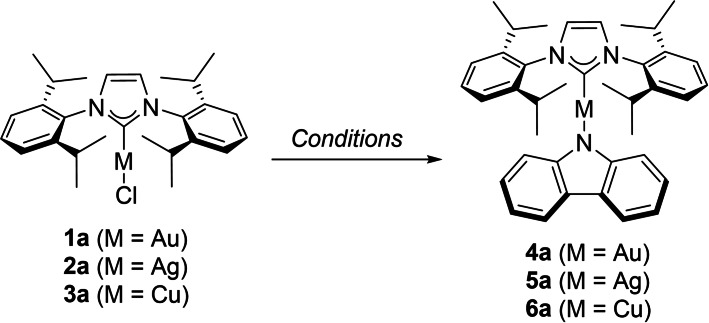				
Entry/Metal^[a]^	Solvent	Base	*T* [°C]	Conversion/Yield [%]
1/Au	EtOH	K_2_CO_3_	40	85
2/Au	EtOH	K_2_CO_3_	60	100
**3/Au**	**Acetone**	**K_2_CO_3_**	**RT** ^[b]^	**100/88**
4/Au	Acetone	NEt_3_	40	26
5/Au	Acetone	NEt_3_	60	30^[c]^
6/Au	EtOAc/H_2_O 1 : 1	K_2_CO_3_	60	80
7/Ag	EtOH	K_2_CO_3_	40	100/70^[d]^
8/Ag	THF/EtOH 4 : 1	K_2_CO_3_	40	100/72^[d]^
**9/Ag**	**Acetone**	**K_2_CO_3_**	**RT^[b]^**	**100/79**
10/Ag	THF	KO^*t*^Bu	RT	100/72^[e]^
**11/Cu**	**EtOH**	**K_2_CO_3_**	**RT^[b]^**	**100/87**
12/Cu	Acetone	K_2_CO_3_	40	93
13/Cu	Acetone	K_2_CO_3_	40	93^[c]^
14/Cu	Acetone	K_2_CO_3_	60	100^[f]^
15/Cu	*i*PrOH	K_2_CO_3_	80	100
16/Cu	Acetone	NEt_3_	40–60	0^[c]^

[a] Conditions unless otherwise noted: [MCl(IPr)] (50 mg), carbazole (1.1 equiv.), base (3.0 equiv.), 0.5 mL of solvent, 24 h. [b] Room temperature (RT) under operating conditions can range from 25 to 40 °C. [c] 48 h. [d] Impurities in the product spectra. [e] Inside a glovebox. [f] 3.5 equiv. of base.

**Scheme 1:**
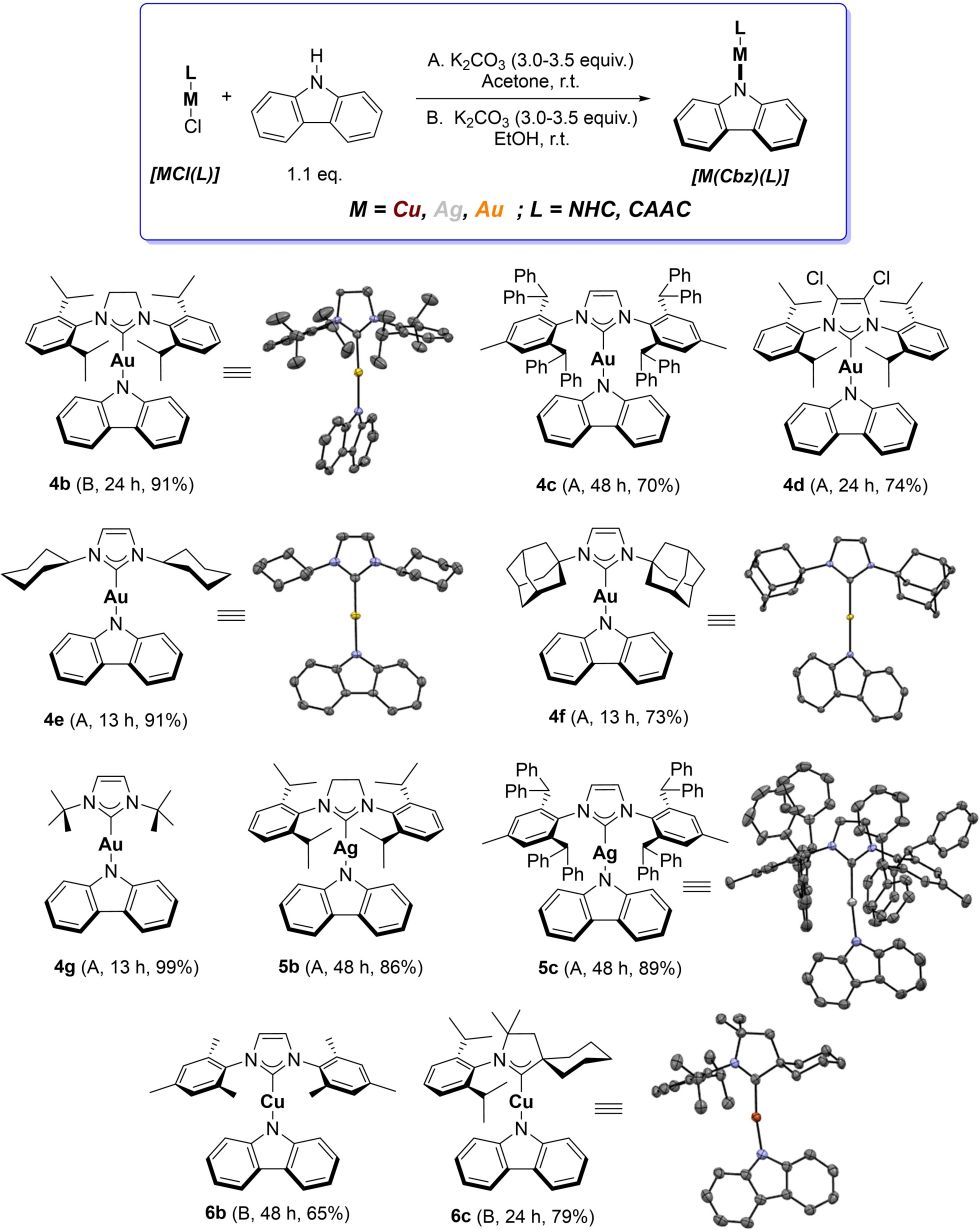
Scope of the CMA synthetic route. The X‐ray molecular structure of complexes **4 b**, **4 e**, **4 f**, **5 c** and **6 c** are presented, showing thermal displacement ellipsoids at the 50 % probability level and hydrogen atoms omitted for clarity (see Supporting Information for more detailed structural information).^[30]^

With the optimal conditions for all three coinage metals in hand, we began to explore the ligand scope of the synthetic method (Scheme 1). While investigating the scope, we found that the synthesis of each specific complex may benefit by slight variations of the optimal conditions shown in Table 1, such as alternating between ethanol and acetone as solvents. In the case of gold, a saturated backbone on the NHC did not affect the reaction and product **4 b** was obtained in high yield. When a bulkier ligand is present, unsurprisingly the reaction is slower and requires more than 24 h to reach full conversion (see Supporting Information for detailed description). Substitution on the NHC backbone did not affect the reaction, with complex **4 d** obtained under the standard conditions. Complexes bearing *N*‐alkyl substituted NHCs were also amenable to these conditions and the CMAs **4 e**–**4 g** were synthesized in high yields in only 13 h. In the case of silver, compounds **5 b** and **5 c** were successfully synthesized in high yields, and require longer reaction time, because of the increased bulk of the ligands. Interestingly, the X‐ray molecular structure of **5 c** shows that the plane of the carbazolyl fragment is forced to adopt a parallel orientation with respect to the plane of the imidazolylidene moiety, which is a unique feature among complexes with N‐aryl substituted NHC ligands reported here. This feature of **5 c** in the solid state is attributed to the steric profile of the specific NHC ligand. In the case of copper, complex **6 b** bearing a less bulky NHC was also synthesized but requires 48 h of reaction time. Finally, in order to showcase the versatility of the route, complexes beyond imidazol(in)ylidenes ligands were targeted. Compound **6 c**, bearing a cyclic (alkyl)(amino) carbene ligand was successfully synthesized using the very mild standard conditions.

**Scheme 2:**
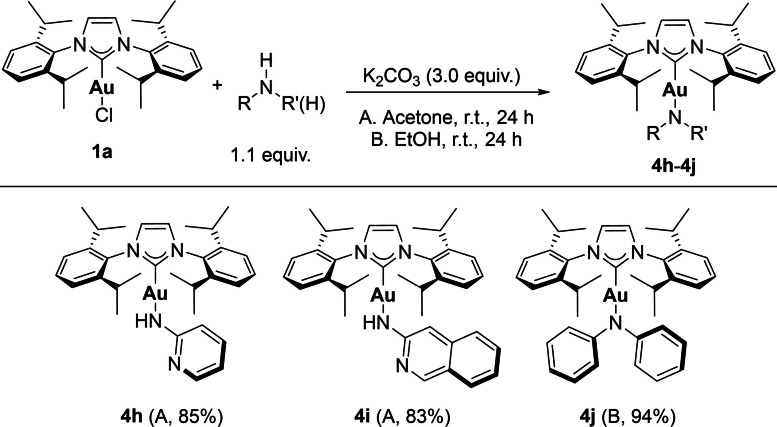
Scope of amines in the case of gold.

In order to establish whether this methodology is applicable to alternative amines, we targeted the synthesis of other gold‐NHC amido complexes (Scheme 2). Amines with significantly higher pKa values than carbazole were also metallated efficiently (**4 h**–**4 j**). In the case of 2‐aminopyridine, we found that use of other weak bases led to no conversion (see Supporting Information for details). The calculated energy profiles of the reaction, suggest that the kinetic barrier for a concerted deprotonation/metallation are similar for all other weak bases and theoretically should allow all transformations, however the case of K_2_CO_3_ is significantly more favoured thermodynamically (see Supporting Information for details) and it appears to hold a privileged position in enabling this methodology. Interestingly, the alternative use of ethanol instead of acetone proved to be essential in order to achieve full conversion in the case of complex **4 j**. This complex was synthesized on a gram scale, thus highlighting the scalability and versatility of the synthetic route.

**Scheme 3:**
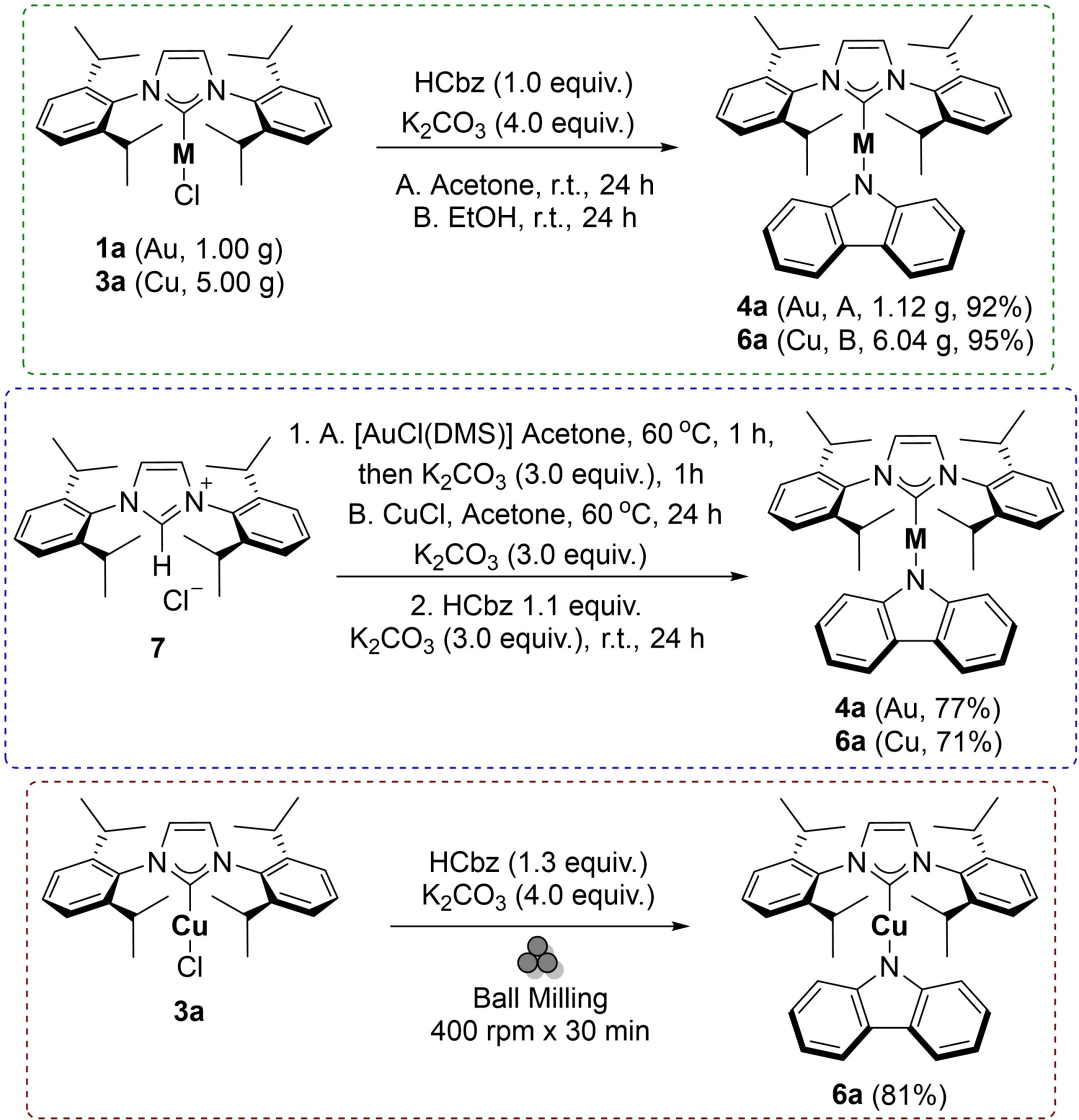
Large scale synthesis, one‐pot synthesis and mechanochemical synthesis of CMA complexes.

Scalability is an important aspect of any synthetic method. As shown in Scheme 3, the methods described here can be carried out on multigram scale, leading to products in high purity and yields, using equimolar amounts of starting materials. Furthermore, in the case of gold and copper, one‐pot syntheses of the desired CMA compounds were successfully carried out directly from the imidazolium salt and metal sources. In order to showcase potential further improvements and inspired by previous advances in mechanochemical synthesis of Cu−NHC complexes,^[29]^ we also performed a mechanochemical, solvent‐free, high‐yielding synthesis of **6 a** in a planetary ball‐mill, with a reaction time of 30 minutes (Scheme 3) to demonstrate an initial proof‐of‐concept using solvent‐free methods. This is currently being further investigated in our laboratories.

With a variety of imidazolylidene‐based CMA complexes in hand, we wanted to take a closer look at their photophysical properties and applicability in photocatalysis, for which long‐lived excited states are beneficial. It has previously been shown that [Au(MeIm)(Cbz)] exhibits dual vibronically resolved phosphorescence (λ_max_=404 and 584 nm) in the solid state, where it forms π‐stacked aggregates and intermolecular H−Au bonds.^[22a]^ Recently, [Cu(NHC)(Cbz)] complexes were reported to emit from the S_1_ state in solution, turning into dual fluorescence at 400–550 nm and remarkably ultralong phosphorescence at 580–750 nm on the millisecond timescale at RT in the solid state, which was attributed to the population of both ^1^ππ* and ^3^ππ* states localized on the carbazolyl ligand.^[19a]^

The authors indicated that intermolecular forces in the single‐crystals may be responsible for the long‐lived emission, but do not provide further details. It is important to note that the observed phosphorescence is bathochromically shifted to that reported for HCbz, KCbz and their derivatives (ca. 400–540 nm),[^7^, ^31^, ^32^] which excludes the interpretation of simple ^3^Cbz emission. Thompson et al. previously reported that some structurally related [Cu(CAAC)(Cbz)] complexes exhibit in the microcrystalline solid state ^3^Cbz emission (ca. 430–550 nm) and in addition, due to the formation of aggregates, the same vibrationally resolved phosphorescence as reported for [Cu(NHC)(Cbz)].^[7]^ Although the nature of this particular aggregate state has not been further clarified, it is feasible that its emission is due to exciton diffusion in the microcrystals, which may explain the very long lifetimes as also found for HCbz.^[32]^ However, the question remains whether persistent long‐lived triplet states of [M(NHC)(Cbz)] can be realized in solution and, more fundamentally, whether higher homologues of Cu could exert any influence on the excited state properties as found for coinage metal CAAC carbazolyl complexes.^[9]^

For the photophysical studies, the gold and silver complexes bearing IPr (**4 a**, **5 a**) and SIPr (**4 b**, **5 b**) were chosen as they provide the best comparison with the [Cu(NHC)(Cbz)] room temperature phosphorescence (RTPs)^[19a]^ and TADF emitters bearing benzimidazolylidenes.^[19b]^ An overview of all spectra and photophysical data is presented in the Supporting Information, Table S4 and Figures S5–S13.

**Figure 1:**
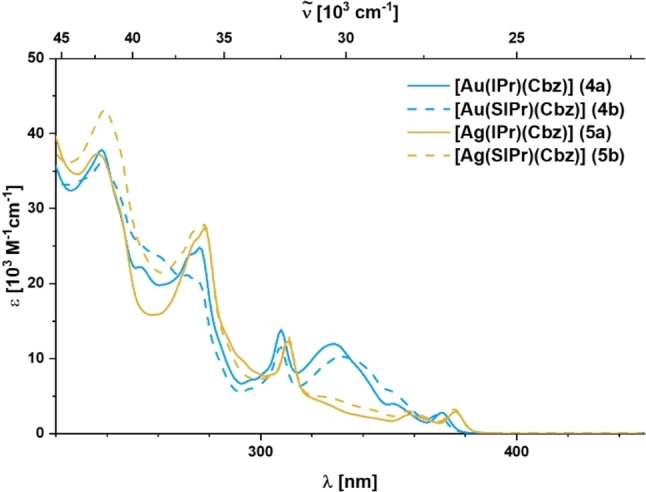
Absorption spectra of **4 a, b** and **5 a, b** in THF solution at room temperature.

The absorption spectra of CMA complexes **4 a, b** and **5 a, b** are very similar with multiple overlapping bands and shoulders between 235–380 nm (Figure 1). The high‐energy bands with maxima at 239, 278 and 311 nm are assigned to LC transitions of the carbene and carbazole ligands as the metal ions neither influence the extinction coefficients nor the energies significantly.

The absorption band between 320–350 nm is considerably broadened and the corresponding extinction coefficients of the gold complexes **4 a, b** of ca. *e*=11,000 M^−1^ cm^−1^ surpass those of their silver congeners **5 a, b** (*e*≈4,000 M^−1^ cm^−1^) by a factor of 2–3. Furthermore, this absorption band shows a minor bathochromic shift from the IPr (**4 a**, **5 a**) to the SIPr complexes (**4 b**, **5 b**) that most likely derives from the differences in acceptor strength between the NHC ligands. These features would be in line with the conclusion that it is a Cbz→NHC charge transfer (LLCT) with significant contribution from metal‐centered orbitals. The lowest energy absorption at ca. 370 nm is only weakly allowed and independent of the nature of the carbenes, but shifts with the metal ion. Thus, it is most likely a symmetry forbidden MLCT to the Cbz. Our DFT and TD‐DFT calculations generally support these assignments (see Supporting Information). However, we note that since rotation of the Cbz ligand around the M−C bond can occur with a very low energy barrier, the absorption spectrum in solution is the sum of all rotamers, each of which has different Franck‐Condon‐factors for the respective transitions, which can also shift in energy.[^4b^, ^33^, ^34^]

When investigating the emission properties, stability issues were encountered (see Supporting Information), in particular for the silver complexes **5 a, b**. In THF solution, **5 a** displays multiple emission bands with distinct vibrational progression and an overall quantum yield of *ϕ*=0.08. The fluorescence at λ_em_=343 nm (τ=13.5 ns) appears to stem from free carbazole as a photodecomposition product because the excitation spectrum of that band differs significantly from the absorption (Figure S10). Between 380–440 nm, fluorescence from the ^1^Cbz LC state occurs as a result of insufficient spin‐orbit coupling mediated by the Ag ion, leading to very slow intersystem‐crossing to the triplet moieties. In addition, very weak phosphorescence from the ^3^Cbz state is hidden underneath the fluorescence band at λ_em_=437 nm with a significantly longer lifetime of at least 1–10 μs. A similar solution behavior is observed for **5 b**, with a more pronounced ^3^Cbz LC emission with τ=332 μs, although we note that concentration and time influence the photolytic stability to a much greater extent (Figure S12). However, **4 a/b** are much more stable under photolytic conditions and, in contrast to their Cu^[19]^ and Ag congeners **5 a, b**, emit solely via phosphorescence in THF solution from a high‐energy ^3^Cbz state at λ_em_=430 nm (ϕ=0.32) with exceptionally long lifetimes of τ=332 and 266 μs, respectively. The stronger spin orbital coupling (SOC) of Au apparently facilitates ISC S_1_→T_n_ with much higher efficiency, i. e. *k*_ISC_ >10^10^ s^−1^, quenching any prompt fluorescence. Interestingly, the emissive T_1_ state is not well coupled to the ground state, resulting in very small oscillator strength and low *k*_phos_ ≈10^3^ s^−1^, which is beneficial for photocatalytic applications (see below). We also note that the nature of the carbene ligand does not seem to influence the luminescence properties, although our TD‐DFT calculations predict the ^3^LLCT state to be lowest in energy for the SIPr complexes **4 b/5 b**, while the weaker π‐acceptor IPr leads to a T_1_ state of ^3^LC character localized at the Cbz ligand for **4 a/5 a** (Supporting Information). Linearly coordinated coinage metal carbazolyl complexes are very flexible with regard to the dihedral angle between the ligand planes, which has an enormous influence on the luminescence properties.[^4b^, ^7^, ^14^, ^35^] Furthermore, the ordering of the excited states highly depends on the environment, i. e. polarity and specific solvent interactions. Therefore, we conclude that benzimidazole‐based carbenes present a borderline in terms of π‐acceptor strength to facilitate S_1_/T_1_ states of LLCT nature for TADF,[^7^, ^19b^] while weaker acceptors provide access to longer‐lived triplet states.

**Figure 2:**
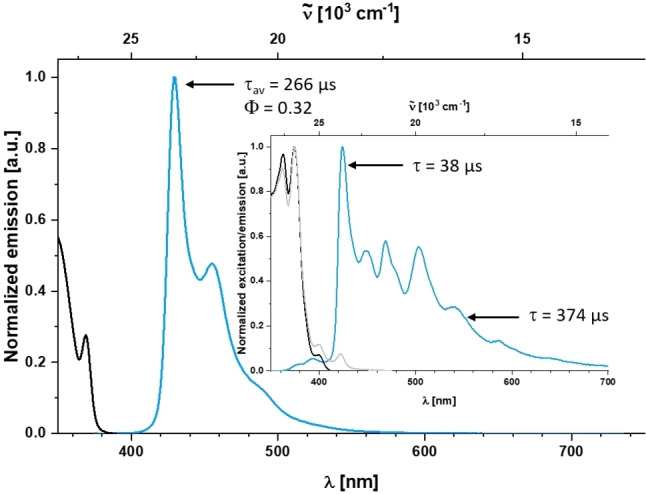
Normalized emission (blue) and excitation (black for λ_em_=430 nm, grey for λ_em_=540 nm) spectra of **4 b** in THF solution and in the solid state (inset) at room temperature.

In the solid state, we find that aggregation influences the luminescence properties of both the silver and gold complexes. Due to insufficient SOC, **5 a**, **b** show significantly broadened dual fluorescence from the LC ^1^Cbz state at λ_fl_≈390 nm with nanosecond lifetimes, indicative for *k*_ISC_<10^9^ s^−1^, and phosphorescence between λ_phos_≈480–700 nm (τ=45 and 335 μs for **5 a** and **5 b**, respectively). The triplet state emission is vibrationally not well resolved and the excitation spectra display low‐energy transitions between 400–480 nm distinctly different from the molecular absorption spectrum in solution (Figures S11 and S13). Gold complexes **4 a**, **b** retain the ^3^Cbz emission (λ_phos_=424 nm) already observed in solution. However, their respective lifetimes are significantly decreased to 74 (**4 a**) and 38 (**4 b**) μs, and a second, vibrationally resolved phosphorescence is detected with τ=885 and 374 μs, respectively (Figures 2 and S6). The excitation spectra of the two simultaneously phosphorescent states are very similar and in line with the absorption spectra in solution, although the longer‐lived emission exhibits additional excitation bands between 400–440 nm. This leads us to conclude that besides direct excitation of the aggregate state, its emission is also triggered by triplet energy transfer from the ^3^Cbz excited state of the monomers, also explaining the change in lifetime mentioned above. The implication of these findings is that the aggregate is already formed in the ground state, and its emission not merely the result of excimer formation in the solid state.

Although aggregation induced emission occurs in our Ag and Au analogs in the solid state, and despite the intermolecular interactions of **4 b** in the single crystals being very similar to those reported for [Cu(IMes/IPr)(Cbz)], we do not observe such remarkably long‐lived RTP as reported for those Cu complexes.^[19a]^ But bearing in mind that it has also been reported for [Au(MeIm)(Cbz)] and some [Cu(CAAC)(Cbz)] complexes,[^7^, ^22a^] and that the aggregate emission of **4 a, b** and **5 a, b** is energetically different, it is possible that the specific crystalline environment or defect sites are responsible for the formation of very long‐lived charge‐separated excitons as recently reported for HCbz.^[32]^

Complex [Au(SIPr)(Cbz)] (**4 b**) demonstrated the highest photostability, high‐energy phosphorescence in the blue‐green range with a remarkable long lifetime in solution, and showed an indication to undergo energy transfer, and was thus chosen as triplet state sensitizer for a proof‐of‐concept photocatalysis reaction. We selected (E,E’)‐dicinnamyl ether as the substrate for a [2+2] cycloaddition via triplet energy transfer^[36]^ upon irradiation at 365 nm with a 18 W LED, where **4 b** still offers a reasonable extinction coefficient and absorption by common borosilicate glass does not appear to be a major issue. Both catalyst loading and irradiation time were varied to screen the performance limit of **4 b** (Table 2, Entries 1–5) in this initial photocatalytic study.

**Table 2:** Optimization and control studies for the photocatalytic [2+2] cycloaddition.

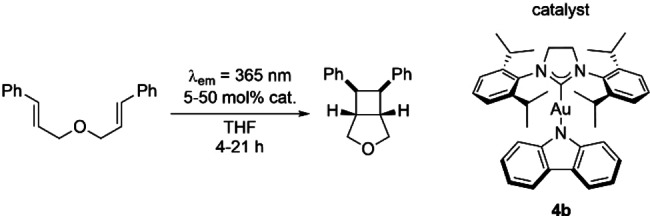			
Entry	Catalyst Load^[a]^ [mol %]	Time [h]	Conversion^[b]^ [%]
1	50	21	>99
2	10	21	>99
3	5	21	>99
4	10	4	>99
5	5	4	>99
6	none	21	12
7^[c]^	10	21	0

[a] Catalyst loading for entry 1 was simply weighed, for Entries 3–7, a stock solution in THF was used. [b] Conversions were determined from the ratio of the product and reactant signals in the ^1^H NMR spectrum. [c] Control reaction performed in the dark.

Full conversion to the desired cycloaddition product was only achieved with **4 b** acting as a photocatalyst. While some conversion (12 %) was observed in the absence of **4 b** after irradiation for 21 h (entry 6), most likely the result of direct excitation of the triplet state of (E,E’)‐dicynnamyl ether, the reaction was completely inhibited when kept in the dark. Overall, **4 b** exhibits remarkable efficiency for a non‐optimized photocatalyst in this chosen [2+2] cycloaddition reaction. Further work is ongoing to test the potential of **4 b** and related complexes as photocatalysts.

## Conclusion

We have demonstrated for the first time that CMAs derived from copper‐, silver‐ and gold‐NHC complexes can be accessed via simple and sustainable synthetic routes. This methodology makes use of a mild base, K_2_CO_3_, which proved to be superior to other mild bases both experimentally and computationally. The issue of solvent use was addressed in the deployment of green solvents (acetone and ethanol) in reactions performed in classical batch mode and under solventless conditions using a mechanochemical approach, which is also showcased for the first time for CMA synthesis. The synthesis of CMAs displays a wide scope with regard to both the NHC ligand and the amine used, while it is scalable and does not require elaborate experimental setups and/or handling. The potential of this advance is evident not only by the simplicity and mild conditions used, but also by the examples of CMA synthesis directly from imidazolium salts, metal sources and carbazole in a one‐pot fashion. The combination of desirable reaction solvents, mild reagents, open‐to‐air conditions and wide scope are expected to significantly aid in the construction of diverse CMA libraries for photochemical and other applications. Considering the original aim of the photophysical and photocatalytic survey, two important insights are provided by this study: 1) the influence of the transition metal on the emission properties of NHC‐M‐Cbz is perceivable (enhanced SOC to facilitate ISC, ultimately giving pure phosphorescence for Au), but within the boundaries of this particular ligand system, highly efficient emitters rivalling analogous CAAC or MAC complexes are not obtainable solely by changing the metal and 2) minor variations of the carbene ligand are far more consequential, as a comparison with the corresponding benzimidazole‐based carbene complexes reported by Thompson and co‐workers^[19b]^ illustrates, however, the introduction of the 4d and, particularly, 5d TM appears to be highly beneficial to fine‐tune photophysical properties. This was confirmed by the impressive performance of the fairly simple gold complex [Au(SIPr)(Cbz)] (**4 b**) in a proof‐of‐concept photocatalysis reaction, where complete conversion was achieved even at relatively low catalyst loadings ( 5 mol%) and short reaction times (4 h). This finding may provide a new perspective on the wide‐ranging applicability of CMAs, especially with highly versatile carbenes such as 5‐membered imidazole‐based NHCs, that have attracted much less attention in studies of CMA complexes due to their lackluster performance in OLED emitter materials.

## Conflict of interest

The authors declare no conflict of interest.

## Supporting information

As a service to our authors and readers, this journal provides supporting information supplied by the authors. Such materials are peer reviewed and may be re‐organized for online delivery, but are not copy‐edited or typeset. Technical support issues arising from supporting information (other than missing files) should be addressed to the authors.

Supporting Information
