# Supplementary material for: Simple Synthetic Routes to Carbene‐M‐Amido (M=Cu, Ag, Au) Complexes for Luminescence and Photocatalysis Applications
Source: Chemistry. 2021 Jun 28;27(46):11904–11. doi: 10.1002/chem.202101476 (PMC8456869; doi:10.1002/chem.202101476)
Supplement: Supplementary file 1 — Supporting Information [file CHEM-27-11904-s001.pdf]

Chemistry–A European Journal

Supporting Information

Simple Synthetic Routes to Carbene-M-Amido (M=Cu, Ag, Au) Complexes for Luminescence and Photocatalysis Applications

Nikolaos V. Tzouras, Ekaterina A. Martynova, Xinyuan Ma, Thomas Scattolin, Benjamin Hupp, Hendrik Busen, Marina Saab, Ziyun Zhang, Laura Falivene, Gianmarco Pisanò, Kristof Van Hecke, Luigi Cavallo, Catherine S. J. Cazin, Andreas Steffen,* and Steven P. Nolan*

Table of Contents

Experimental Procedures ... 1
General information ... 1
Computational details ... 1
Photophysical measurements and photocatalysis ... 2
Synthetic procedures ... 2
Scope of ligands and coinage metals ... 2
Scope of amines ... 9
Results and Discussion ... 11
Weak base tests and DFT calculations ... 11
Molecular structures of complexes 4b, 4e, 4f, 5a, 5c, 6a and 6c ... 15
Photophysical properties of 4a,b and 5a,b and analytical data for photocatalysis ... 17
References ... 26
NMR Spectra ... 27
Cartesian Coordinates ... 49

Experimental Procedures

General information

All reactions were carried out in air, unless otherwise noted. Solvents and all other reagents were purchased and used as received without further purification unless otherwise stated. [AuCl(DMS)] was prepared from $HAuCl_4 \cdot nH_2O$, which was supplied by Umicore. All [M(NHC)Cl] complexes were synthesized according to known procedures.[1-3] Carbazole 96% (Cbz used as the acronym) was used as supplied by Acros Organics. Purification of compounds by filtration was performed using basic alumina or celite purchased from Sigma Aldrich, or syringe membrane filters purchased from Carl Roth. Unless otherwise noted, absolute ethanol, reagent grade acetone, deionized water and freshly crushed, anhydrous bases were used. Potassium tert-butoxide was purchased from Sigma Aldrich and was used in a glove box (Innovative Technology) with a dry argon atmosphere, in combination with dry and degassed THF prepared according to literature procedures. ^1H, ^{13}C-{^1H} Nuclear Magnetic Resonance (NMR) spectra were recorded on a Bruker Avance 400 Ultrashield, Bruker Avance 300 Ultrashield or a Bruker Avance 500 spectrometer at 298 K using the residual solvent peak as reference ($CDCl_3$: δ_H = 7.26 ppm, δ_C = 77.16 ppm; CD_2Cl_2: δ_H = 5.32 ppm, δ_C = 54.00 ppm, Acetone-d_6: δ_H = 2.05 ppm, δ_C = 29.92 ppm). Peaks are assigned as: s (singlet), d (doublet), t (triplet), h (heptuplet) and m (multiplet). All chlorinated solvents (including the deuterated ones) were neutralized prior to use by filtration through dried basic alumina, as the metal amido complexes were found to be sensitive to traces of HCl in these solvents. Elemental analyses were performed at Université de Namur, rue de Bruxelles, 55 B-5000 Namur, Belgium.

Computational details

For the N-H metallation reactions, geometries were optimized with the Gaussian09 package[4] at the PBE0-D3 level of theory.[5] The standard split-valence basis set with a polarization function of Ahlrichs and coworkers was used for H, C, N, O and Cl atoms (SVP keyword in Gaussian)[6] while the quasi relativistic small-core Stuttgart effective core potential (ECP) was used for Au (SDD keyword in Gaussian09). The reported free energies have been obtained via single point energy calculations with the triple-ζ basis set of Ahlrichs for main group atoms atoms (TZVP keyword in Gaussian09).[7] Solvent effects, acetone, was included using the PCM method.[8] To this PBE0-D3/TZVP electronic energy in solvent, zero point and thermal corrections were added from the gas-phase frequency calculations at the PBE0-D3/SVP level.

Calculations for **4a** and **5a/b** were performed with the ORCA 4.2.1 program suite with tight SCF convergence criteria.[9] Geometry optimizations (gas-phase) were carried out with the PBE[5a, 10] functional as implemented in ORCA, and a frequency analysis ensuring that the optimized structures correspond to global energy. The def2-TZVP[6,11] basis set was used for all atoms together with the auxiliary basis set SARC/J[12] in order to accelerate the computations within the framework of RI approximation. Relativistic effects were accounted for by employing the ZORA[12c] method, and van der Waals interactions have been considered by an empirical dispersion correction (Grimme-D3BJ).[13] TD-DFT calculations for the first 20 singlet and triplet excited states of **4a** and **5a/b** were performed with the hybrid version of the previous functional, i.e. PBE0, using the def2-SVP[6,11] basis set, including a conductor-like polarizable continuum model (CPCM) for CH_2Cl_2. Representations of electronic transition differences at isovalues of 0.0015 were produced with orca_plot as provided by ORCA 4.2.1 and with USCF Chimera[14].

Photophysical measurements and photocatalysis

All measurements were performed under argon and rigorous exclusion of moisture and air. For solid state measurements, precipitated or microcrystalline material has been used. UV-visible absorption spectra were obtained on an Agilent Cary 5000 spectrophotometer using standard 1 cm path length quartz cells. Excitation and emission spectra were recorded on an Edinburgh Instrument FLS1000 spectrometer, equipped with a 450 W Xenon arc lamp, double monochromators for the excitation and emission pathways, and a red-sensitive photomultiplier (PMT-980) as detector. The excitation and emission spectra were collected at right angles to the excitation source and were corrected using the standard corrections supplied by the manufacturer for the spectral power of the excitation source and the sensitivity of the detector. The quantum yields were measured by use of an integrating sphere with an Edinburgh Instrument FLS1000 spectrometer. The luminescence lifetimes were measured either using a µF2 pulsed 60 W Xenon microsecond flashlamp, with a repetition rate between 10-100 Hz (depending on the time range), and a multichannel scaling (MCS) module or via time-correlated single photon counting (TCSPC) using pulsed ps laser diodes (40 µW).

For photocatalysis experiments, an EvoluChem^{TM} PhotoRedOx Box by HepatoChem (see below), equipped with an EvoluChem^{TM} LED 365PF (365 nm, 18 W, 9 mW/cm²), was used. Reaction mixtures were prepared in 2 ml GC vials with magnetic stir bars using 0.5 mL of a solution of (E,E')-dicinnamyl ether (40 mg/mL in THF), adding a corresponding volume of the Au complex **4b** in THF (10 mg/mL) and pure THF up to a volume of 1.5 mL under inert conditions, then irradiated for the time given in Table 2. The organic layer was concentrated *in vacuo* and conversion was determined after dissolving the residue in $CDCl_3$ by recording ^1H NMR spectra on a Agilent DD2 – 500 MHz spectrometer equipped with a RT 5 mm Triple Resonance Sample Head, referenced to TMS (0.0 ppm). To avoid further irradiation by stray light, the vials and NMR tubes were covered with aluminium foil when handled outside the photoreactor.

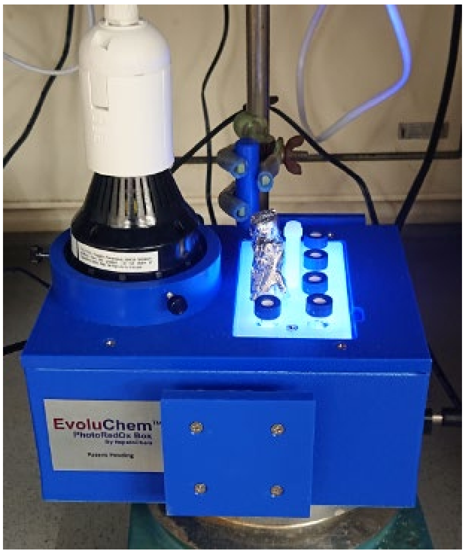

Synthetic procedures

Scope of ligands and Coinage metals

Synthesis of [*N*,*N*-Bis(2,6-diisopropylphenyl)imidazol-2-ylidene](9H-carbazol-9-yl)gold(I) [Au(IPr)Cbz] (4a):

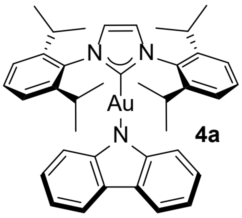

4a

Procedure A (small scale): A 4 mL scintillation vial equipped with a septum cap and a stirring bar was charged with [Au(IPr)Cl] (50 mg, 0.081 mmol), carbazole (14.1 mg, 0.085 mmol, 1.05 equiv.), K_2CO_3 (33.4 mg, 0.242 mmol, 3 equiv.) and acetone (0.5 mL). The reaction mixture was stirred at room temperature for 24 hours. The solvent was removed under vacuum and purification of the product was carried out by filtration through a millipore membrane filter with THF (4 mL). Evaporation of the solvent, washing with pentane (3x3 mL) and drying under high vacuum afforded the product as a white powder in 88% yield (54 mg, 0.071 mmol).

Procedure A (large scale): A 40 mL scintillation vial equipped with a septum cap and a stirring bar was charged with [Au(IPr)Cl] (1.00 g, 1.61 mmol,), carbazole (96%, 0.281 g, 1.61 mmol,1.00 equiv.), K_2CO_3 (0.890 g, 6.44 mmol, 4 equiv.) and acetone (15 mL). Full

conversion had been achieved in 24 hours of stirring at room temperature, as judged by NMR analysis of an aliquot. After concentrating the reaction mixture to dryness, purification of the product was carried out by filtration through a pad of celite with THF (60 mL). Evaporation of the solvent under vacuum, washing with diethyl ether (3x30 mL) on a frit and drying under high vacuum afforded the product as a white, microcrystalline solid in 92% yield (1.12 g, 1.48 mmol).

Procedure B (one pot): A 4 mL scintillation vial equipped with a septum cap and a stirring bar was charged with [Au(DMS)Cl] (30 mg, 0.10 mmol), IPr•HCl (43.3 mg, 0.10 mmol, 1.0 equiv.) and acetone (0.6 mL). After stirring the solution at 60 °C for 1 hour, K_2CO_3 (42.2 mg, 0.31 mmol, 3 equiv.) was added in one portion and the mixture was stirred at 60 °C for 1 hour. The reaction was cooled to room temperature and carbazole (18.7 mg, 0.11 mmol,1.1 equiv.) was added, followed by K_2CO_3 (42.2 mg, 0.31 mmol, 3 equiv.). The mixture was left stirring at room temperature for 24 hours. The solvent was evaporated under vacuum and the residue was taken up in THF (5 mL) and filtered through an alumina plug (1 cm).). Evaporation of the solvent under vacuum, washing with diethyl ether (3x3 mL) and drying under high vacuum afforded the product as a white, microcrystalline solid in 77% yield (59 mg, 0.08 mmol).

^{1}H NMR (400 MHz, CD_2Cl_2): δ (ppm) = 7.88 (ddd, *J* = 7.7, 1.2, 0.7 Hz, 2H), 7.65 (t, *J* = 7.8 Hz, 2H), 7.44 (d, *J* = 7.8 Hz, 4H), 7.35 (s, 1H), 7.03 (ddd, *J* = 8.2, 7.0, 1.3 Hz, 2H), 6.86 (ddd, *J* = 7.9, 7.1, 1.0 Hz, 2H), 6.73 (dt, *J* = 8.2, 0.8 Hz, 2H), 2.72 (hept, *J* = 6.9 Hz, 4H), 1.38 (d, *J* = 6.9 Hz, 12H), 1.29 (d, *J* = 6.9 Hz, 12H).

^{13}C {^{1}H} NMR (101 MHz, CD_2Cl_2): δ (ppm) = 179.2, 149.9, 146.7, 134.9, 131.2, 124.8, 124.0, 123.9, 123.9, 119.6, 116.2, 114.0, 29.5, 24.7, 24.5.

Analytical data obtained are in agreement with reported values.[15]

Synthesis of [*N,N*-Bis(2,6-diisopropylphenyl)imidazolin-2-ylidene](9H-carbazol-9-yl)gold(I) [Au(SIPr)Cbz] (4b):

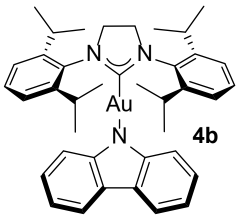

4b

A vial was charged, under air, with 50.0 mg of [Au(SIPr)Cl] (0.081 mmol), 33.2 mg of K_2CO_3 (0.240 mmol, 3 equiv.), 14.1 mg of carbazole (0.098 mmol, 1.05 equiv.) and the solids were suspended in EtOH (0.5 mL). The reaction mixture was stirred at room temperature for 24 hours. After this time the solvent was removed in vacuo and THF was added (4 mL). The mixture was filtered through aluminium oxide (basic), the solvent was removed in vacuo and the resulting solid was washed with pentane (3 ×3 mL). Then the mixture was filtered and the desired compound was obtained as a white solid in 91% yield (47 mg, 0.076 mmol).

^{1}H NMR (400 MHz, CD_2Cl_2): δ (ppm) = 7.89 – 7.84 (m, 2H, $CH_{Ar\ cbz}$), 7.60 (t, *J* = 7.8 Hz, 2H, CH_{Ar}), 7.41 (d, *J* = 7.8 Hz, 4H, CH_{Ar}), 7.03 – 6.93 (m, 2H, $CH_{Ar\ cbz}$), 6.88 – 6.81 (m, 2H, $CH_{Ar\ cbz}$), 6.52 – 6.44 (m, 2H, $CH_{Ar\ cbz}$), 4.19 (s, 4H, $CH_{2\ imid}$), 3.22 (hept, *J* = 6.9 Hz, 4H, C***H***$(CH_3)_2$), 1.42 (d, *J* = 6.9 Hz, 12H, CH(C***H***$_3)_2$), 1.39 (d, *J* = 6.9 Hz, 12H, CH(C***H***$_3)_2$).

^{13}C{^{1}H} NMR (101 MHz, CD_2Cl_2): δ (ppm) = 200.4 (C-Au), 149.9 (C-N_{cbz}), 147.9 (***C***-CH$(CH_3)_2$), 135.0, 130.4 (CH_{Ar}), 125.1 (CH_{Ar}), 124.1, 123.9 ($CH_{Ar\ cbz}$), 119.6 ($CH_{Ar\ cbz}$), 116.2 ($CH_{Ar\ cbz}$), 113.9 ($CH_{Ar\ cbz}$), 54.2 ($CH_{2\ imid}$), 29.6 (***C***H$(CH_3)_2$), 25.3 (CH(***C***H$_3)_2$), 24.6 (CH(***C***H$_3)_2$).

Elemental analysis calcd (%) for $C_{39}H_{46}AuN_3$: C, 62.14; H, 6.15; N, 5.57; found: C 62.13; H 6.12; N 5.56.

Synthesis of [*N,N*-Bis(2,6-bis(diphenylmethyl)-4-methylphenyl)imidazol-2-ylidene](9H-carbazol-9-yl)gold(I) [Au(IPr*)Cbz] (4c):

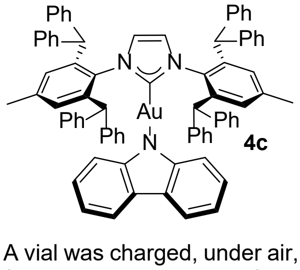

4c

A vial was charged, under air, with 50.0 mg of [Au(IPr*)Cl] (0.044 mmol), 18.2 mg of K_2CO_3 (0.132 mmol, 3 equiv.), 7.7 mg of carbazole (0.046 mmol, 1.05 equiv.) and the solids were suspended in acetone (0.5 mL). The reaction mixture was stirred at room temperature for 48 hours. After this time the solvent was removed in vacuo and THF was added (4 mL). The mixture was filtered through micro filter and the solvent was removed in vacuo. Diethyl ether was added to the residue and the desired complex was precipitated with pentane. The solvents were decanted and pentane (3 ×3 mL) was used to wash the product which was obtained as a white solid in 70% yield (39.5 mg, 0.031 mmol).

^{1}H NMR NMR (300 MHz, Acetone-*d*$_6$): δ (ppm) = 8.22 – 7.90 (m, 2H, $CH_{Ar\ cbz}$), 7.36 – 7.21 (m, 12H), 7.20 – 7.13 (m, 8H, CH_{Ar}), 7.09 (s, 4H, $CH_{Ar\ IPr*}$), 7.04 – 6.90 (m, 18H, CH_{Ar}), 6.88 – 6.79 (m, 8H), 6.27 (s, 2H, CH_{imid}), 5.52 (s, 4H, $CHPh_2$), 2.34 (s, 6H, CH_3).

^{13}C NMR (75 MHz, Acetone-*d*$_6$): δ (ppm) = 178.9 (C-Au), 150.6 (C-N_{cbz}), 143.6, 143.6, 142.6, 141.1, 135.3, 131.0, 130.5, 130.3, 129.4, 129.1, 127.7, 127.3, 125.0, 124.8 (CH_{imid}), 124.2, 119.9 ($C_{Ar\ cbz}$), 116.6, 114.9, 52.3 ($CHPh_2$), 21.8 (CH_3).

Elemental analysis calculated (%) for $C_{81}H_{64}AuN_3$: C, 76.22; H, 5.05; N, 3.29; found: C 75.84; H 5.11; N 3.09.

Synthesis of [4,5-dichloro-*N,N*-Bis(2,6-diisopropylphenyl)imidazol-2-ylidene](9H-carbazol-9-yl)gold(I) [Au(IPr)Cbz] (4d):

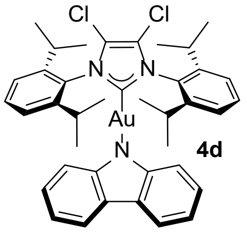

4d

A 20 mL scintillation vial equipped with a septum cap and a stirring bar was charged with [Au(IPr^{Cl})Cl] (200 mg, 0.290 mmol), carbazole (53.3 mg, 0.318 mmol, 1.1 equiv.), K_2CO_3 (140 mg, 1.01 mmol, 3.5 equiv.) and acetone (3 mL). The reaction mixture was stirred at room temperature for 24 hours. The solvent was removed under vacuum and purification of the product was carried out by filtration through a syringe membrane filter with THF (4 mL). The filtrate was additionally pushed through a basic alumina plug (1 cm) using THF (5 mL) Evaporation of the solvent, washing with cold diethyl ether (3x5 mL) and drying under high vacuum afforded the product as a white powder in 74% yield (170 mg, 0.207 mmol). This compound was found to be substantially soluble in diethyl ether (necessary for removing the excess of carbazole), however most of the amount that is dissolved can be recovered as it precipitates at lower temperatures (0-4 °C).

^{1}H NMR (400 MHz, $CDCl_3$): δ (ppm) = 7.95 (d, *J* = 7.7 Hz, 2H, $H_{Ar\text{-}cbz}$), 7.70 (t, *J* = 7.8 Hz, 2H, $H_{Ar(IPr^{Cl})}$), 7.45 (d, *J* = 7.8 Hz, 4H, $H_{Ar(IPr^{Cl})}$), 7.10 – 7.01 (m, 2H, $H_{Ar\text{-}cbz}$), 6.94 – 6.88 (m, 2H, $H_{Ar\text{-}cbz}$), 6.65 (d, *J* = 8.1 Hz, 2H, $H_{Ar\text{-}cbz}$), 2.61 (hept, *J* = 6.8 Hz, 4H, $C\mathit{H}(CH_3)_{2(IPr^{Cl})}$), 1.37 (d, *J* = 6.9 Hz, 12H, $CH(C\mathit{H}_3)_{2(IPr^{Cl})}$), 1.32 (d, *J* = 6.9 Hz, 12H, $CH(C\mathit{H}_3)_{2(IPr^{Cl})}$).

^{13}C {^{1}H} NMR (101 MHz, $CDCl_3$): δ (ppm) = 179.3 (C-Au), 149.4 (C-N_{cbz}), 146.7(N$\mathit{C}_{Ar(IPr^{Cl})}$), 131.7 ($CH_{Ar(IPr^{Cl})}$), 131.6 (N$\mathit{C}_{imid(IPr^{Cl})}$), 124.8 ($CH_{Ar(IPr^{Cl})}$), 123.8 ($C_{Ar\text{-}cbz}$), 123.5 ($CH_{Ar\text{-}cbz}$), 119.5 ($CH_{Ar\text{-}cbz}$), 119.1 ($C_{Ar\text{-}cbz}$), 115.9 ($CH_{Ar\text{-}cbz}$), 113.5 ($CH_{Ar\text{-}cbz}$), 29.5 ($\mathit{C}H(CH_3)_{2(IPr^{Cl})}$), 24.7 ($CH(\mathit{C}H_3)_{2(IPr^{Cl})}$), 23.8 ($CH(\mathit{C}H_3)_{2(IPr^{Cl})}$).

Elemental analysis calcd (%) for $C_{39}H_{42}AuCl_2N_3$: C 57.08, H 5.16, N 5.12; found: C 56.64, H 4.78, N 5.26.

Synthesis of [*N,N*-Bis(cyclohexyl)imidazol-2-ylidene] (9H-carbazol-9-yl)gold(I) [Au(ICy)Cbz] (4e):

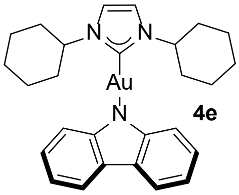

4e

A vial was charged, under air, with 50.0 mg of [Au(ICy)Cl] (0.108 mmol), 44.6 mg of K_2CO_3 (0.323 mmol, 3 equiv.), 18.9 mg of carbazole (0.113 mmol, 1.05 equiv.) and the solids were suspended in acetone (0.5 mL). The reaction mixture was stirred at room temperature for 13 hours. After this time the solvent was removed in vacuo and THF was added (4 mL). The mixture was filtered through a syringe membrane filter, the solvent was evaporated under vacuum and the resulting solid was washed with pentane (3 ×3 mL). Then the mixture was filtered on a frit using pentane and the desired compound was obtained as a white solid in 91% yield (58.8 mg, 0.098 mmol) after drying under vacuum.

^{1}H NMR (500 MHz, CD_2Cl_2) δ (ppm) = 8.08 (d, *J* = 7.7 Hz, 2H, $CH_{Ar\ cbz}$), 7.75 (d, *J* = 8.1 Hz, 2H, $CH_{Ar\ cbz}$), 7.33 (t, *J* = 7.5 Hz, 2H, $CH_{Ar\ cbz}$), 7.09 – 6.99 (m, 4H, CH), 4.77 (tt, *J* = 12.1, 3.7 Hz, 2H, N-CH), 2.27 (d, *J* = 11.5 Hz, 4H, CH_2), 1.98 (d, *J* = 13.7 Hz, 4H, CH_2), 1.86 – 1.72 (m, 6H, CH_2), 1.63 – 1.55 (m, 4H, CH_2), 1.38 – 1.24 (m, 2H, CH_2).

^{13}C NMR (126 MHz, CD_2Cl_2) δ (ppm) = 172.5 (C-Au), 150.3 (C-N_{cbz}), 124.5 (***C***-CN_{cbz}), 124.3 ($CH_{Ar\ cbz}$), 120.1 ($CH_{Ar\ cbz}$), 117.9 (CH_{imid}), 116.6 ($CH_{Ar\ cbz}$), 114.2 ($CH_{Ar\ cbz}$), 61.9 (N-CH_{Cy}), 34.8 (CH_2), 26.2 (CH_2), 25.8 (CH_2).

Elemental analysis calcd (%) for $C_{27}H_{32}AuN_3$: C, 54.45; H, 5.42; N, 7.06; found: C, 54.31; H, 5.48; N, 7.06.

Synthesis of [*N,N*-Bis(adamantyl)imidazol-2-ylidene](9H-carbazol-9-yl)gold(I) [Au(IAd)Cbz] (4f):

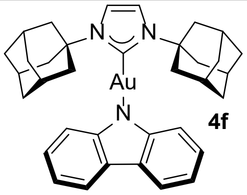

4f

A vial was charged, under air, with 50.0 mg of [Au(IAd)Cl] (0.088 mmol), 36.5 mg of K_2CO_3 (0.264 mmol, 3 equiv.), 15.4 mg of carbazole (0.092 mmol, 1.05 equiv.) and the solids were suspended in acetone (0.5 mL). The reaction mixture was stirred at room temperature for 13 hours. After this time the solvent was removed in vacuo and dichloromethane was added (4 mL). The mixture was filtered through a syringe membrane filter and after evaporation of the solvent, the resulting solid was washed with diethyl ether (1 mL). Then the mixture was filtered and the desired compound was obtained as a white solid in 74% yield (45.5 mg, 0.065 mmol) after drying under vacuum.

^{1}H NMR (500 MHz, CD_2Cl_2) δ (ppm) = 8.08 (d, *J* = 7.7 Hz, 2H, $CH_{Ar\ cbz}$), 7.72 (d, *J* = 8.1 Hz, 2H, $CH_{Ar\ cbz}$), 7.35 – 7.27 (m, 2H, $CH_{Ar\ cbz}$), 7.20 (s, 2H, $CH_{2\ imid}$), 7.08 – 6.96 (m, 2H, $CH_{Ar\ cbz}$), 2.75 (d, *J* = 2.6 Hz, 12H, CH_2), 2.31 (s, 6H, CH_2), 1.80 (q, *J* = 12.5 Hz, 12H, CH_2).

^{13}C NMR (101 MHz, CD_2Cl_2) δ (ppm) = 170.4 (s, C-Au), 150.2 (s, C-N_{cbz}), 124.5 (s, C_{cbz}), 124.2 (s, $CH_{Ar\ cbz}$), 120.0 (s, $CH_{Ar\ cbz}$), 116.4, 116.3, 114.0 (s, $CH_{Ar\ cbz}$), 59.9 (s, N-C_{Ad}), 44.9 (s, CH_2), 36.4 (s, CH_2), 30.7 (s, CH_2).

Elemental analysis calculated (%) for $C_{35}H_{40}AuN_3$: C, 60.08; H, 5.76; N, 6.01; found: C, 60.39; H, 5.50; N, 5.93.

Synthesis of [*N,N*-Bis(*tert*-butyl)imidazol-2-ylidene] (9H-carbazol-9-yl)gold(I) [Au(I^{t}Bu)Cbz] (4g):

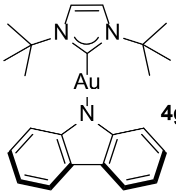

4g

A vial was charged, under air, with 50.0 mg of [Au(I^{t}Bu)Cl] (0.121 mmol), 50.2 mg of K_2CO_3 (0.363 mmol, 3 equiv.), 21.3 mg of carbazole (0.127 mmol, 1.05 equiv.) and the solids were suspended in acetone (0.5 mL). The reaction mixture was stirred at room temperature for 13 hours. After this time the solvent was removed in vacuo and dichloromethane was added (4 mL). The mixture was filtered through a syringe membrane filter and after evaporation of the solvent, the resulting was washed with diethyl ether (1 mL). Then the mixture was filtered and the desired compound was obtained as a white solid in 99% yield (65.1 mg, 0.120 mmol) after drying under vacuum.

^{1}H NMR (400 MHz, CD_2Cl_2): δ (ppm) = 8.13 – 8.04 (m, 2H, $CH_{Ar\ cbz}$), 7.78 – 7.66 (m, 2H, $CH_{Ar\ cbz}$), 7.37 – 7.28 (m, 2H, $CH_{Ar\ cbz}$), 7.17 (s, 2H, $CH_{2\ imid}$), 7.08 – 7.00 (m, 2H, $CH_{Ar\ cbz}$), 2.03 (s, 18H, CH_3).

^{13}C NMR (101 MHz, CD_2Cl_2): δ (ppm) = 172.2 (s, C-Au), 150.3 (s, C-N_{cbz}), 124.5 (s, C_{cbz}), 124.3 (s, $CH_{Ar\ cbz}$), 120.0 (s, $CH_{Ar\ cbz}$), 117.2 (s), 116.5 (s), 114.1 (s, $CH_{Ar\ cbz}$), 59.5 (s, ***C***-CH_3), 32.2 (s, CH_3).

Elemental analysis calcd (%) for $C_{23}H_{28}AuN_3$: C, 50.83; H, 5.19; N, 7.73; found: C 50.82; H 5.11; N 7.50.

Synthesis of [*N,N*-Bis(2,6-diisopropylphenyl)imidazol-2-ylidene](9H-carbazol-9-yl)silver(I) [Ag(IPr)Cbz] (5a):

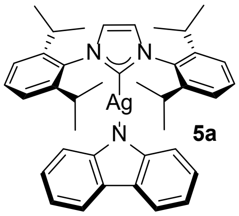

5a

Procedure A (small scale): A 4 mL scintillation vial equipped with a septum cap and a stirring bar was charged with [Ag(IPr)Cl] (50 mg, 0.094 mmol), carbazole (16.5 mg, 0.099 mmol, 1.1 equiv.), K_2CO_3 (38.9 mg, 0.282 mmol, 3 equiv.) and acetone (0.6 mL). The reaction mixture was stirred at room temperature for 24 hours. The solvent was removed under vacuum and purification of the product was carried out by filtration through an alumina plug (1 cm) with THF (4 mL). Evaporation of the solvent, washing with diethyl ether (3x3 mL) and drying under high vacuum afforded the product as an off-white powder in 79% yield (49mg, 0.074 mmol).

Procedure A (200 mg scale): A 20 mL scintillation vial equipped with a septum cap and a stirring bar was charged with [Ag(IPr)Cl] (200 mg, 0.376 mmol,), carbazole (69.13 mg, 0.413 mmol,1.1 equiv.), K_2CO_3 (182 mg, 1.31 mmol, 3.5 equiv.) and acetone (3 mL). Full conversion had been achieved in 24 hours of stirring at room temperature, as judged by NMR analysis of an aliquot. After concentrating the reaction mixture to dryness, purification of the product was carried out by filtration through an alumina plug (1 cm) with THF (6 mL). Evaporation of the solvent under vacuum, washing with diethyl ether (3x5 mL) on a frit and drying under high vacuum afforded the product as an off-white, microcrystalline solid in 87% yield (217 mg, 0.327 mmol).

Procedure B (glove box): In the glove box, a 4 mL scintillation vial equipped with a septum cap and a stirring bar was charged with [Ag(IPr)Cl] (50 mg, 0.094 mmol), carbazole (16.5 mg, 0.099 mmol, 1.1 equiv.), KO^{t}Bu (11.6 mg, 0.103 mmol, 1.1 equiv.) and THF (0.8 mL). The reaction mixture was stirred at room temperature for 24 hours, avoiding exposure to light (aluminium foil). The reaction was removed from the glove box and purification of the product was carried out by filtration through a syringe filter with THF (4 mL). Evaporation of the solvent, washing with diethyl ether (3x3 mL) and drying under high vacuum afforded the product as an off-white powder in 72% yield (45mg, 0.074 mmol).

^{1}H NMR (400 MHz, CDCl$_3$): δ (ppm) = 7.98 (d, *J* = 7.7 Hz, 2H, $H_{Ar\ cbz}$), 7.60 (t, *J* = 7.8 Hz, 2H, H_{Ar-IPr}), 7.39 (d, *J* = 7.8 Hz, 4H, H_{Ar-IPr}), 7.34 (d, J_{Ag-H} = 1.7 Hz, 2H, NCH_{Imid}), 7.07 (ddd, *J* = 8.2, 7.0, 1.3 Hz, 2H, $H_{Ar\ cbz}$), 6.93 – 6.87 (m, 2H, $H_{Ar\ cbz}$), 6.69 (d, *J* = 8.1 Hz, 2H, $H_{Ar\ cbz}$), 2.69 (hept, *J* = 6.9 Hz, 4H, C*H*(CH$_3$)$_2$), 1.34 (d, *J* = 6.9 Hz, 12H), 1.29 (d, *J* = 6.9 Hz, 12H, CH(C*H*$_3$)$_2$).

^{13}C {^{1}H} NMR (101 MHz, CDCl$_3$): δ (ppm) = 185.6 (dd, *J(Ag^{109}-C)* = 239.3 Hz, *J(Ag^{107}-C)* = 208.1 Hz, C-Ag), 150.4 (d, J_{Ag-C} = 4.7 Hz, C-N_{Cbz}), 146.0 (s, C-N_{Imid}), 134.9 (s, C_{Ar-IPr}), 130.9 (s, CH_{Ar-IPr}), 124.5 (s, CH_{Ar-IPr}), 124.0 (d, J_{Ag-C} = 5.0 Hz, C_{Ar-Cbz}), 123.7 (d, J_{Ag-C} = 6.8 Hz, NCH_{IPr}), 123.2 (s, CH_{Ar-Cbz}), 119.5 (s, CH_{Ar-Cbz}), 114.8 (s, CH_{Ar-Cbz}), 114.3 (s, CH_{Ar-Cbz}), 29.0 (s, *C*H(CH$_3$)$_2$), 24.9 (s, CH(*C*H$_3$)$_2$), 24.2 (s, CH(*C*H$_3$)$_2$).

^{1}H NMR (300 MHz, Acetone-d$_6$): δ (ppm) = 7.95 (d, J_{Ag-H} = 1.7 Hz, 2H), 7.84 (d, *J* = 7.7 Hz, 2H), 7.65 (t, *J* = 7.6 Hz, 2H), 7.50 (d, *J* = 7.6 Hz, 4H), 6.96 (ddd, *J* = 8.2, 7.0, 1.3 Hz, 2H), 6.76 (td, *J* = 7.4, 1.0 Hz, 2H), 6.69 – 6.64 (m, 2H), 2.80 (hept, *J* = 6.8 Hz, 4H), 1.36 (d, *J* = 6.9 Hz, 12H), 1.30 (d, *J* = 6.9 Hz, 12H).

Elemental analysis calcd (%) for $C_{39}H_{44}AgN_3$: C, 70.94; H, 6.69; N, 6.34; found: C 71.06; H 6.64; N 6.02.

Synthesis of [*N,N*-Bis(2,6-diisopropylphenyl)imidazolin-2-ylidene](9H-carbazol-9-yl)silver(I) [Ag(SIPr)Cbz] (5b):

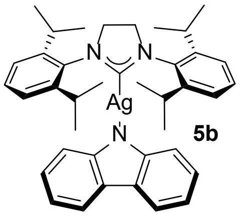

5b

Procedure (200 mg scale): A 20 mL scintillation vial equipped with a septum cap and a stirring bar was charged with [Ag(SIPr)Cl] (200 mg, 0.375 mmol,), carbazole (68.9. mg, 0.412 mmol,1.1 equiv.), K_2CO_3 (155 mg, 1.12 mmol, 3 equiv.) and acetone (2.8 mL). Full conversion had not been achieved in 20 hours of stirring at room temperature, as judged by NMR analysis of an aliquot (90% conversion), therefore the reaction was allowed to progress over 48 hours in total. After concentrating the reaction mixture to dryness, purification of the product was carried out by filtration through an alumina plug (1 cm) with THF (10 mL). Evaporation of the solvent under vacuum, washing with diethyl ether (3x5 mL) on a frit and drying under high vacuum afforded the product as an off-white, microcrystalline solid in 93% yield (232 mg, 0.349 mmol).

^{1}H NMR (400 MHz, CDCl$_3$): δ (ppm) = 7.94 (dd, *J* = 7.2, 0.5 Hz, 2H, $H_{Ar\ cbz}$), 7.54 (t, *J* = 7.8 Hz, 2H $H_{Ar-SIPr}$), 7.36 (d, *J* = 7.8 Hz, 4H, $H_{Ar-SIPr}$), 7.02 (ddd, *J* = 8.2, 7.0, 1.3 Hz, 2H, $H_{Ar\ cbz}$), 6.87 (td, *J* = 7.4, 1.0 Hz, 2H, $H_{Ar\ cbz}$), 6.48 (d, *J* = 8.1 Hz, 2H, $H_{Ar\ cbz}$), 4.21 (s, 4H,NCH$_2$), 3.20 (hept, *J* = 6.8 Hz, 4H, C*H*(CH$_3$)$_2$), 1.40 (d, *J* = 7.3 Hz, 12H, CH(C*H*$_3$)$_2$), 1.38 (d, *J* = 7.2 Hz, 12H, CH(C*H*$_3$)$_2$).

^{13}C {^{1}H} NMR (101 MHz, CDCl$_3$): δ (ppm) = 208.9 (dd, *J(Ag^{109}-C)* = 225 Hz, *J(Ag^{107}-C)* = 195 Hz, C-Ag), 150.4 (d, J_{Ag-C} = 4.6 Hz, C-N_{Cbz}), 147.1 (s, C-N_{Imid}), 134.9 (s, $C_{Ar-Imid}$), 130.2 (s, $CH_{Ar-Imid}$), 124.9 (s, $CH_{Ar-Imid}$), 123.9 (d, J_{Ag-C} = 5.0 Hz, C_{Ar-Cbz}), 123.2 (s, CH_{Ar-cbz}), 119.44(s, CH_{Ar-cbz}), 114.8 (s, CH_{Ar-cbz}), 114.3 (s, CH_{Ar-cbz}), 54.0 (d, J_{Ag-C} = 7.8 Hz, NCH_{Imid}), 29.2 (s, *C*H(CH$_3$)$_2$), 25.5 (s, CH(*C*H$_3$)$_2$), 24.2 (s, CH(*C*H$_3$)$_2$).

^{1}H NMR (400 MHz, Acetone-d$_6$): δ (ppm) = 7.83 – 7.80 (m, 2H), 7.61 – 7.55 (t, *J* = 7.7 Hz, 2H), 7.45 (d, *J* = 7.7 Hz, 4H), 6.91 (ddd, *J* = 8.2, 7.0, 1.3 Hz, 2H), 6.74 (ddd, *J* = 7.8, 7.0, 1.0 Hz, 2H), 4.43 (s, 4H), 3.42 (hept, *J* = 6.9 Hz, 4H), 1.40 (d, *J* = 0.7 Hz, 12H), 1.39 (d, *J* = 0.8 Hz, 12H).

Elemental analysis calcd (%) for $C_{39}H_{46}AgN_3$: C, 70.47; H, 6.98; N, 6.32; found: C 70.34; H 7.11; N 6.30.

Synthesis of [*N,N*-Bis(2,6-bis(diphenylmethyl)-4-methylphenyl)imidazol-2-ylidene](9H-carbazol-9-yl)silver(I) [Ag(IPr*)Cbz] (5c):

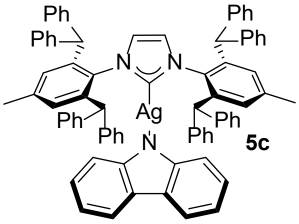

5c

Procedure (200 mg scale): A 20 mL scintillation vial equipped with a septum cap and a stirring bar was charged with [Ag(IPr*)Cl] (200 mg, 0.189 mmol,), carbazole (34.8. mg, 0.208 mmol,1.1 equiv.), K_2CO_3 (92 mg, 0.66 mmol, 3.5 equiv.) and acetone (2.8 mL). Full conversion had not been achieved in 20 hours of stirring at room temperature, as judged by NMR analysis of an aliquot (90% conversion), therefore the reaction was allowed to progress over 48 hours in total. After concentrating the reaction mixture to dryness,

purification of the product was carried out by filtration through a syringe filter and then an alumina plug (1 cm) with THF (10 mL). Evaporation of the solvent under vacuum, washing sequentially with pentane (3x5 mL) and diethyl ether (3x5 mL) and then drying under high vacuum afforded the product as a white solid in 89% yield (200 mg, 0.168 mmol).

^{1}H NMR (400 MHz, CD_2Cl_2): δ (ppm) = 8.12 – 8.02 (m, 2H, $H_{Ar\ cbz}$), 7.28 – 7.19 (m, 12H, H_{Ar}), 7.04 (ddd, J = 8.1, 6.9, 1.3 Hz, 2H, $H_{Ar\ cbz}$), 7.00 – 6.91 (m, 24H, H_{Ar}), 6.86 – 6.78 (m, 10H, H_{Ar}), 6.05 (d, $J_{Ag\text{-}H}$ = 1.7 Hz, 2H, NCH_{Imid}), 5.31 (s, 4H, $CHPh_2$), 2.32 (s, 6H, CH_3).

^{13}C NMR (101 MHz, CD_2Cl_2): δ (ppm) = 185.3 (dd, $J(Ag^{109}\text{-}C)$ = 237.4 Hz, $J(Ag^{107}\text{-}C)$ = 207.1 Hz, C-Ag), 151.1 (d, $J_{Ag\text{-}C}$ = 4.5 Hz, C-N_{Cbz}), 143.6 (s, C_{Ar}), 142.9 (s, C_{Ar}), 141.8 (s, C_{Ar}), 140.9 (s, C_{Ar}), 135.2 (s, C-N_{Imid}), 130.9 (s, CH_{Ar}), 129.9 (s, CH_{Ar}), 129.9 (s, CH_{Ar}), 129.1 (s, CH_{Ar}), 129.0 (s, CH_{Ar}), 127.4 (s, CH_{Ar}), 127.1 (s, CH_{Ar}), 124.6 (d, $J_{Ag\text{-}C}$ = 5.0 Hz, $C_{Ar\text{-}Cbz}$) 124.4 (d, $J_{Ag\text{-}C}$ = 6.5 Hz, NCH_{Imid}) 123.7 (s, CH_{Ar}), 119.6 (s, CH_{Ar}), 115.3 (s, CH_{Ar}), 115.2 (s, CH_{Ar}), 51.97 (s,$CHPh_2$), 22.10 (s, CH_3).

Elemental analysis calcd (%) for $C_{81}H_{64}AgN_3$: C, 81.94; H, 5.47; N, 3.54; found: C, 81.82; H 5.33; N 3.39.

Synthesis of [*N,N*-Bis(2,6-diisopropylphenyl)imidazol-2-ylidene](9H-carbazol-9-yl)copper(I) [Cu(IPr)Cbz] (6a):

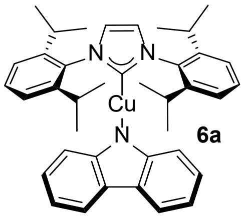

6a

Procedure A (small scale): A 4 mL scintillation vial equipped with a septum cap and a stirring bar was charged with [Cu(IPr)Cl] (39.3 mg, 0.081 mmol), carbazole (14.1 mg, 0.085 mmol, 1.05 equiv.), K_2CO_3 (33.4 mg, 0.242 mmol, 3 equiv.) and ethanol (0.5 mL). The reaction mixture was stirred at room temperature for 24 hours. The solvent was removed under vacuum and purification of the product was carried out by filtration through a syringe membrane filter with THF (4 mL). Evaporation of the solvent, washing with diethyl ether (3x3 mL) and drying under high vacuum afforded the product as a white powder in 87% yield (43 mg, 0.070 mmol).

Procedure A (large scale): A 250 mL round bottom flask equipped with a septum cap and a heavy stirring bar was charged with [Cu(IPr)Cl] (5.00 g, 10.25 mmol,), carbazole (96%, 1.806 g, 10.25 mmol,1.00 equiv.), K_2CO_3 (5.67 g, 41.0 mmol, 4 equiv.) and ethanol (60 mL). Full conversion had been achieved in 24 hours of stirring at room temperature, as judged by NMR analysis of an aliquot. After concentrating the reaction mixture to dryness, purification of the product was carried out by filtration through a 3 cm pad of celite, covered by a 1 cm layer of basic alumina with THF (700 mL). Evaporation of the solvent under vacuum, washing sequentially with pentane (2x50 mL), diethyl ether (3x30 mL) and pentane (2x50 mL) on a frit and drying under high vacuum afforded the product as a white, microcrystalline solid in 95% yield (6.04 g, 9.77 mmol).

Procedure B (one pot): A vial was charged, under air, with 100 mg of IPr•HCl (0.235 mmol), 65 mg of K_2CO_3 (0.471 mmol, 2 equiv.), 23.3 mg of CuCl (0.235 mmol, 1 equiv.), and the solids were suspended in acetone (0.5 mL). The reaction mixture was stirred at 60ºC for 24 hours. Then, the reaction was cooled to room temperature and 43.3 mg of carbazole (0.259 mmol, 1.1 equiv.), 113.8 mg of K_2CO_3 (0.824 mmol, 3.5 equiv.) and 0.5 mL of acetone were added. The reaction mixture was stirred at room temperature for 22h. After this time the solvent was removed under vacuum and the residue was taken up in THF (2 mL) and filtered through basic alumina which was washed using THF (2 mL). The filtrate was concentrated to dryness, and the resulting solid was washed with diethyl ether and dried, affording the desired complex as a white solid in 79% yield (103 mg, 0.186 mmol).

Procedure C (mechanosynthesis): A 12 mL ZrO_2 reactor equipped with 18 milling balls (Ø = 5 mm) was charged in air with [Cu(IPr)Cl] (100.0 mg, 0.21 mmol), carbazole (44.6 mg, 0.27 mmol, 1.3 equiv.), and K_2CO_3 (113.4 mg, 0.82 mmol, 4 equiv.). The mixture of solids was ground in a planetary ball mill at 400 rpm for 30 min. At the end of the 30 min, the content of the jar was extracted in THF (2x3 mL) and the solution microfiltered. After removal of the solvent by rotatory evaporation, the solid residue was washed with diethyl ether (2x2 mL). Following removal of the supernatant, the solid was further triturated using 2 mL of pentane and then dried under high vacuum, affording the product as a white, microcrystalline solid in 81% yield (102.8 mg, 0.17 mmol).

^{1}H NMR (400 MHz, CD_2Cl_2): δ (ppm) = 7.87 – 7.82 (m, 2H), 7.70 (t, J = 7.8 Hz, 2H), 7.48 (d, J = 7.8 Hz, 4H), 7.34 (s, 2H), 6.95 (ddd, J = 8.2, 7.0, 1.3 Hz, 2H), 6.85 – 6.80 (m, 2H), 6.29 (d, J = 8.1 Hz, 2H), 2.74 (hept, J = 6.8 Hz, 4H), 1.30 (overlapping d, J = 6.8 Hz, 24H).

^{1}H NMR (300 MHz, Acetone-d_6): δ (ppm) = 7.87 (s, 2H), 7.80 (ddd, J = 7.6, 1.2, 0.7 Hz, 2H), 7.76 – 7.70 (m, 2H), 7.55 (d, J = 7.8 Hz,4H), 6.87 (ddd, J = 8.2, 7.0, 1.3 Hz, 2H), 6.75 (td, J = 7.4, 1.0 Hz, 2H), 6.34 (dt, J = 8.2, 0.8 Hz, 2H), 2.89 – 2.75 (m, 4H), 1.30 (overlapping d, J = 6.8 Hz, 24H).

^{1}H NMR (300 MHz, $CDCl_3$): δ (ppm) = 7.92 (dd, J = 7.6, 0.6 Hz, 2H), 7.72 – 7.63 (m, 2H), 7.45 (d, J = 7.8 Hz, 4H), 7.29 (s, 2H), 6.97 (ddd, J = 8.2, 7.0, 1.3 Hz, 2H), 6.91 – 6.83 (m, 2H), 6.32 (d, J = 8.0 Hz, 2H), 2.79 – 2.63 (m, 4H), 1.29 (overlapping d, J = 7.0 Hz, 24H).

^{13}C NMR (75 MHz, $CDCl_3$): δ (ppm) = 182.4, 150.1, 146.3, 134.8, 130.7, 124.5, 124.0, 123.2, 119.4, 115.1, 114.4, 29.1, 24.9, 24.1.

Analytical data obtained are in agreement with reported values.[16]

Synthesis of [*N,N*-Bis(mesityl)imidazol-2-ylidene](9H-carbazol-9-yl)copper(I) [Cu(IMes)Cbz] (6b):

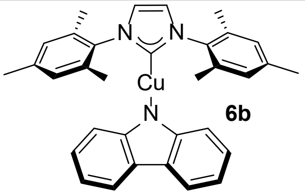

6b

A 4 mL scintillation vial equipped with a septum cap and a stirring bar was charged with [Cu(IMes)Cl] (80.9 mg, 0.2 mmol), carbazole (36.8 mg, 0.22 mmol, 1.1 equiv.), K_2CO_3 (96.7 mg, 0.7 mmol, 3.5 equiv.) and acetone (0.8 mL). The reaction mixture was stirred at 60 ^{o}C for 48 hours. The solvent was removed under vacuum and purification of the product was carried out by filtration through a basic alumina plug (1 cm) with THF (2 mL). Evaporation of the solvent, washing with diethyl ether (3x3 mL) and drying under high vacuum afforded the product as a white powder in 75% yield (80 mg, 0.070 mmol).

^{1}H NMR (400 MHz, CD_2Cl_2): δ (ppm) = 7.86 (d, J = 7.6 Hz, 2H), 7.25 (s, 2H), 7.18 (s, 4H), 7.00 (t, J = 7.4 Hz, 2H), 6.84 (t, J = 7.3 Hz, 2H), 6.51 (d, J = 8.0 Hz, 6H), 2.47 (s, 3H), 2.23 (s, 12H).

^{13}C NMR (75 MHz, CD_2Cl_2): δ (ppm) = 180.2 (seen in HMBC),150.5, 140.4, 136.1, 135.8, 129.9, 124.3, 123.8, 122.9, 119.7, 115.7, 114.9, 21.5, 18.3.

Analytical data obtained are in agreement with reported values. [16]

Synthesis of (2-(2,6-diisopropylphenyl)-3,3-dimethyl-2-azaspiro[4.5]decan-1-yl)(9H-carbazol-9-yl)copper(I) [Cu(CAAC^{Cy})(Cbz)] (6c):

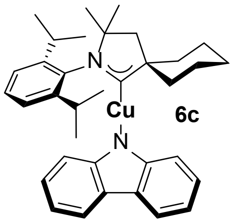

6c

A 4 mL scintillation vial equipped with a septum cap and a stirring bar was charged with [Cu(CAAC^{Cy})Cl] (45.5 mg, 0.1 mmol), carbazole (17.6 mg, 0.105 mmol, 1.05 equiv.), K_2CO_3 (41.5 mg, 0.3 mmol, 3.0 equiv.) and ethanol (0.5 mL). The reaction mixture was stirred at 40 ^{o}C for 20 hours. The solvent was removed under vacuum and purification of the product was carried out by filtration through a basic alumina plug (1 cm) with THF (2 mL). Evaporation of the solvent, washing with diethyl ether (3x3 mL) and drying under high vacuum afforded the product as a white powder in 79% yield (44 mg, 0.079 mmol).

^{1}H NMR (300 MHz, CD_2Cl_2): δ (ppm) = 7.91 (ddd, J = 7.7, 1.2, 0.7 Hz, 2H, $H_{Ar\ cbz}$), 7.66 (t, J = 7.8 Hz, 1H, H_{Ar}), 7.46 (d, J = 7.8 Hz, 2H, H_{Ar}), 7.07 (ddd, J = 8.2, 7.0, 1.3 Hz, 2H, $H_{Ar\ cbz}$), 6.89 (ddd, J = 7.8, 7.0, 1.0 Hz, 2H, $H_{Ar\ cbz}$), 6.70 – 6.64 (m, 2H, $H_{Ar\ cbz}$), 2.99 (hept, J = 6.8 Hz, 2H, C*H*(CH_3)$_2$), 2.35 (td, J = 12.8, 3.6 Hz, 2H, C$H_{2\text{-Cy}}$), 2.20 (s, 2H, C$H_{2\text{-CAAC}}$), 2.11 – 2.01 (m, 2H, C$H_{2\text{-CAAC}}$), 1.86 (m, 1H, C$H_{2\text{-CAAC}}$) 1.64 (m, 5H, C$H_{2\text{-CAAC}}$), 1.46 (s, 6H, CH_3), 1.35 (d, J = 6.8 Hz, 6H, CH(C*H*$_3$)$_2$), 1.22 (d, J = 6.7 Hz, 6H, CH(C*H*$_3$)$_2$).

^{13}C NMR (75 MHz, CD_2Cl_2): δ (ppm) = 251.2 (C_{CAAC}-Cu), 150.5 (C-N_{Cbz})r, 146.4 (C_{Ar}-N_{CAAC}), 135.9 ($C_{Ar\text{-}CAAC}$), 130.3 ($CH_{Ar\text{-}CAAC}$), 125.7 ($CH_{Ar\text{-}CAAC}$), 124.7 (C_{Ar}), 123.8 ($CH_{Ar\text{-}Cbz}$), 119.7 ($CH_{Ar\text{-}Cbz}$), 115.9 ($CH_{Ar\text{-}Cbz}$), 115.1 ($CH_{Ar\text{-}Cbz}$), 81.0 (C-N_{CAAC}), 60.0 ($C_{Cy\text{-}CAAC}$), 47.0 ($CH_{2\text{-}CAAC}$), 37.2 ($CH_{2\text{-}CAAC}$), 30.1 (CH_3), 29.8(*C*H(CH_3)$_2$), 27.0 (CH(*C*H$_3$)$_2$), 26.3 ($CH_{2\text{-}CAAC}$), 23.0 (CH(*C*H$_3$)$_2$), 22.9($CH_{2\text{-}CAAC}$).

Elemental analysis calcd (%) for $C_{35}H_{43}CuN_2$: C, 75.71; H, 7.81; N, 5.04; found: C: 75.12%, H: 5.60%, N: 5.06%

Scope of amines

Synthesis of [*N,N*-Bis(2,6-diisopropylphenyl)imidazol-2-ylidene](pyridin-2-ylamino)gold(I) [Au(IPr)(2-AmPy)] (4h):

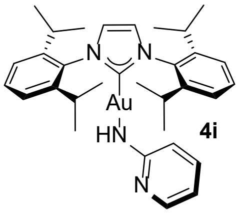

4i

A vial was charged, under air, with 53.8 mg of [Au(IPr)Cl] (0.086 mmol), 36.1 mg of K_2CO_3 (0.261 mmol, 3 equiv.), 8.7 mg of 2-aminopyridine (0.093 mmol, 1.1 equiv.) and the solids were suspended in technical grade acetone (0.5 mL). The reaction mixture was stirred at room temperature for 24 hours. After this time the solvent was removed in vacuo and dichloromethane was added (1 mL). The mixture was filtered on a syringe membrane filter and addition of diethylether (1 mL) to the concentrated solution yielded the final complex as a white solid which was filtered and dried under vacuum (85%, 50 mg, 0.073 mmol).

^{1}H NMR (400 MHz, CD_2Cl_2): δ (ppm) = 7.55 (t, *J* = 7.8 Hz, 2H; m, 1H), 7.35 (d, *J* = 7.8 Hz, 4H), 7.23 (s, 2H), 6.75 (t, *J* = 8.5 Hz, 1H), 5.94 (m, 1H), 5.59 (d, *J* = 8.5 Hz, 1H), 4.21 (s, 1H), 2.59 (hept, *J* = 7.0 Hz, 4H), 1.34 (d, *J* = 7.0 Hz, 12H), 1.24 (d, *J* = 7.0 Hz, 12H).

^{13}C NMR (101 MHz, CD_2Cl_2): δ (ppm) = 179.6, 169.0 , 147.9, 146.2, 135.8, 135.0, 130.7, 124.6, 123.7, 109.9, 107.6, 29.2, 24.6, 24.2.

Analytical data obtained are in agreement with reported values.[17]

Synthesis of [*N,N*-Bis(2,6-diisopropylphenyl)imidazol-2-ylidene](isoquinolin-3-ylamino)gold(I) [Au(IPr)(3-AmIsoquin)] (4i):

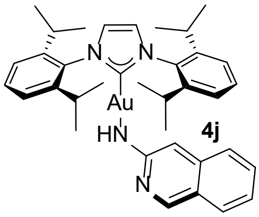

4j

A vial was charged, under air, with 60.9 mg of [Au(IPr)Cl](0.098 mmol), 40.7 mg of K_2CO_3 (0.294 mmol, 3 equiv.), 15.6 mg of 3-aminoisoquinoline(0.098 mmol, 1.1 equiv.) and the solids were suspended in technical grade acetone (0.5 mL). The reaction mixture was stirred at room temperature for 24 hours. After this time the solvent was removed in vacuo and dichloromethane was added (1 mL). The mixture was filtered on a syringe membrane filter and addition of pentane (2 mL) to the concentrated solution yielded the final complex as a white solid which was filtered and dried under vacuum (83%, 59 mg, 0.081 mmol).

^{1}H NMR (400 MHz, CD_2Cl_2): δ (ppm) = 8.34 (s, 1H), 7.62 (t, *J* = 7.8 Hz, 2H), 7.41 (d, J = 7.8 Hz, 4H), 7.37 (m, 1H), 7.25 (s, 2H), 7.17 (ddd, *J* = 8.2, 6.7, 1.3 Hz, 1H), 6.95 (d, *J* = 8.5 Hz, 1H), 6.75 (ddd, *J* = 8.01, 6.71, 1.12 Hz, 1H), 5.80 (m, 1H), 4.33 (s, 1H), 2.67 (hept, *J* = 6.9 Hz, 4H), 1.37 (d, *J* = 6.9 Hz, 12H), 1.25 (d, *J* = 6.9 Hz, 12H).

^{13}C NMR (101 MHz, CD_2Cl_2): δ (ppm) = 179.9, 166.2, 151.3, 146.5, 140.0, 134.9, 130.9, 129.2, 128.1, 124.6, 123.8, 123.5, 121.4, 118.7, 96.5, 29.3, 24.5, 24.2.

Analytical data obtained are in agreement with reported values.[17]

Synthesis of [*N,N*-Bis(2,6-diisopropylphenyl)imidazol-2-ylidene](diphenylamido)gold(I) [Au(IPr)(NPh_2)] (4j):

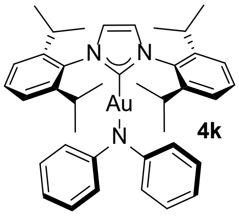

4k

Procedure A (small scale): A vial was charged, under air, with 51.6 mg of [Au(IPr)Cl] (0.083 mmol), 57.8 mg of K_2CO_3 (0.249 mmol, 3 equiv.), 15.6 mg of diphenylamine (0.091 mmol, 1.1 equiv.) and the solids were suspended in ethanol (1.0 mL). The reaction mixture was stirred at 40°C for 24 hours. After this time the solvent was removed in vacuo and dichloromethane was added (2 mL). The mixture was filtered on a syringe membrane filter and addition of pentane (2 mL) to the concentrated solution yields the final complex as a pale yellow solid which was filtered and dried under vacuum (90%, 56.2 mg, 0.075 mmol).

Procedure A (large scale): A round-bottom flask equipped with a stirring bar and a septum was charged, under air, with 0.937 g of [Au(IPr)Cl] (1.51 mmol), 0.6248 g of K_2CO_3 (4.52 mmol, 3 equiv.), 0.281 g of diphenylamine (1.659 mmol, 1.1 equiv.) and the solids were suspended in ethanol (20 mL). The reaction mixture was stirred at 40°C for 24 hours. After this time the solvent was removed in vacuo and dichloromethane was added (20 mL). The mixture was filtered on a celite pad and addition of pentane (10 mL) to the concentrated solution yields the final complex as a pale yellow solid which was filtered and dried under vacuum (94%, 1.069 g, 1.42 mmol).

^{1}H NMR (400 MHz, CD_2Cl_2): δ (ppm) = 7.60 (t, *J* = 7.8 Hz, 2H), 7.36 (d, *J* = 7.8 Hz, 4H,), 7.27 (s, 2H), 6.79-6.74 (m, 4H), 6.64-6.61 (m, 4H), 6.41 (tt, *J* = 7.1, 1.1 Hz, 2H), 2.61 (hept, *J* = 6.9 Hz, 4H), 1.26 (d, *J* = 6.9 Hz, 12H), 1.22 (d, *J* = 6.9 Hz, 12H).

^{13}C NMR (101 MHz, CD_2Cl_2): δ (ppm) = 178.4, 154.7, 146.4, 135.0, 130.7, 128.5, 124.5, 123.4, 119.7, 116.6, 29.2, 24.4, 24.2.

Analytical data obtained are in agreement with reported values. [17]

Results and Discussion

Weak base tests and DFT calculations

In order to probe the limits with regards to the mild bases that can be used to carry out such synthetic procedures, we used the weak bases shown in table S1 under the standard operational conditions (Scheme S1).

Scheme S1. Standard operational conditions for the base tests on the N-H metalation of carbazole.

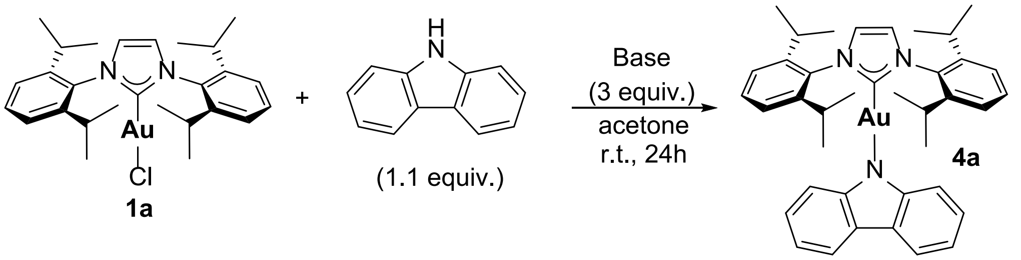

Interestingly, only K_2CO_3 led to full conversion, while triethylamine and $KHCO_3$ led to low conversions and NaOAc was not able to facilitate the reaction. This supports the hypothesis that K_2CO_3 acts as a monobasic reagent. Furthermore, the kinetic barriers calculated for other bases are all higher, while the reactions were either disfavoured (in case of NEt_3 which was approximated by NMe_3 for computational ease) or only slightly favored (Figure S1). Of note, when evaluating kinetic barriers, it should be taken into account that these reactions are carried out at room temperature and are suspensions, therefore the active concentrations of the metal species in solution are low. K_2CO_3 proved superior kinetically and thermodynamically (*vide infra*).

Table S1. Weak base effect on the N-H metallation of carbazole.

Entry[a]	Base	Conversion (%)[b]
1	K_2CO_3	100
2	NEt_3	24
3	NaOAc	0
4	$KHCO_3$	17

[a] Performed on a 0.081 mmol scale with 0.5 mL of acetone. [b] Determined by NMR.

Intrigued by the fact that 2-aminopyridine was also an amenable substrate despite having a drastically higher pKa value, we investigated the performance of other weak bases in the synthesis of **4i**. Only K_2CO_3 successfully led to the desired product, while no conversion was achieved with any of the other bases. The kinetic barrier in the case of using K_2CO_3 for the auration of this amine was significantly higher than that in the case of carbazole, however still in an achievable range and the reaction is thermodynamically favoured (Figure S2).

Scheme S2. Standard operational conditions for the base tests on the N-H metalation of 2-aminopyridine.

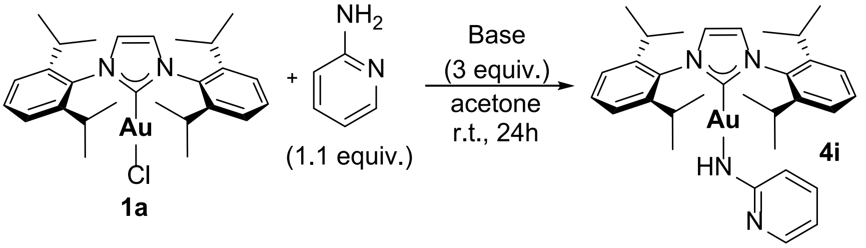

Table S2. Weak base effect on the N-H metallation of 2-aminopyridine.

Entry[a]	Base	Conversion (%)[b]
1	K_2CO3	100
2	NEt_3	0
3	NaOAc	0
4	$KHCO_3$	0

[a] Performed on a 0.081 mmol scale with 0.5 mL of acetone. [b] Determined by NMR.

The experimental results are in agreement with the theoretical calculations detailed below. Additionally, the N-H metallation of carbazole with silver and copper using K_2CO_3, was also studied computationally, suggesting that the transformation is favoured thermodynamically and kinetic barriers are low for all coinage metals (Figure S3).

Figure S1. Free energy profiles (kcal/mol, acetone) for the synthesis of carbene-Au-amido complexes in presence of the four weak bases considered.

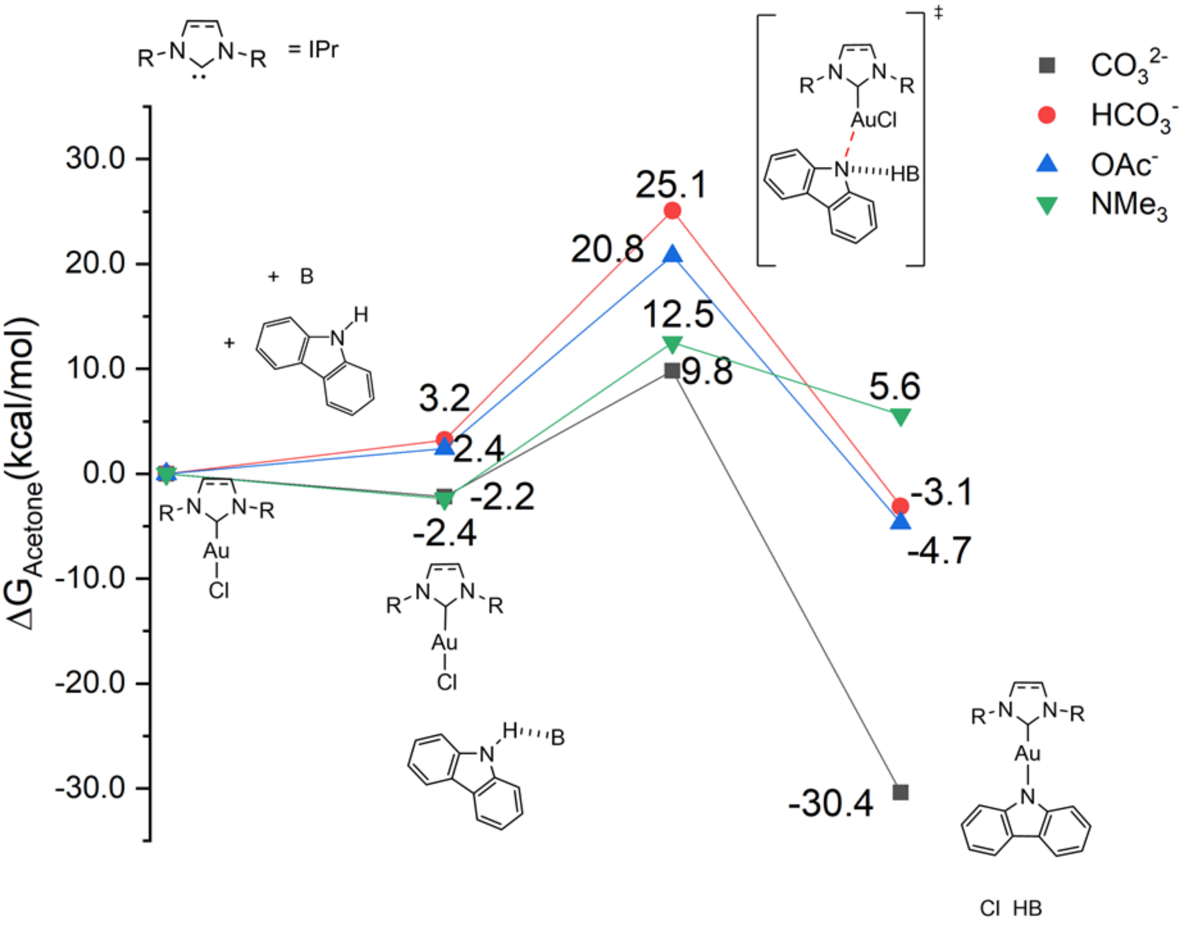

Figure S2. Free energy profile (kcal/mol, acetone) for the synthesis of carbene-Au-amido complex in presence of of CO_3^{2-} weak base.

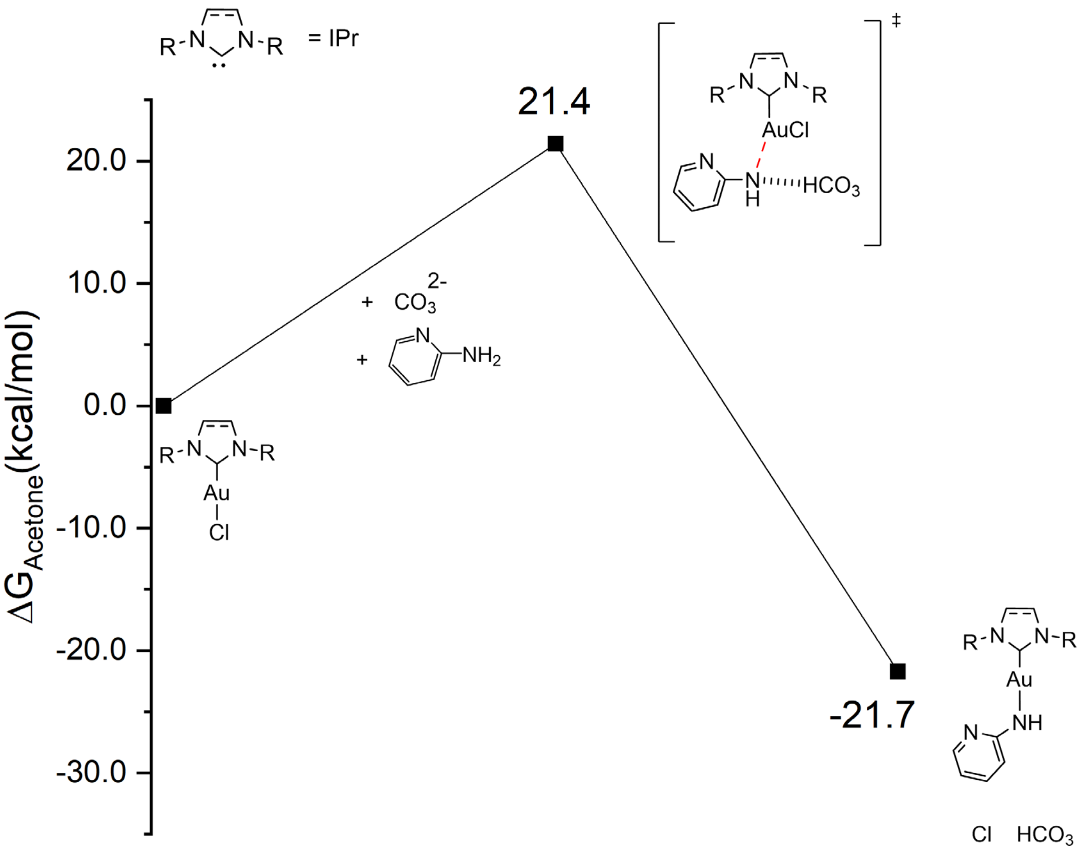

Figure S3. Free energy profiles (kcal/mol, acetone) for synthesis of carbene-M-amido (M=Ag, Cu) complexes in presence of CO_3^{2-} weak base.

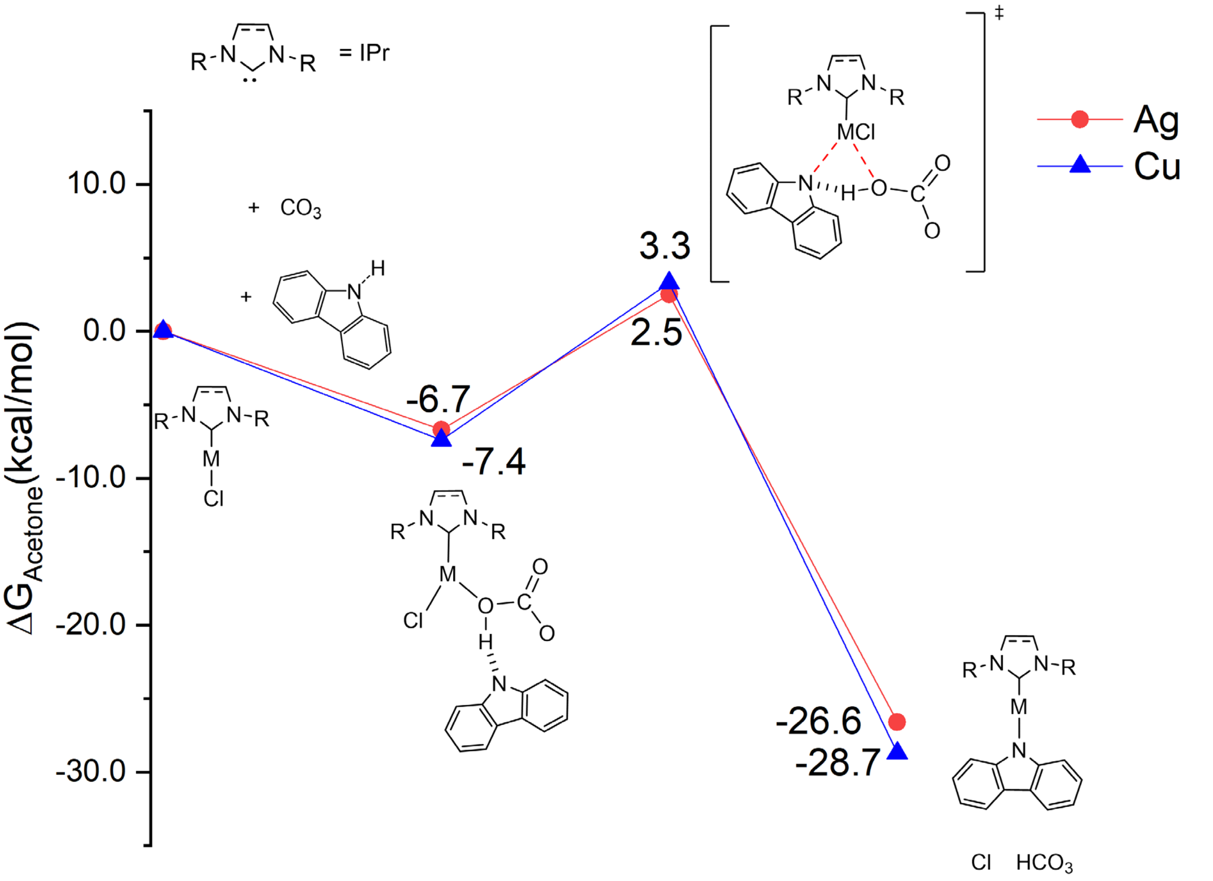

Molecular structures of complexes 4b, 4e, 4f, 5a, 5c, 6a and 6c

Crystals that were of suitable quality for single crystal X-ray diffraction analysis were obtained in all cases by slow vapor diffusion of the antisolvent (pentane) into saturated solutions of the complexes (in acetone or dichloromethane) at 4 ºC. CCDC 2054515-2054517 and 2054519-2054522 (**4b**, **4e**, **4f**, **5a**, **5c**, **6a** and **6c**) contain the supplementary crystallographic data for this paper. These data can be obtained free of charge from The Cambridge Crystallographic Data Centre via www.ccdc.cam.ac.uk/structures.

FigureS4. X-ray molecular structures of complexes **4b**, **4e**, **4f**, **5a**, **5c**, **6a** and **6c** are presented, showing thermal displacement ellipsoids at the 50% probability level and hydrogen atoms omitted for clarity.

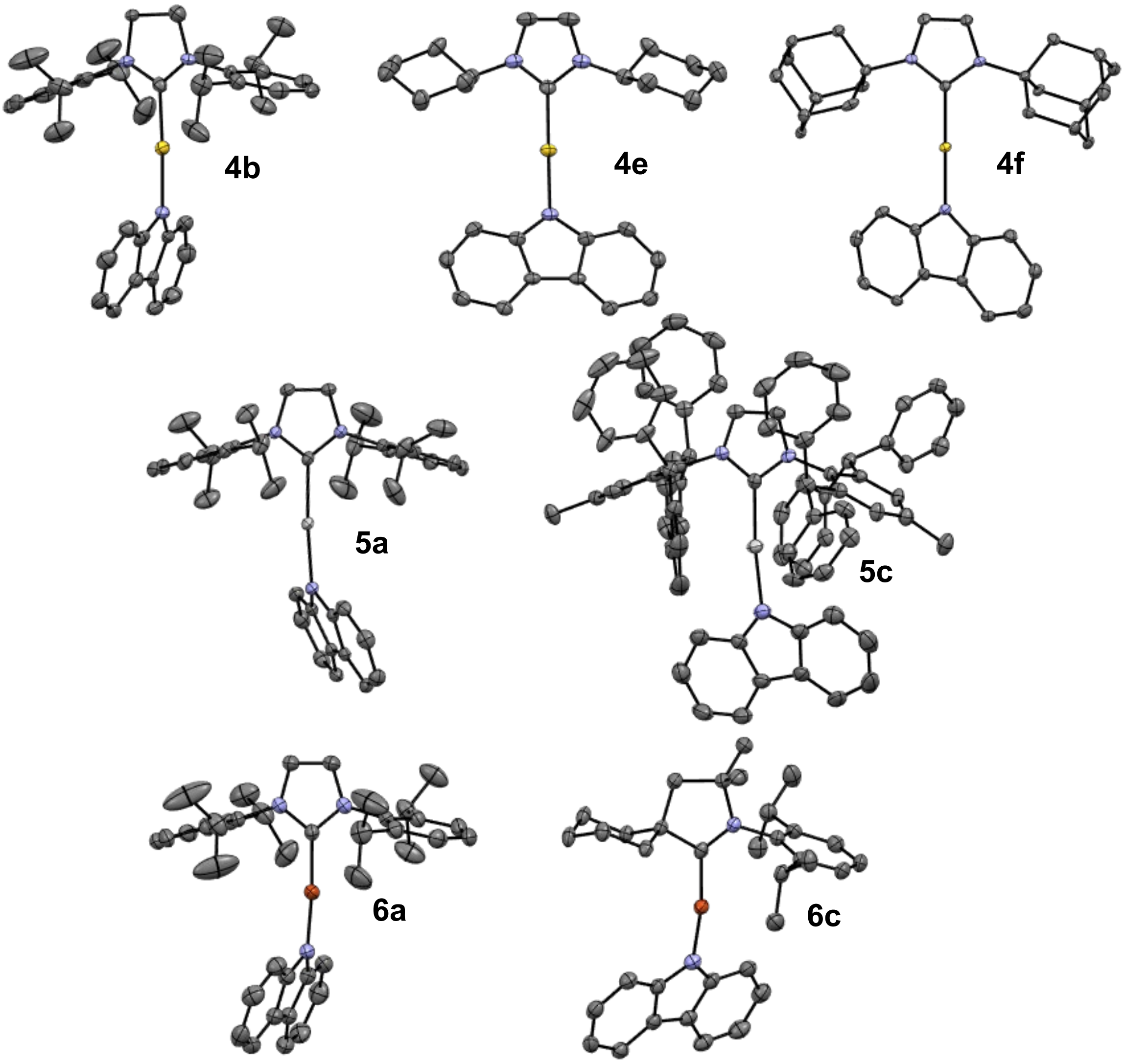

Selected structural data for these compounds may be found in table S3. The highest values for M-N and M-C bond lengths are observed in the case of the silver complexes **5a** and **5c**, while the maximum deviation from linear geometry is observed in the case of complex **6c**.

Table S3. Selected bond lengths (Å) and angles (°) for each complex

Complex	C_{NHC}-M	M-N	C_{NHC}-M-N
**4b**	1.990(7)	2.022(60	177.5(3)
**4e**	1.983	2.026	176.9
**4f**	1.998(4)	2.026(3)	179.8(1)

**5a**	2.056(2)	2.058(1)	175.42(6)
**5c**	2.056(4)	2.068(3)	174.0(1)
**6a**	1.882(3)	1.873(3)	175.4(1)
**6c**	1.874(4)	1.860(3)	171.5(1)

Photophysical properties of 4a,b and 5a,b and analytical data for photocatalysis

Emission scan series for the determination of the stability of the investigated complexes were performed by running multiple (5-8) emission scans under identical conditions consecutively and without additional delay between the scans, guaranteeing permanent irradiation of the sample at λ_{ex}. We encountered stability issues in THF, particularly at low concentrations (10^{-6} M to low 10^{-5} M), indicating minor (photo-)decomposition, which could be due to traces of moisture or oxygen (low ppm range) that are left in the solvent even after meticulous drying and degassing. At higher concentrations, typically used for NMR spectroscopy and photocatalysis, this effect is not observable. Furthermore, when examining the absorption data for the determination of extinction coefficients, extrapolation of just the data at very low concentrations would give a steeper slope and, therefore, an overestimation of ε for the same reason (see Figure S8).

Depictions of the transition density differences of the two lowest excited singlet and triplet states are given in Figure S14.

An overview of the ^{1}H NMR data for the determination of conversion in the photocatalytic [2+2] cycloaddition reactions with **4b** as a catalyst are shown in Figure S16.

Table S4. Selected photophysical data for **4a,b** and **5a,b** in THF solution and in the solid state at room temperature.

Cpd	THF solution				solid state	
	λ_{abs} [nm] (ε [10^3 $M^{-1}cm^{-1}$])	λ_{em}[a] [nm]	τ_{av} [µs]	Φ	λ_{em}[a] [nm]	τ_{av} [µs]
**4a**	239 (37.4), 278 (23.1), 308 (13.8), 332 (11.2), 350 (3.9), 369 (2.5)	431	335	0.33	424<br>550	74<br>885
**4b**	239 (36.0), 278 (18.0), 308 (11.2), 332 (10.2), 350 (6.3), 369 (2.7)	429	266	0.32	424<br>550	38<br>374
**5a**	239 (36.5), 278 (27.8), 311 (12.6), 328 (3.5), 357 (2.6), 376 (3.0)	343<br>437	0.014<br>10^{0}[b]	0.08	393<br>510	10^{-3}[b]<br>45
**5b**	239 (43.7), 278 (28.7), 311 (12.1), 328 (4.7), 357 (3.0), 376 (3.3)	359<br>438	0.014<br>332	0.44	396<br>509	0.002<br>335

[a] Detection wavelength for lifetime measurements. [b] Due to low emission intensity and stability issues, only the order of magnitude is reported.

PL spectra of [Au(IPr)Cbz] (4a):

Figure S5. Absorption, excitation and emission scan series in THF solution.

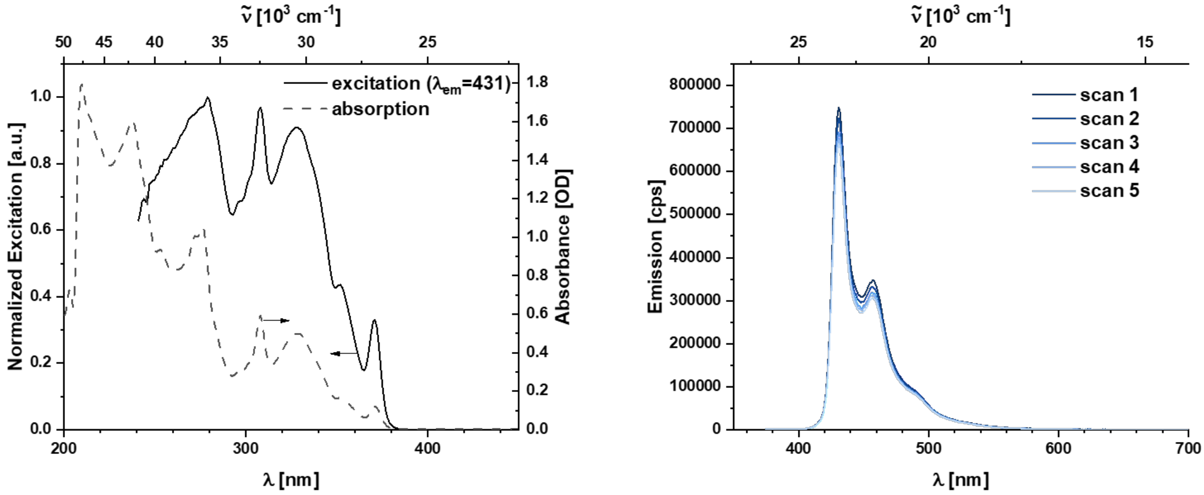

Figure S6. Excitation, emission and emission scan series in the solid state.

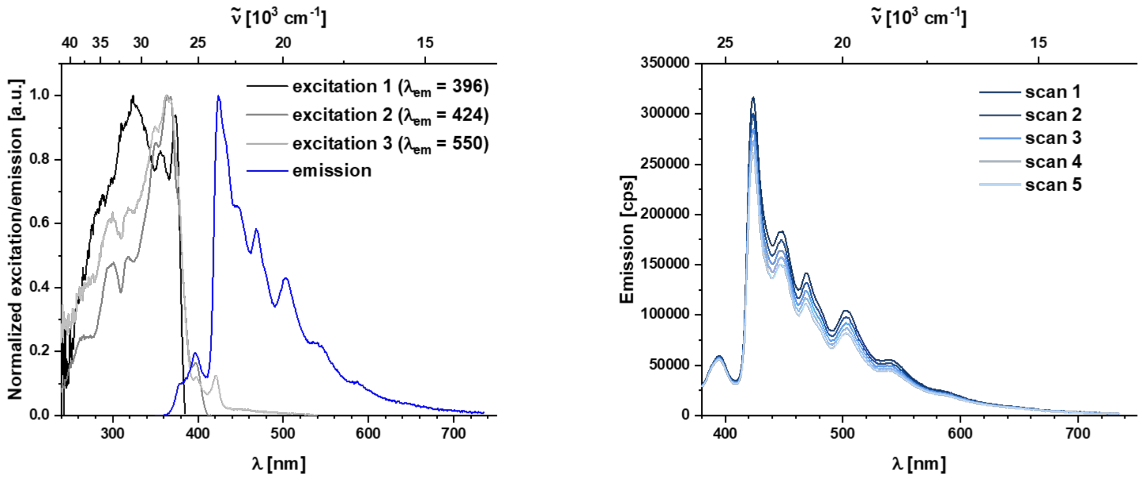

PL spectra of [Au(SIPr)Cbz] (4b):

Figure S7. Absorption, excitation and emission scan series in thf solution.

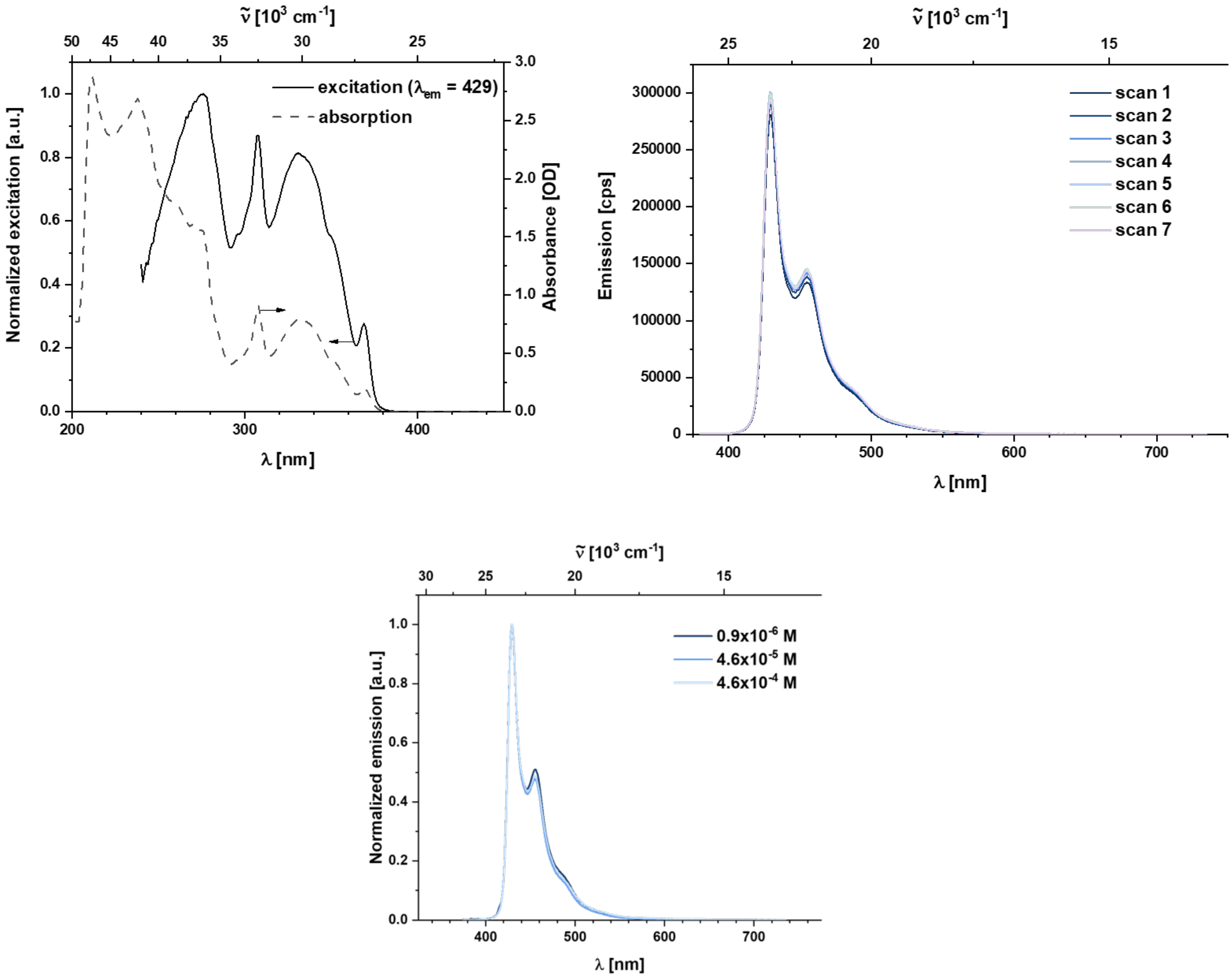

Figure S8. Extrapolation of various extinction coefficients from absorption data at different concentrations.

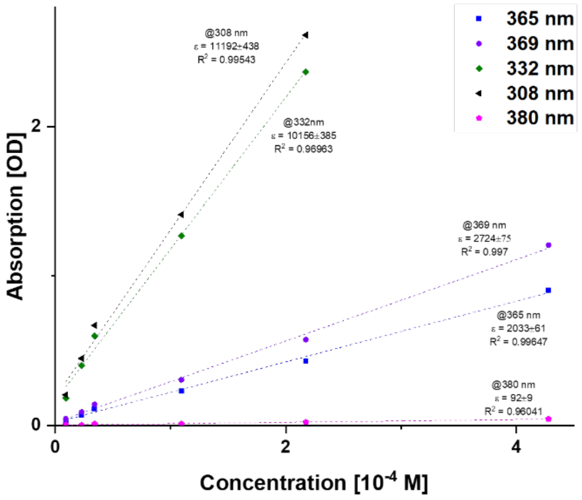

Figure S9. Excitation, emission and emission scan series in the solid state.

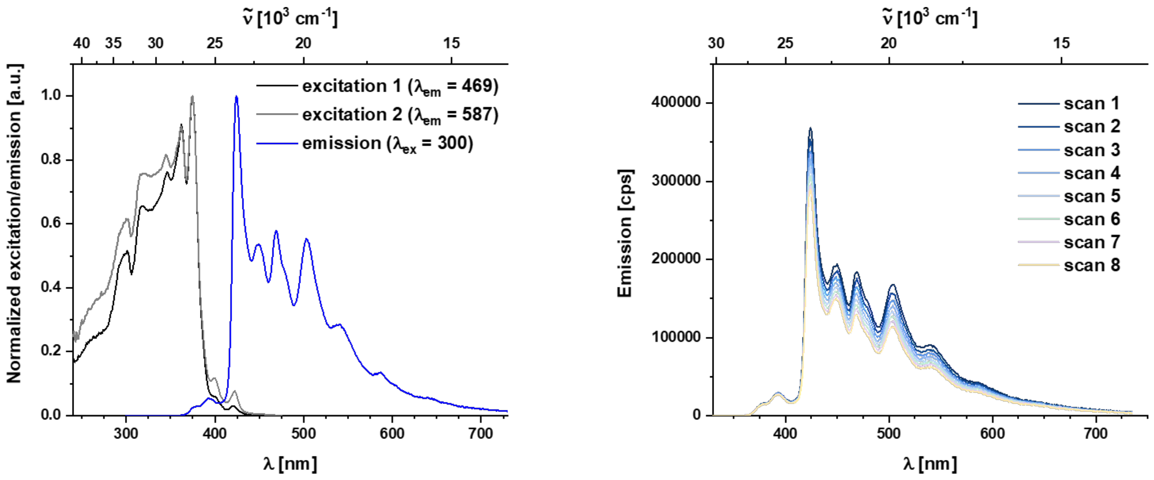

PL spectra of [Ag(IPr)Cbz] (5a):

Figure S10. Absorption, excitation and emission in thf solution.

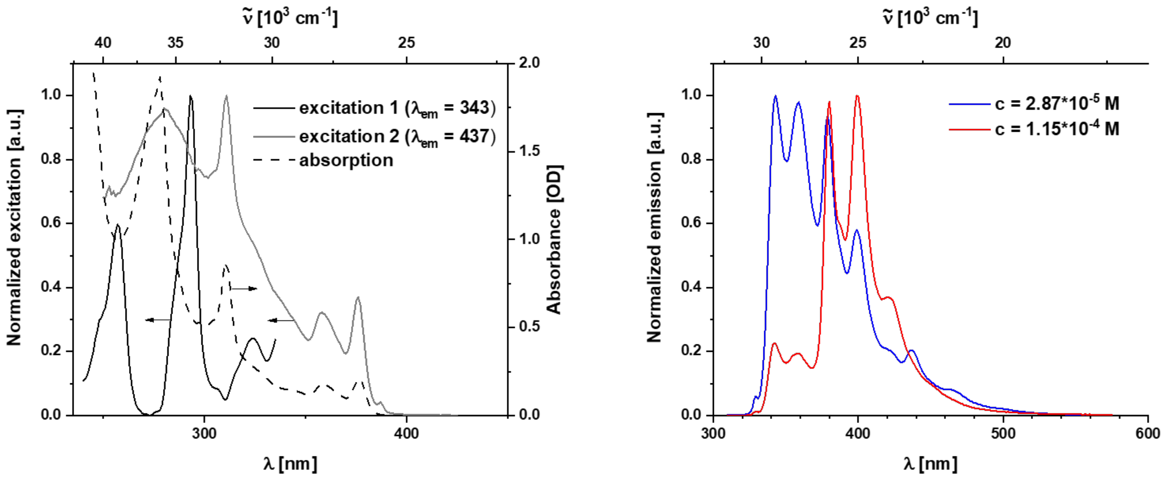

Figure S11. Excitation, emission and emission scan series in the solid state.

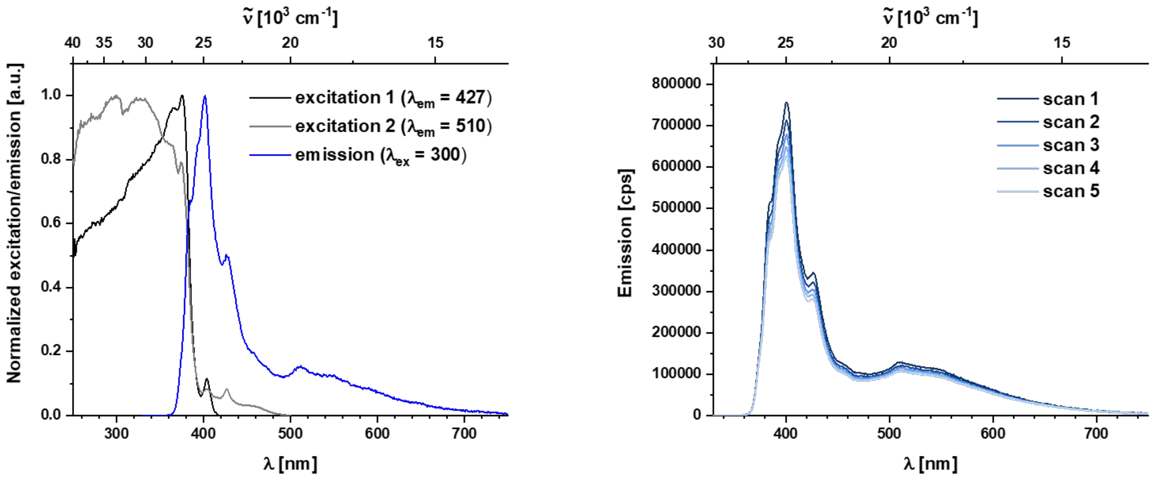

PL spectra of [Ag(SIPr)Cbz] (5b):

Figure S12. Absorption, excitation and emission scan series in thf solution.

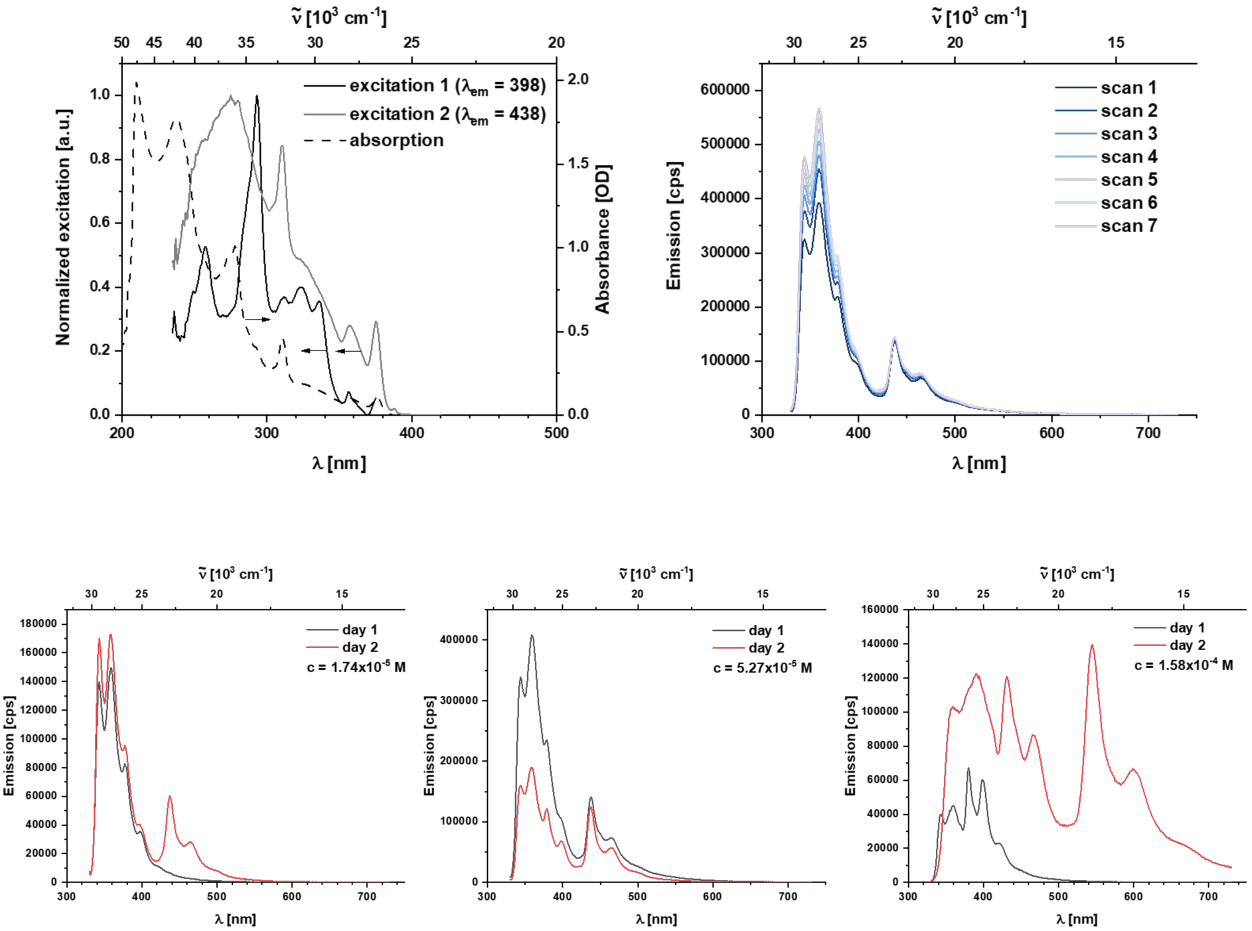

Figure S13. Excitation, emission and emission scan series in the solid state.

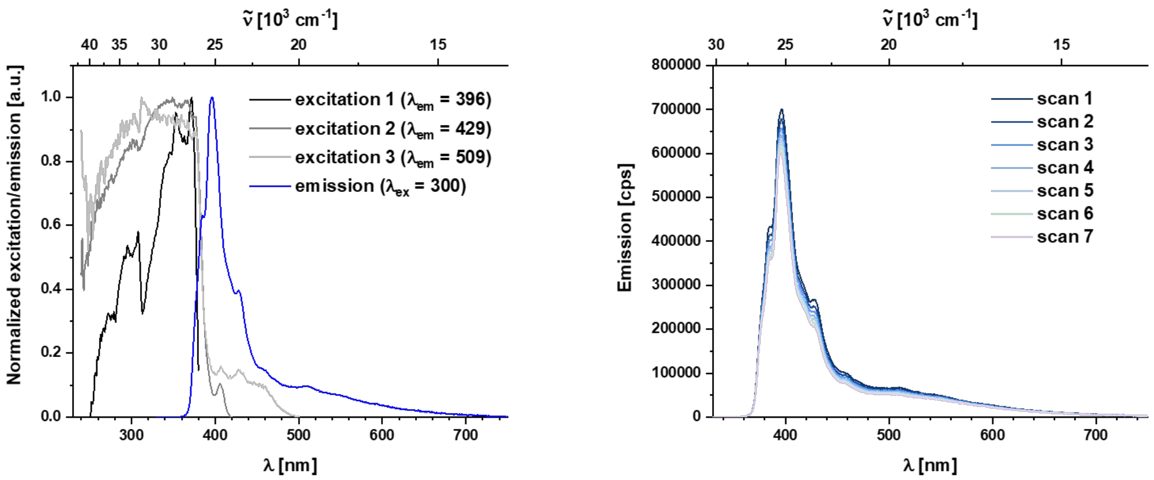

Figure S14. Calculated UV/vis absorption spectrum and most relevant transition density differences (blue: loss of electron density, gold: gain of electron density) for [Au(SIPr)(Cbz)] (**4b**) at the PBE-D3BJ/def2-TZVP//PBE0-D3BJ/def2-SVP/CPCM(THF) level of theory.

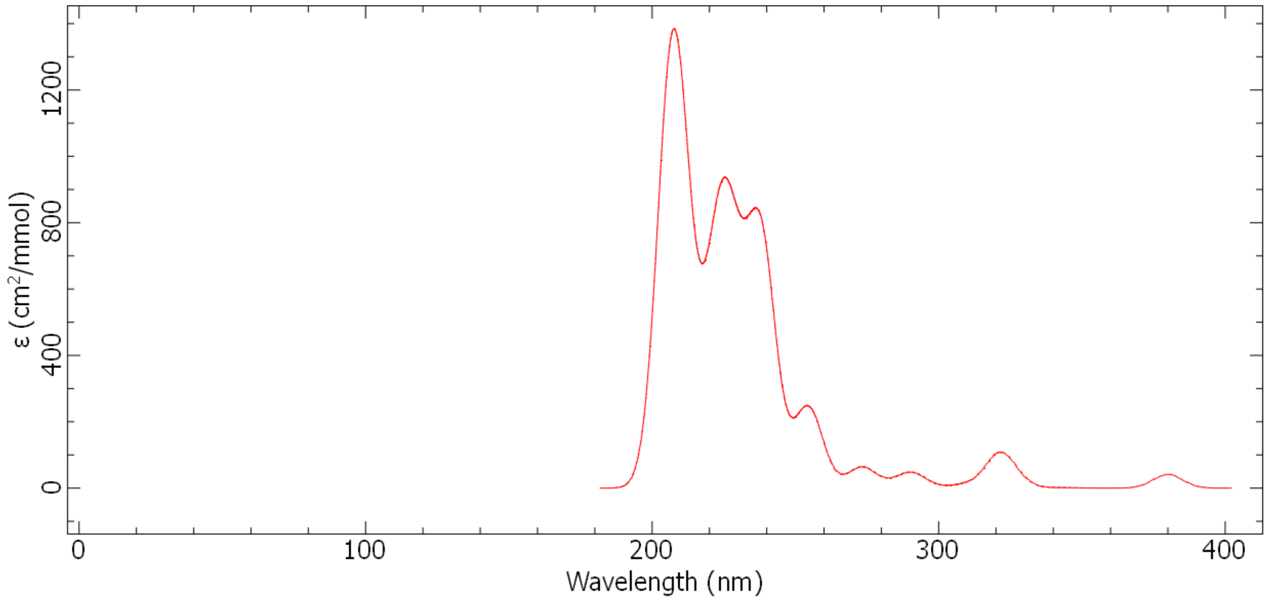

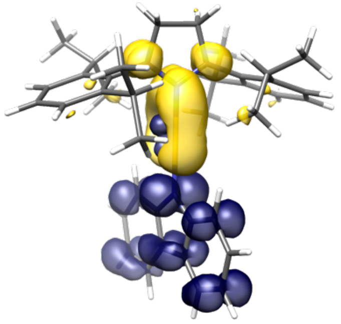

S$_1$ (380 nm, f = 0.019)

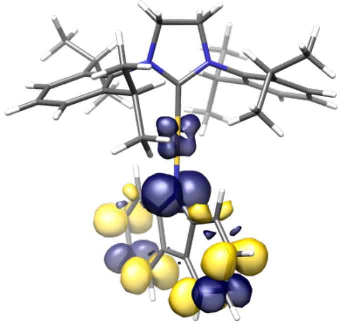

S$_5$ (321 nm, f = 0.042)

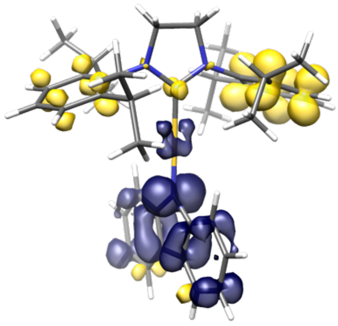

S$_9$ (290 nm, f = 0.012)

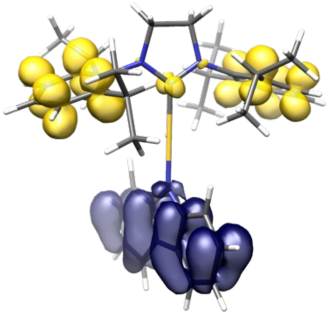

S$_{12}$ (273 nm, f = 0.028)

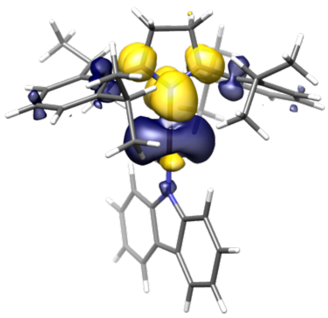

S$_{13}$ (255 nm, f = 0.026)

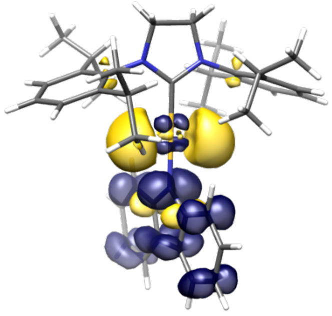

S$_{14}$ (254 nm, f = 0.082)

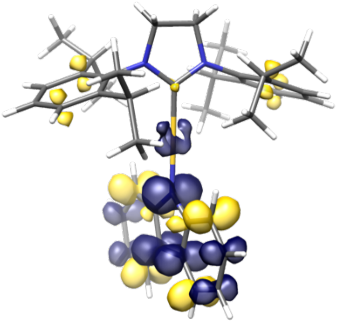

S_{18} (238 nm, f = 0.311)

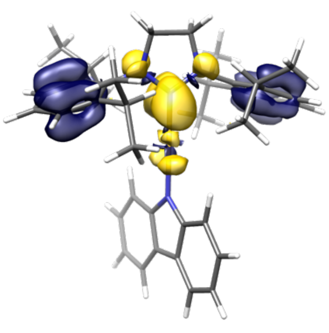

S_{23} (233 nm, f = 0.041)

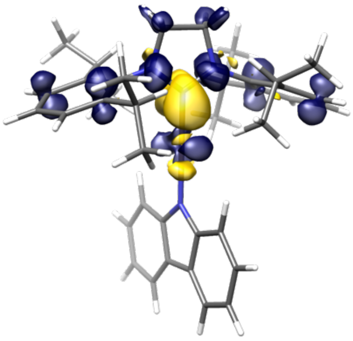

S_{27} (226 nm, f = 0.156)

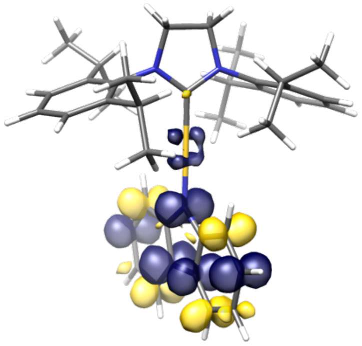

S_{29} (223 nm, f = 0.137)

Figure S15. Transition density differences (blue: loss of electron density, gold: gain of electron density) of selected excited states at the respective optimized T_1 geometry.

[Au(SIPr)(Cbz)] (4b)

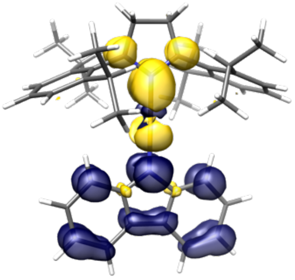

T_1 (434 nm)

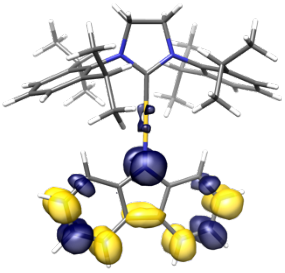

T_2 (425 nm)

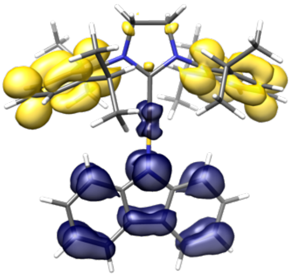

S_1 (378 nm)

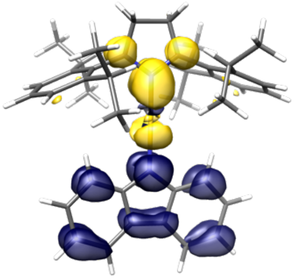

S_2 (342 nm)

[Ag(IPr)(Cbz)] (5a)

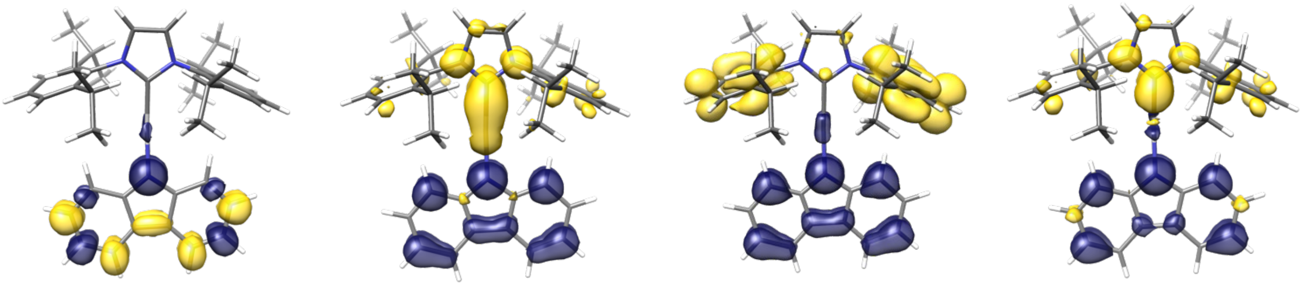

T_1 (443 nm) T_2 (412 nm) S_1 (364 nm) S_2 (351 nm)

[Ag(SIPr)(Cbz)] (5b)

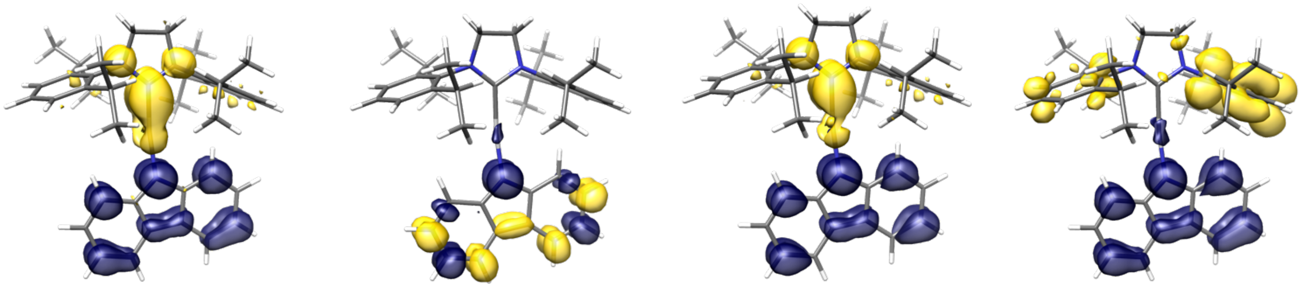

T_1 (455 nm) T_2 (440 nm) S_1 (390 nm) S_2 (369 nm)

Figure S16. ^1H NMR spectra of the photocatalytic reactions of [Au(SIPr)(cbz)] (**4b**) with (E,E*)-dicinnamyl ether, measured in $CDCl_3$ at 500 MHz; top: aromatic region; bottom: aliphatic region.

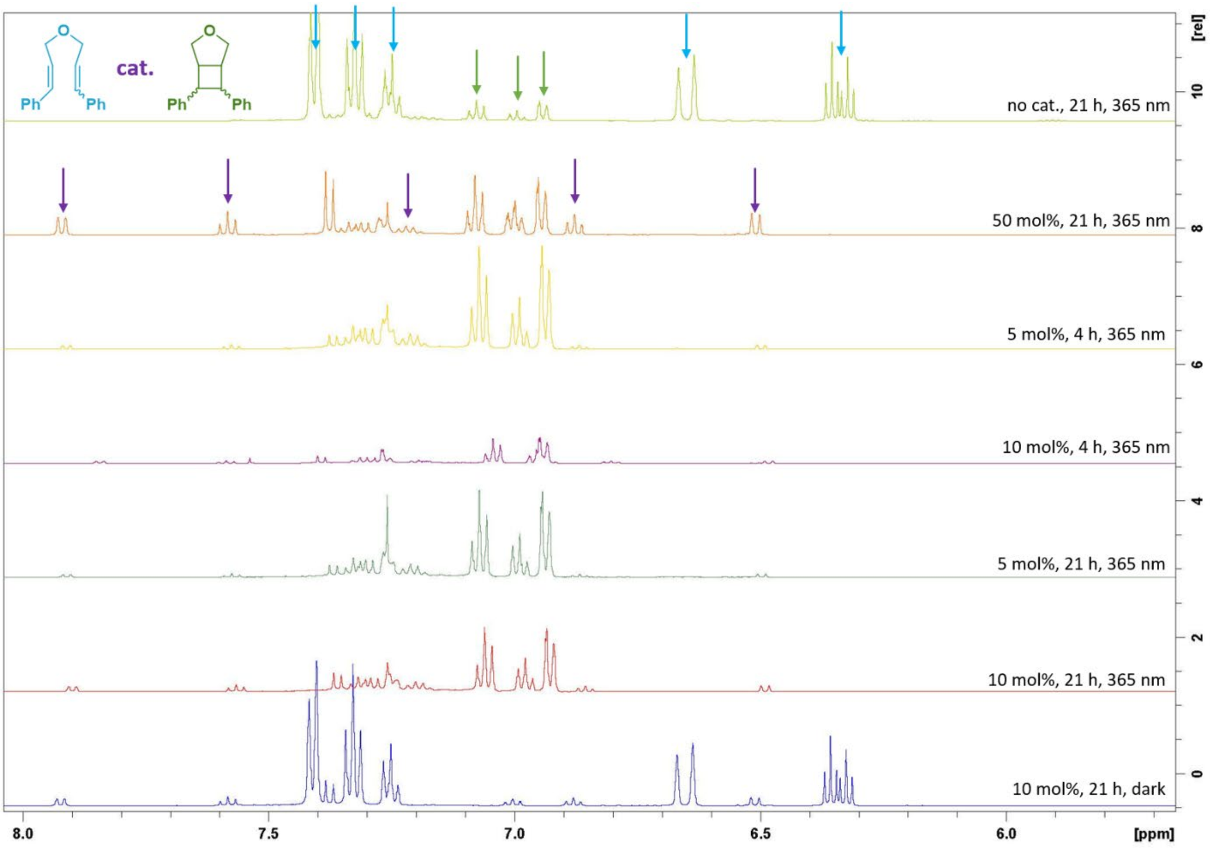

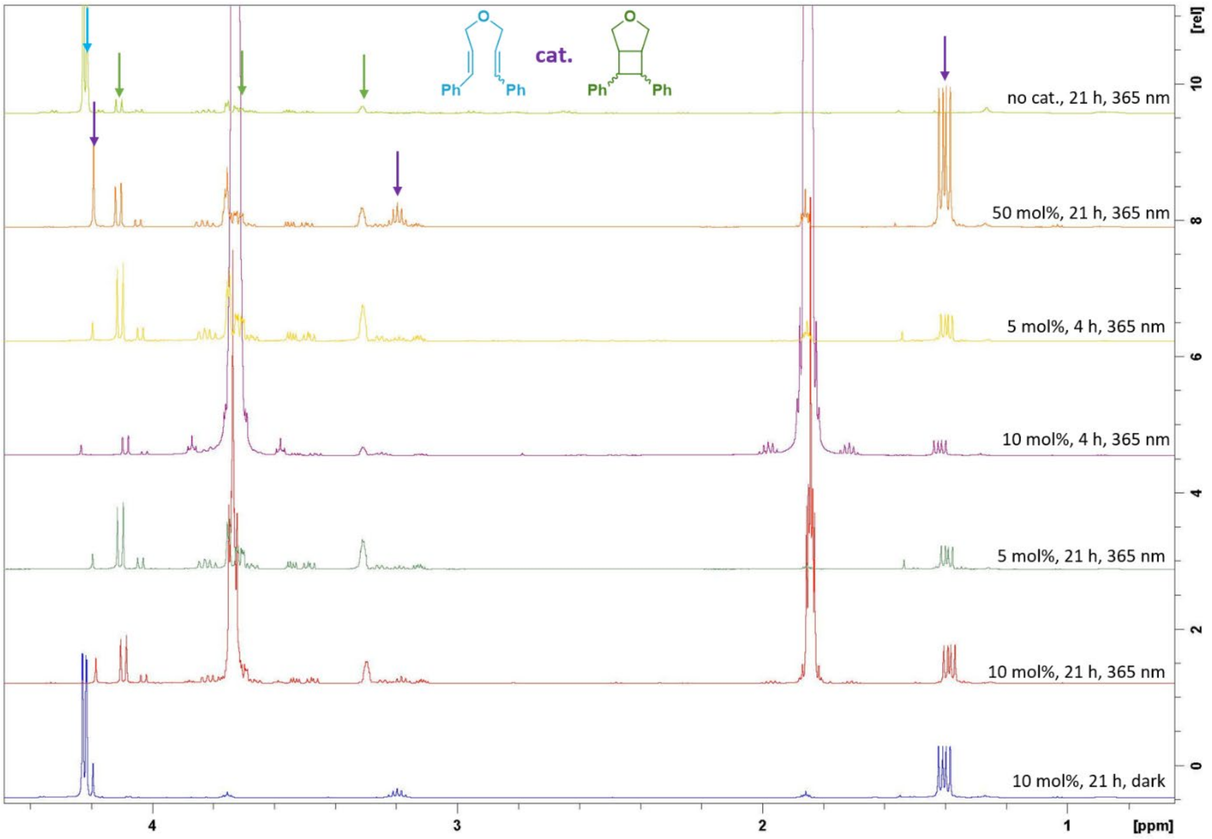

References

[1] a) P. de Frémont, N. M. Scott, E. D. Stevens, S. P. Nolan, *Organometallics* **2005**, *24*, 2411-2418; b) S. R. Patrick, A. Gómez-Suárez, A. M. Z. Slawin, S. P. Nolan, *Organometallics* **2013**, *33*, 421-424; c) A. Collado, A. Gomez-Suarez, A. R. Martin, A. M. Z. Slawin, S. P. Nolan, *Chem. Commun.* **2013**, *49*, 5541-5543; .F. Nahra, N.V. Tzouras, A. Collado, S. P. Nolan, *Nat. Protoc.* **2021**, *16,* 1476‑1493.

[2] a) P. de Frémont, N. M. Scott, E. D. Stevens, T. Ramnial, O. C. Lightbody, C. L. B. Macdonald, J. A. C. Clyburne, C. D. Abernethy, S. Nolan, *Organometallics* **2005**, *24*, 6301-6309; b) G. Berthon-Gelloz, M. A. Siegler, A. L. Spek, B. Tinant, J. N. H. Reek, I. E. Markó, *Dalton Trans.* **2010**, *439*, 1444-1446.

[3] a) O. Santoro, A. Collado, A. M. Z. Slawin, S. P. Nolan, C. S. J. Cazin, *Chem. Commun.* **2013**, *49*, 10483-10485; b) Y. D. Bidal, M. Lesieur, M. Melaimi, F. Nahra, D. B. Cordes, K. S. Athukorala Arachchige, A. M. Z. Slawin, G. Bertrand, C. S. J. Cazin*, Adv. Synth. Catal.*, **2015**, *357*, 3155-3161.

[4] Frisch, M. J.; Trucks, G. W.; Schlegel, H. B.; Scuseria, G. E.; Robb, M. A.; Cheeseman, J. R.; Scalmani, G.; Barone, V.; Mennucci, B.; Petersson, G. A.; Nakatsuji, H.; Caricato, M.; Li, X.; Hratchian, H. P.; Izmaylov, A. F.; Bloino, J.; Zheng, G.;. Sonnenberg, J. L; Hada, M.; Ehara, M.; Toyota, K.; Fukuda, R.; Hasegawa, J.; Ishida, M.; Nakajima, T.; Honda, Y.; Kitao, O.; Nakai, H.; Vreven, T.; Montgomery, J. A.; Peralta, J. E.; Ogliaro, F.; Bearpark, M.; Heyd, J. J.; Brothers, E.; Kudin, K. N.; Staroverov, V. N.; Kobayashi, R.; Normand, J.; Raghavachari, K.; Rendell, A.; Burant, J. C.; Iyengar, S. S.; Tomasi, J.; Cossi, M;. Rega, N.; Millam, J. M.; Klene, M.; Knox, J. E.; Cross, J. B.; Bakken, V.; Adamo, C.; Jaramillo, J.; Gomperts, R.; Stratmann, R. E.; Yazyev, O.; Austin, A. J.; Cammi, R.; Pomelli, C.; Ochterski, J. W. R.; Martin, L.; Morokuma, K.; Zakrzewski, V. G.; Voth, G. A.; Salvador, P.; Dannenberg, J. J.; Dapprich, S.; Daniels, A. D.; Farkas, Ö.; Foresman, J. B.; Ortiz, J. V.; Cioslowski, J.; Fox D. J. Gaussian 09 Revision A.1, Gaussian, Inc., Wallingford, CT, 2009.

[5] a) Adamo, C.; Barone, V., J. Chem. Phys. 1999, 110, 6158-6170, b) Grimme, S., *J. Comp. Chem*. **2004**, *25*, 1463-1473

[6] Schaefer, A.; Horn, H.; Ahlrichs, R. J. Chem. Phys. 1992, 97, 2571-2577.

[7] a) Wadt, W. R.; Hay, P. J. J. Chem. Phys. 1985, 82, 284-298; b) Hay, P. J ; Wadt, W. R. *J. Chem. Phys*. **1985**, *82*, 299-310.

[8] a) Tomasi, J.; Persico, M. Chem. Rev. 1994, 94, 2027–2094; b) Barone, V.; Cossi, M. *J. Phys. Chem. A* **1998**, *102*, 1995–2001.

[9] F. Neese, *WIREs Comput. Mol. Sci.* **2012**, *2*, 73–78.

[10] a) J. P. Perdew, K. Burke, M. Ernzerhof, *Phys. Rev. Lett.* **1996**, *77*, 3865–3868; b) J. P. Perdew, K. Burke, M. Ernzerhof, *Phys. Rev. Lett.* **1997**, *78*, 1396; c) J. P. Perdew, K. Burke, M. Ernzerhof, *Phys. Rev. B: Condens. Matter* **1996**, *105*, 9982–9985; d) M. Ernzerhof, G. E. Scuseria, *J. Chem. Phys.* **1999**, *110*, 5029–5036; e) J. Tao, J. P. Perdew, V. N. Staroverov, G. E. Scuseria, *Phys. Rev. Lett.* **2003**, *91*, 146401; f) J. P. Perdew, J. Tao, V. N. Staroverov, G. E. Scuseria, *J. Chem. Phys.* **2004**, *120*, 6898–6911.

[11] F. Weigend, R. Ahlrichs, *Phys. Chem. Chem. Phys.* **2005**, *7*, 3297–3305.

[12] F. Weigend, *Phys. Chem. Chem. Phys.* **2006**, *8*, 1057–1065; b) D. A. Pantazis, F. Neese, *J. Chem. Theory Comput.* **2009**, *5*, 2229–2238; c) D. A. Pantazis, X.-Y. Chen, C. R. Landis, F. Neese, *J. Chem. Theory Comput.* **2008**, *4*, 908–919; d) D. A. Pantazis, F. Neese, *Theor. Chem. Acc.* **2012**, *131*, 1292; e) D. A. Pantazis, F. Neese, *J. Chem. Theory Comput.* **2011**, *7*, 677–684.

[13] a) S. Grimme, J. Antony, S. Ehrlich, H. Krieg, *J. Chem. Phys.* **2010**, *132*, 154104; b) S. Grimme, S. Ehrlich, L. Goerigk, *J. Comput. Chem.* **2011**, *32*, 1456–1465.

[14] E. F. Pettersen, T. D. Goddard, C. C. Huang, G. S. Couch, D. M. Greenblatt, E. C. Meng, T. E. Ferrin, *J. Comput. Chem.* **2004**, *13*, 1605-12.

[15] a) S. Kim, F. D.Toste, *J. Am. Chem. Soc.* **2019**, *141*, 4308-4315; b) N. V. Tzouras, M. Saab, W. Janssens, T. Cauwenbergh, K. Van Hecke, F. Nahra, S. P. Nolan, *Chem. Eur. J.* **2020**, *26*, 5541-5551.

[16] J. Li, L. Wang, Z. Zhao, X. Li, X. Yu, P. Huo, Q. Jin, Z. Liu, Z. Bian, C. Huang, *Angew. Chem. Int. Ed.* **2020**, *59*,8210-8217.

[17] A. Gómez-Suárez, D. J. Nelson, D. G. Thompson, D. B. Cordes, D. Graham, A. M. Z. Slawin, S. P. Nolan, *Beilstein J. Org. Chem.* **2013**, *9*, 2216-2223.

NMR Spectra

^{1}H NMR and ^{13}C {^{1}H} NMR for [Au(IPr)Cbz] (4a):

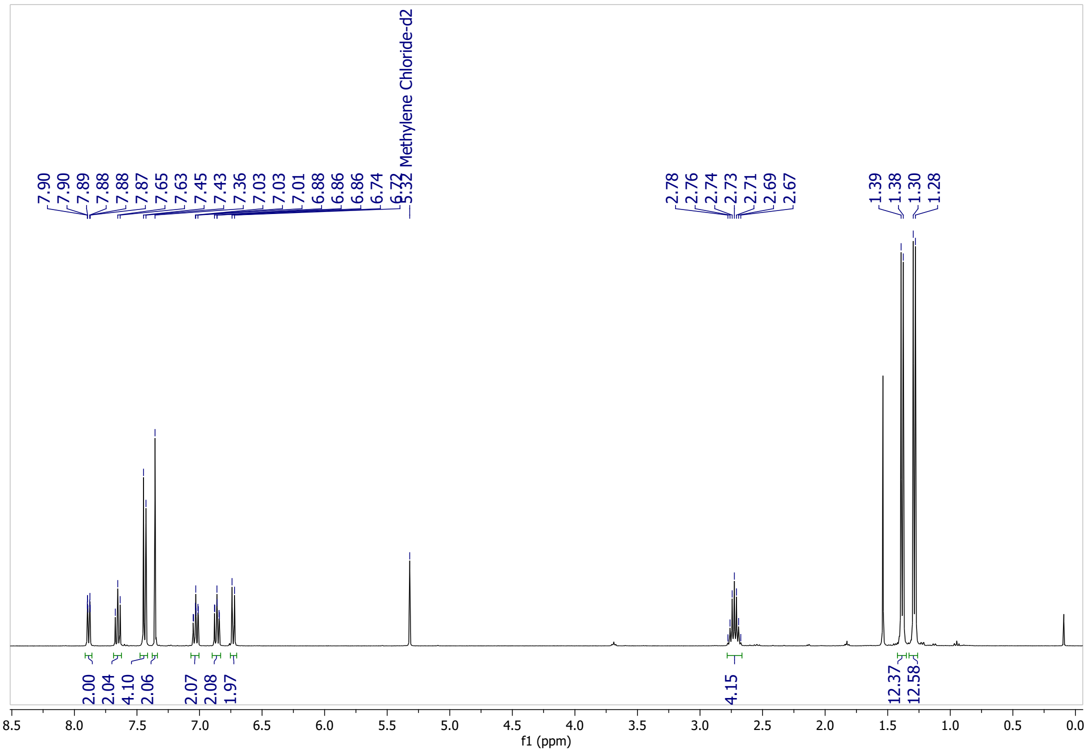

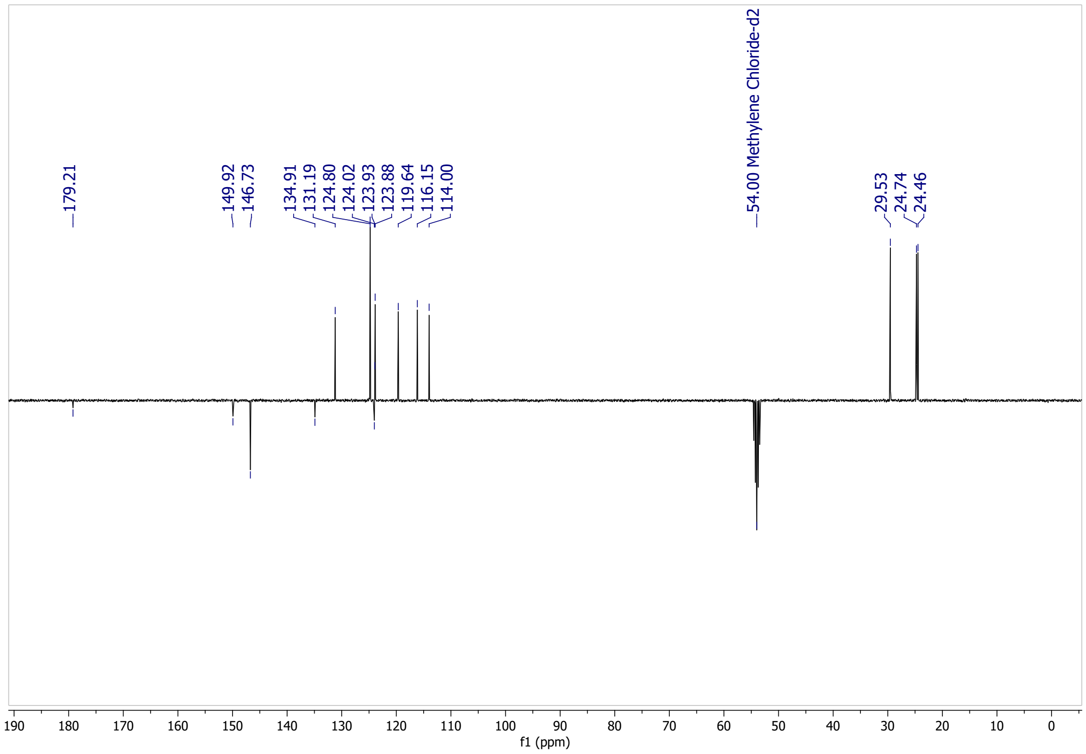

1H NMR and 13C {1H} NMR for [Au(SIPr)Cbz] (4b):

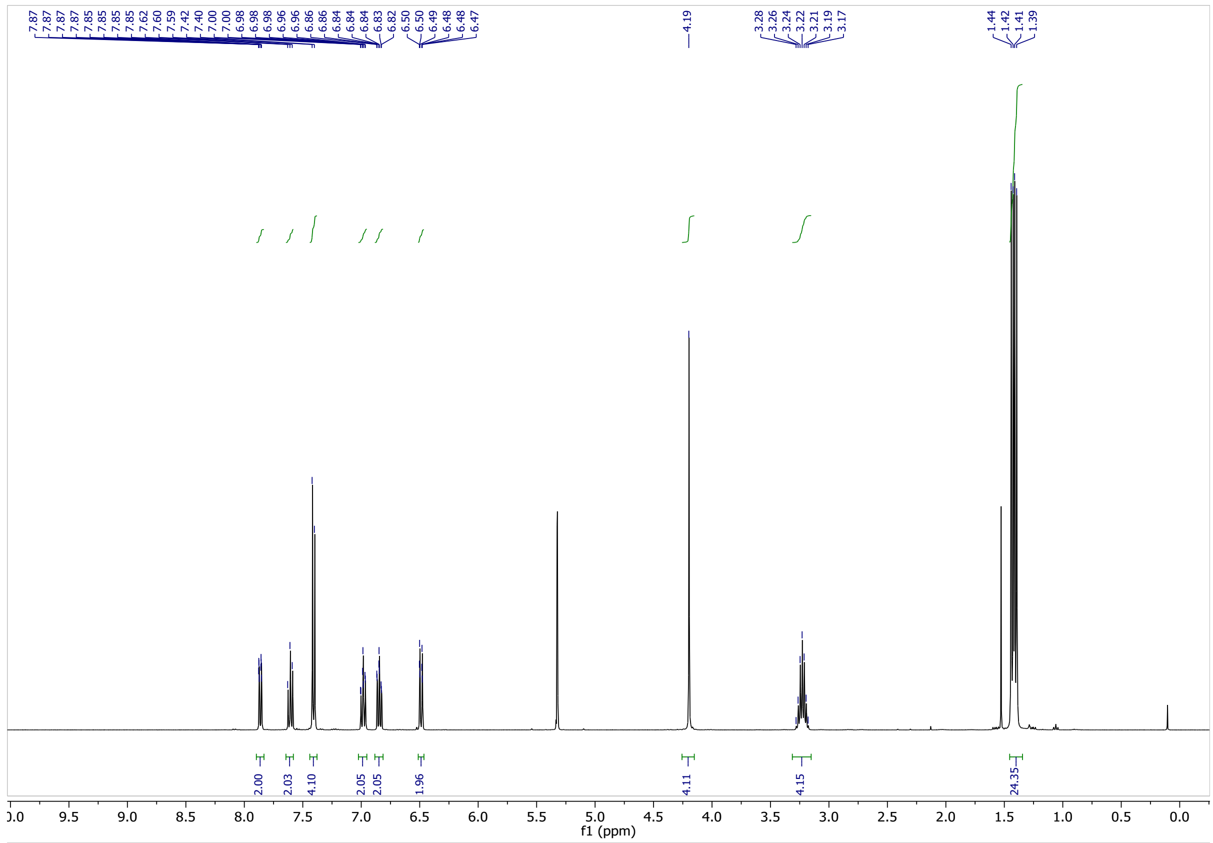

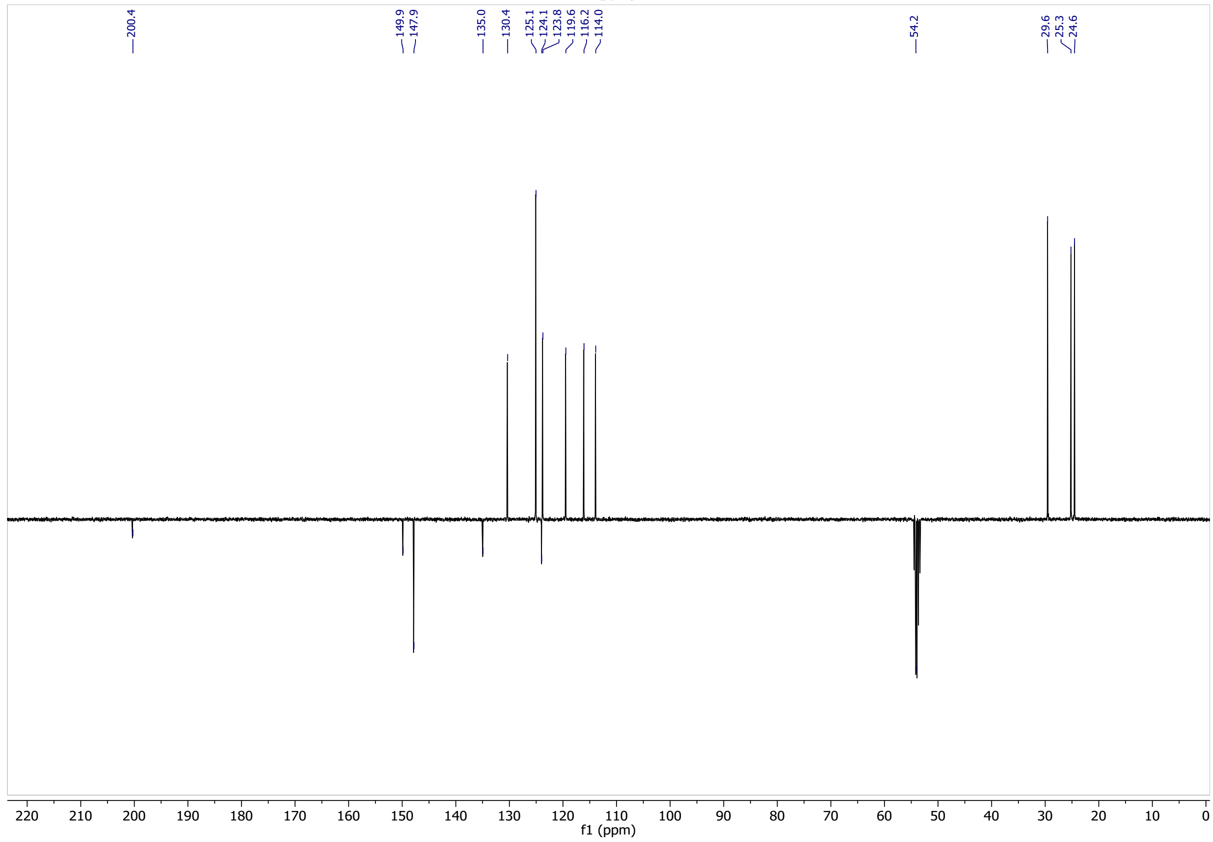

^{1}H NMR and ^{13}C {^{1}H} NMR for [Au(IPr*)Cbz] (4c):

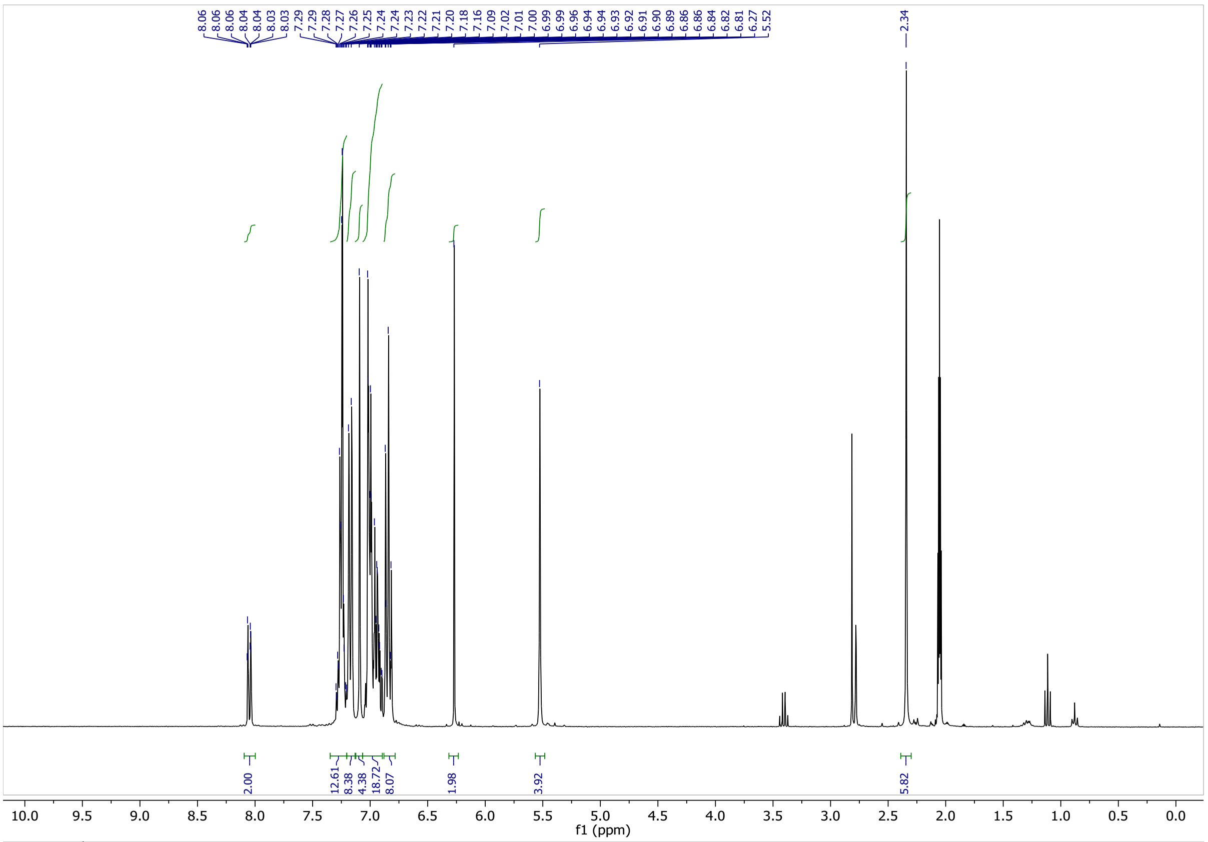

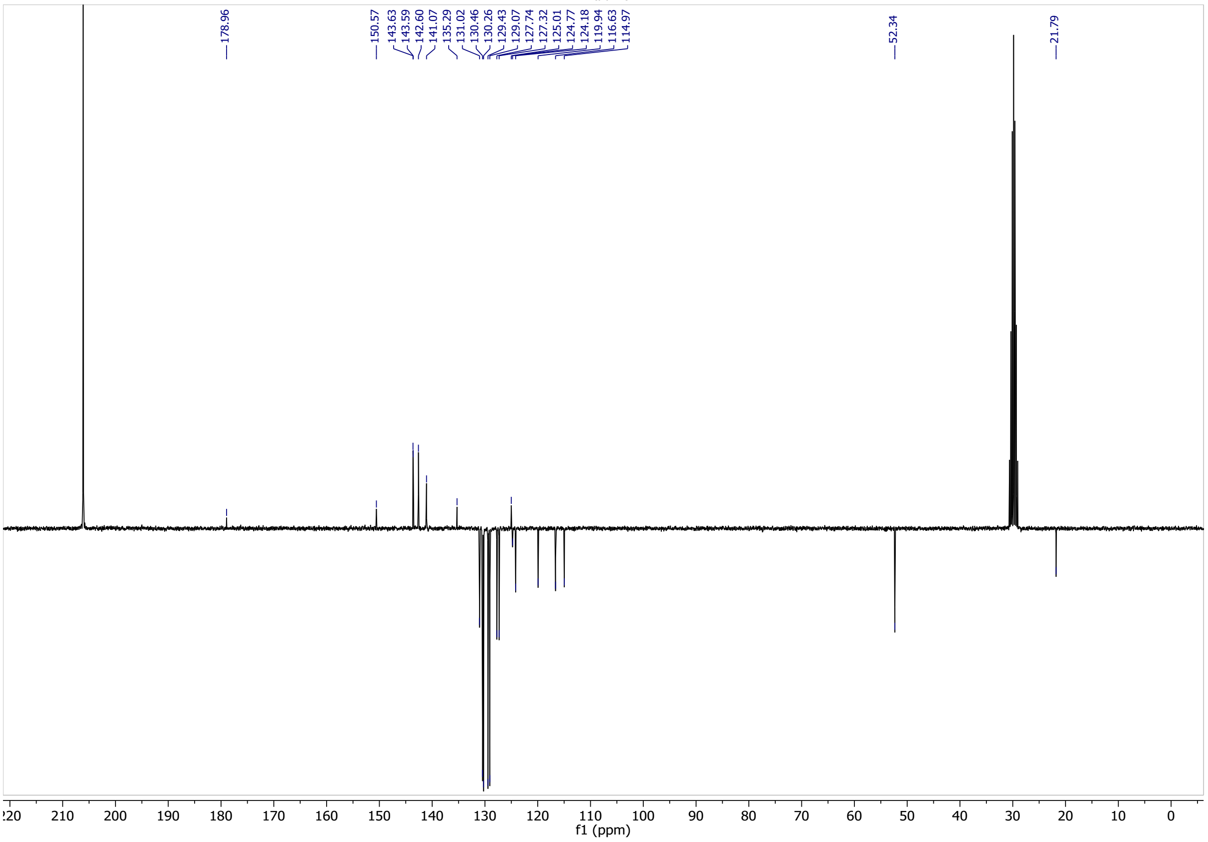

¹H NMR and ¹³C {¹H} NMR for [Au(IPr^{Cl})Cbz] (4d):

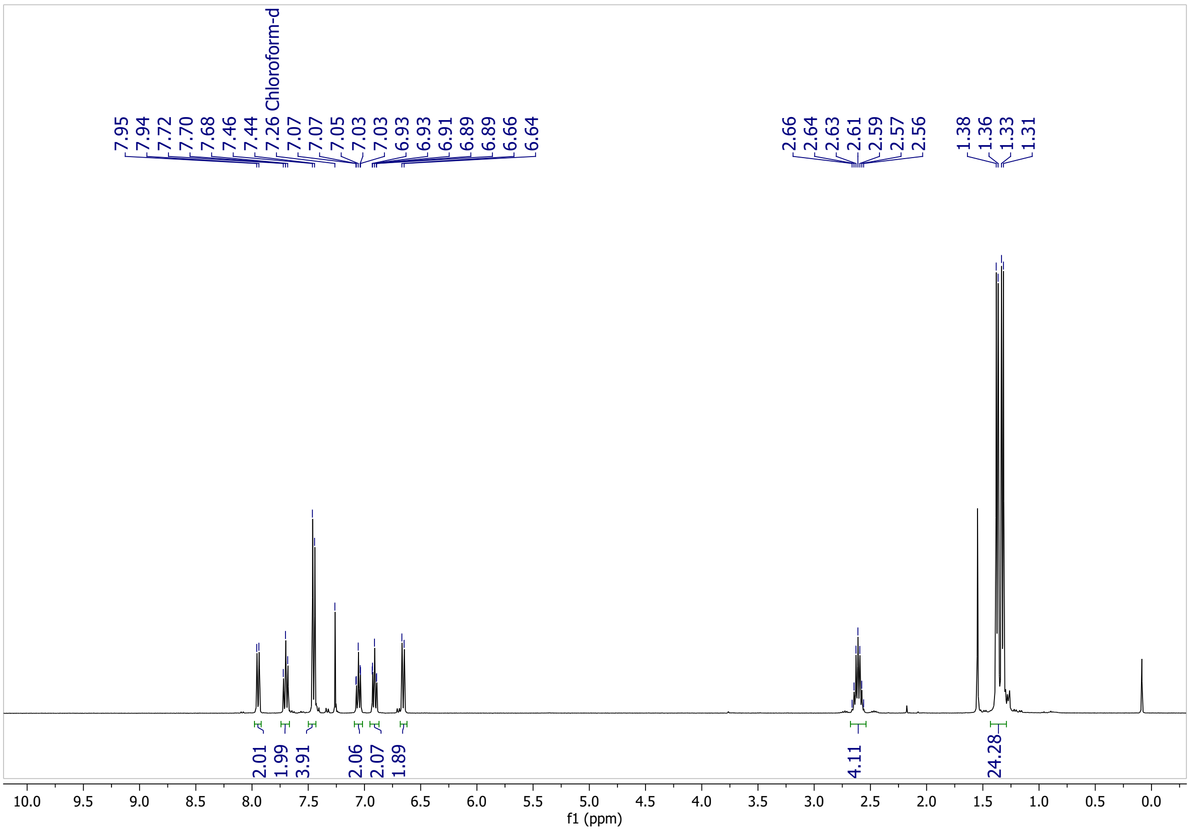

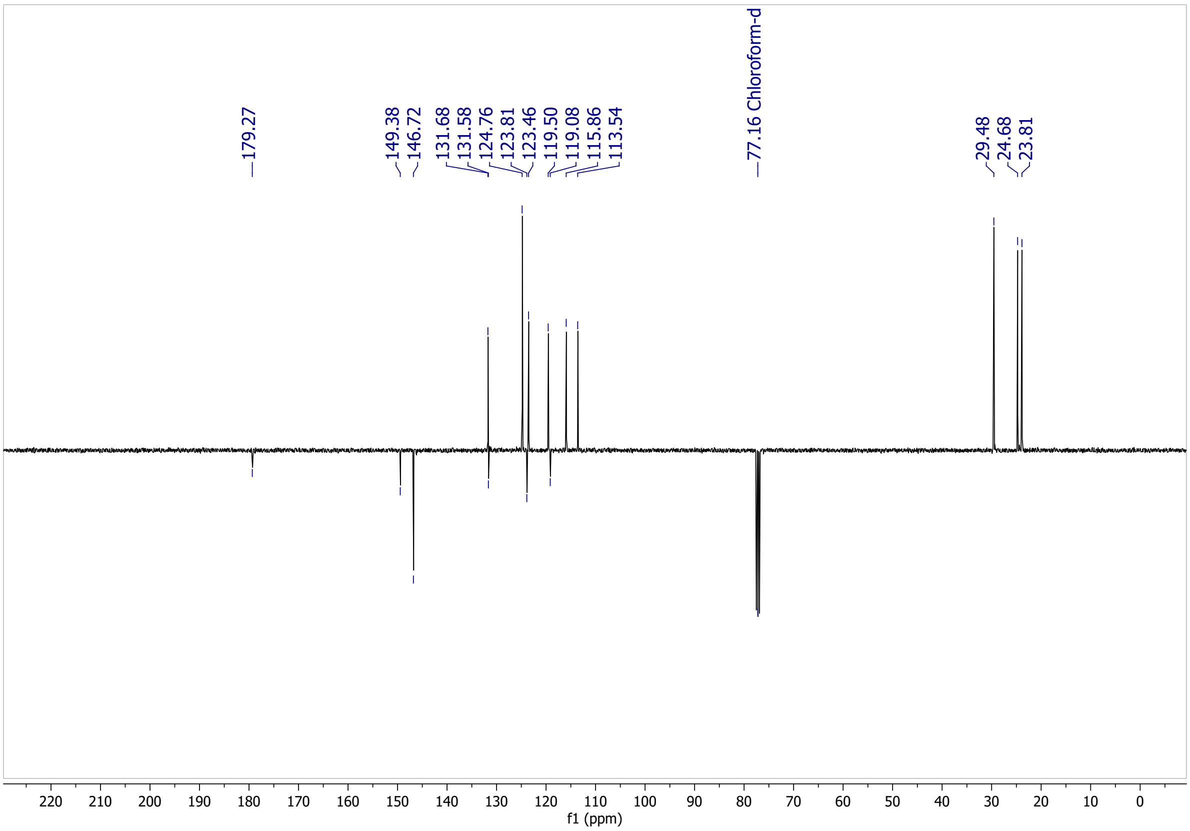

^{1}H NMR and ^{13}C {^{1}H} NMR for [Au(ICy)Cbz] (4e):

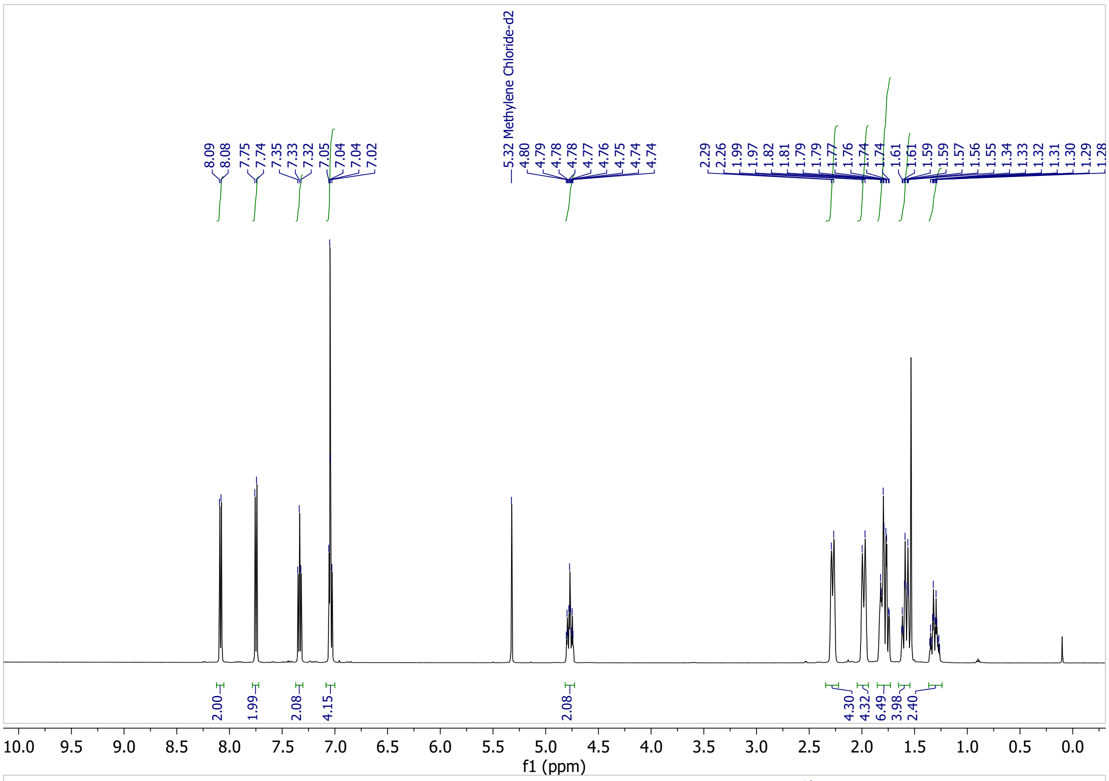

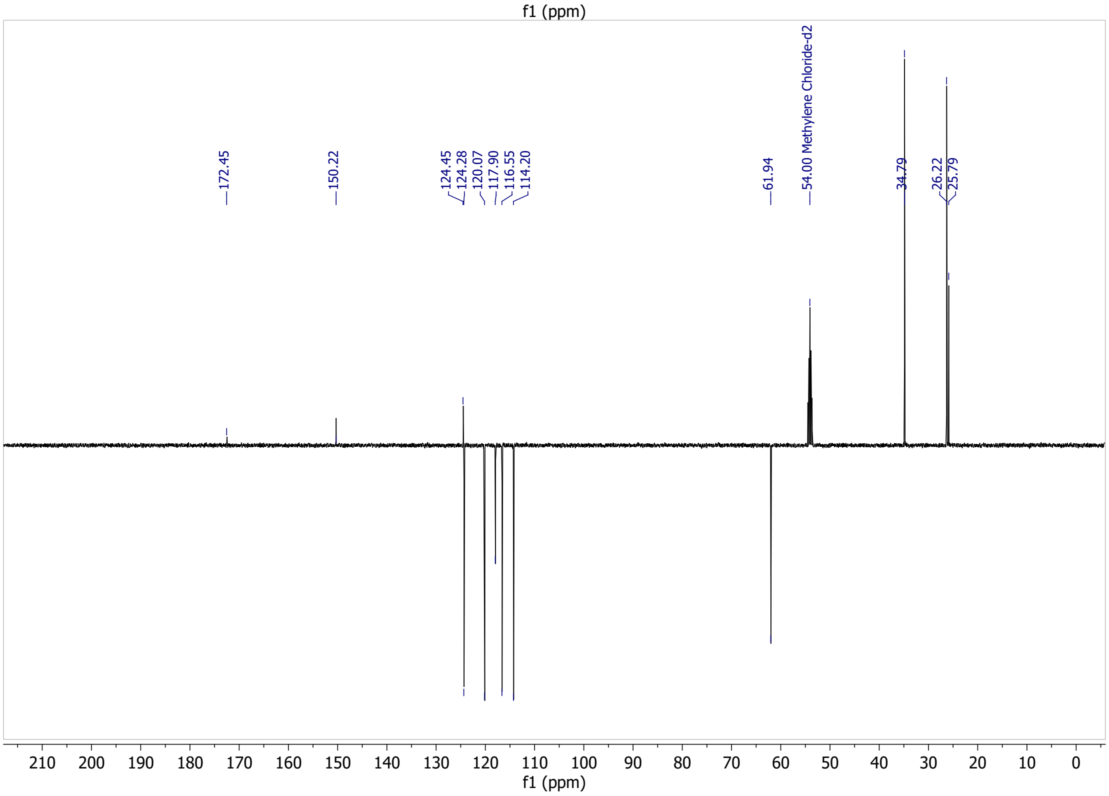

^{1}H NMR and ^{13}C {^{1}H} NMR for [Au(IAd)Cbz] (4f):

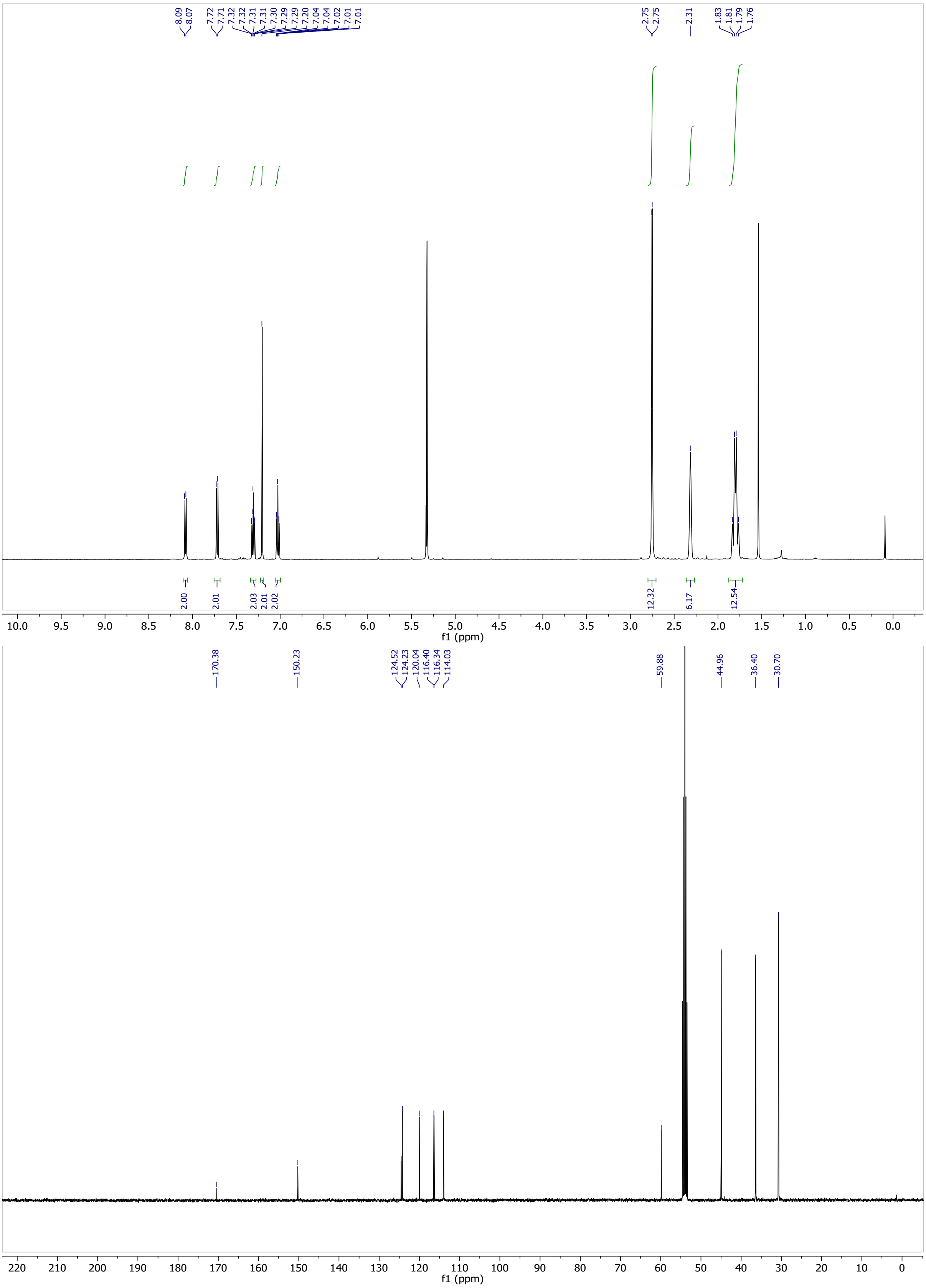

SUPPORTING INFORMATION

^{1}H NMR and ^{13}C {^{1}H} NMR for [Au(I^{t}Bu)Cbz] (4g):

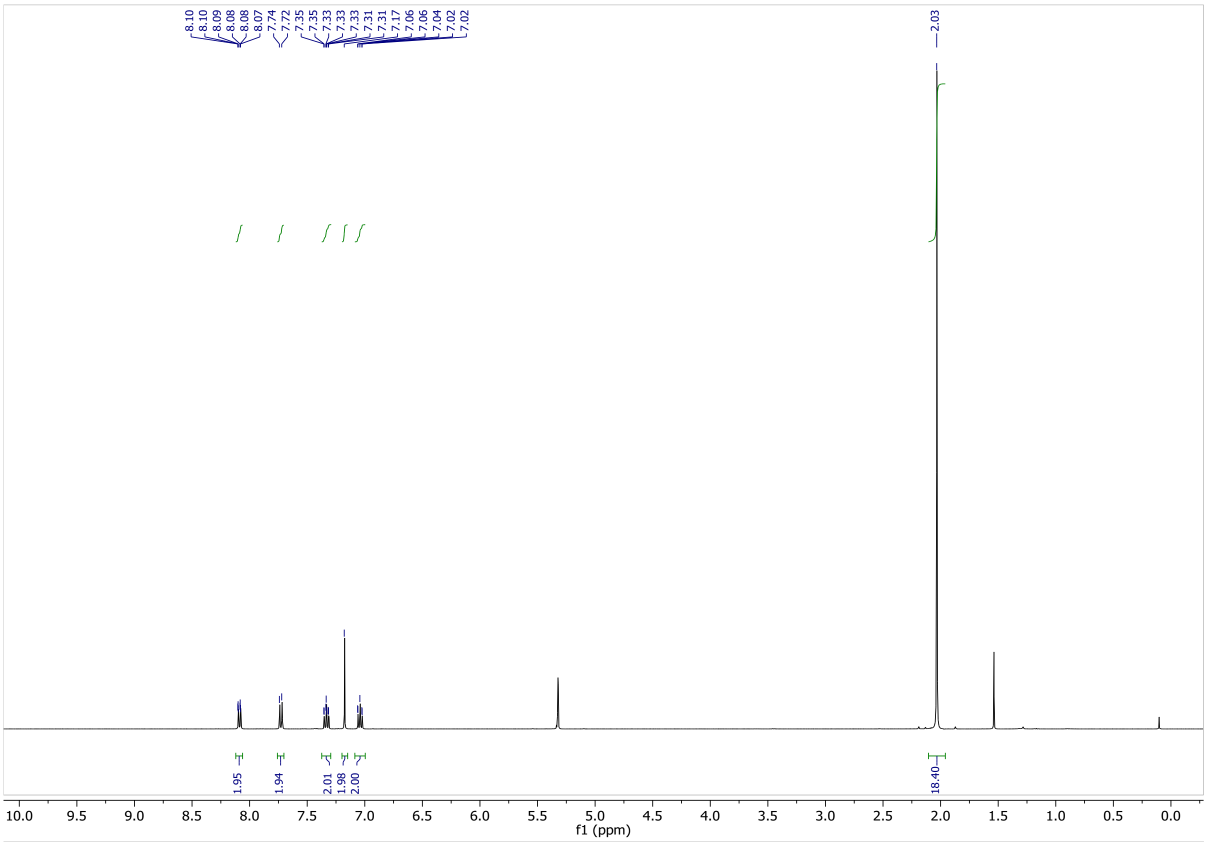

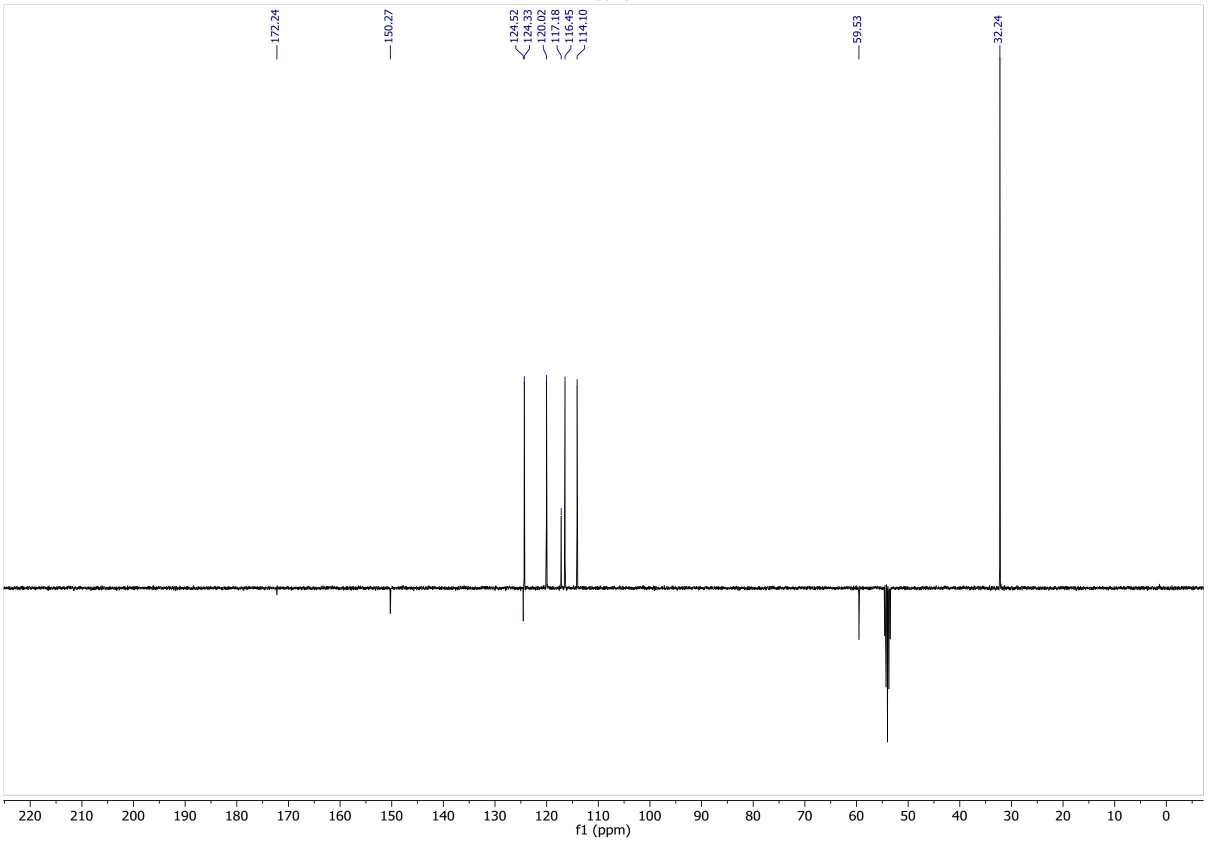

^{1}H NMR, ^{13}C {^{1}H} NMR and 2D-HMBC for [Ag(IPr)Cbz] (5a):

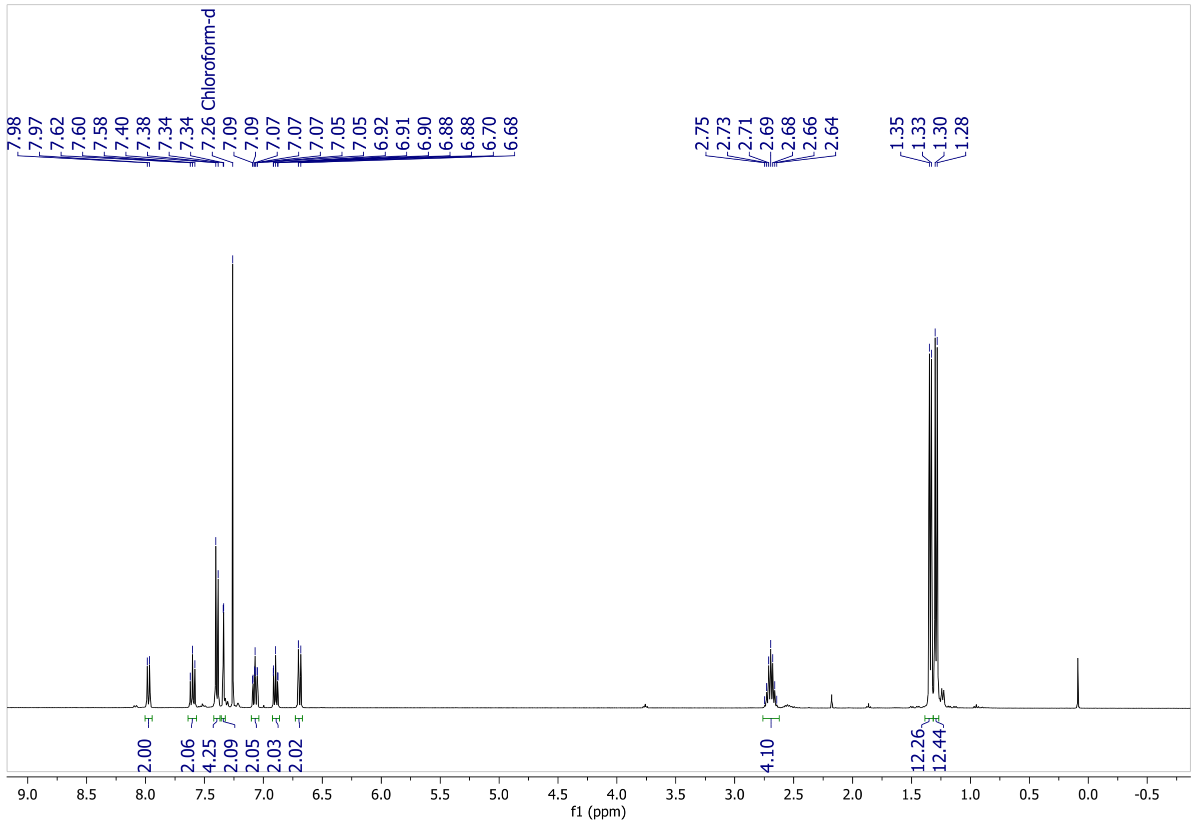

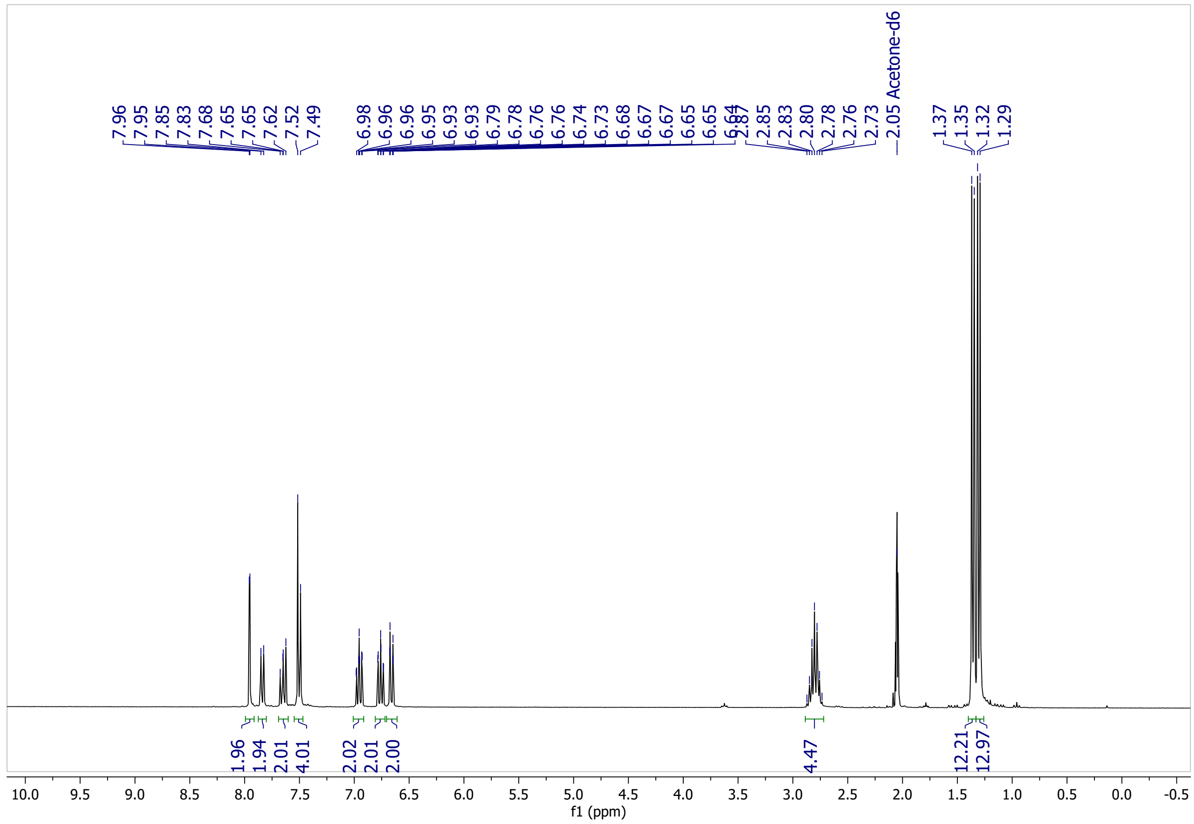

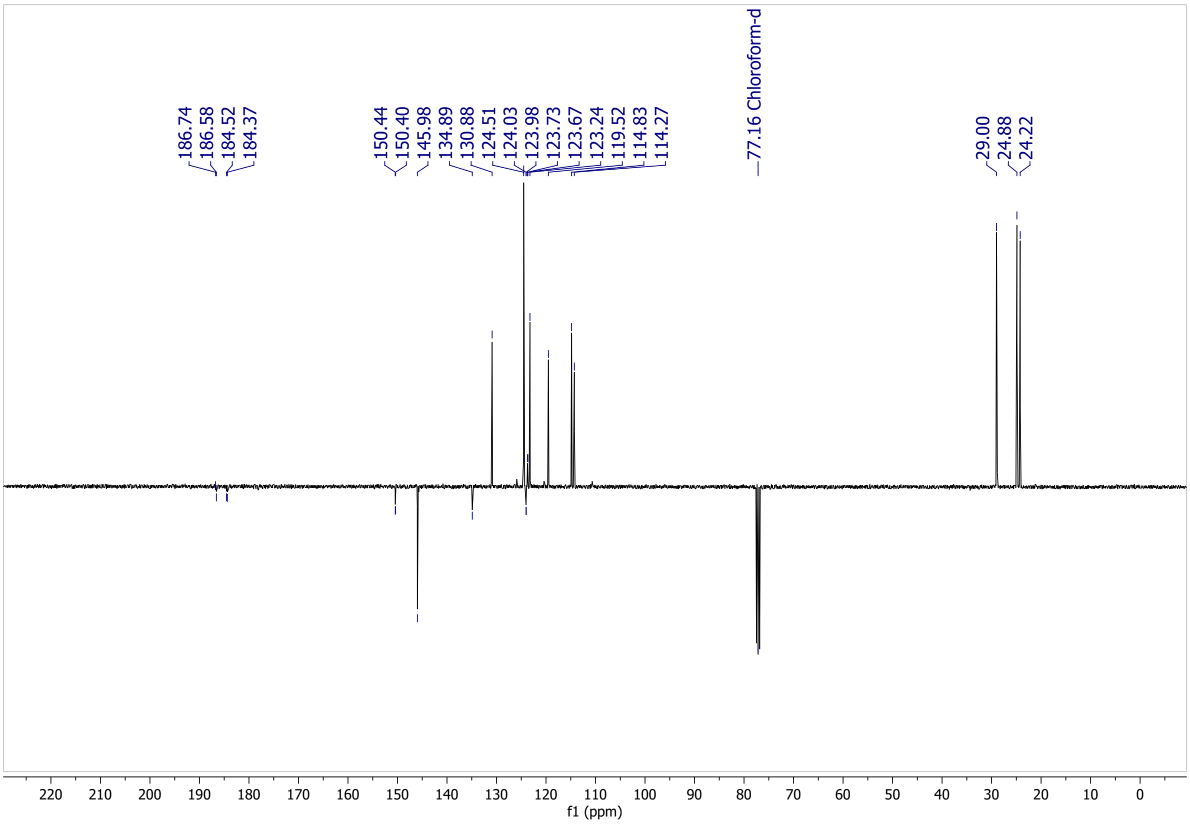
186.74
186.58
184.52
184.37
150.44
150.40
145.98
134.89
130.88
124.51
124.03
123.98
123.73
123.67
123.24
119.52
114.83
114.27
77.16 Chloroform-d
29.00
24.88
24.22
f1 (ppm)

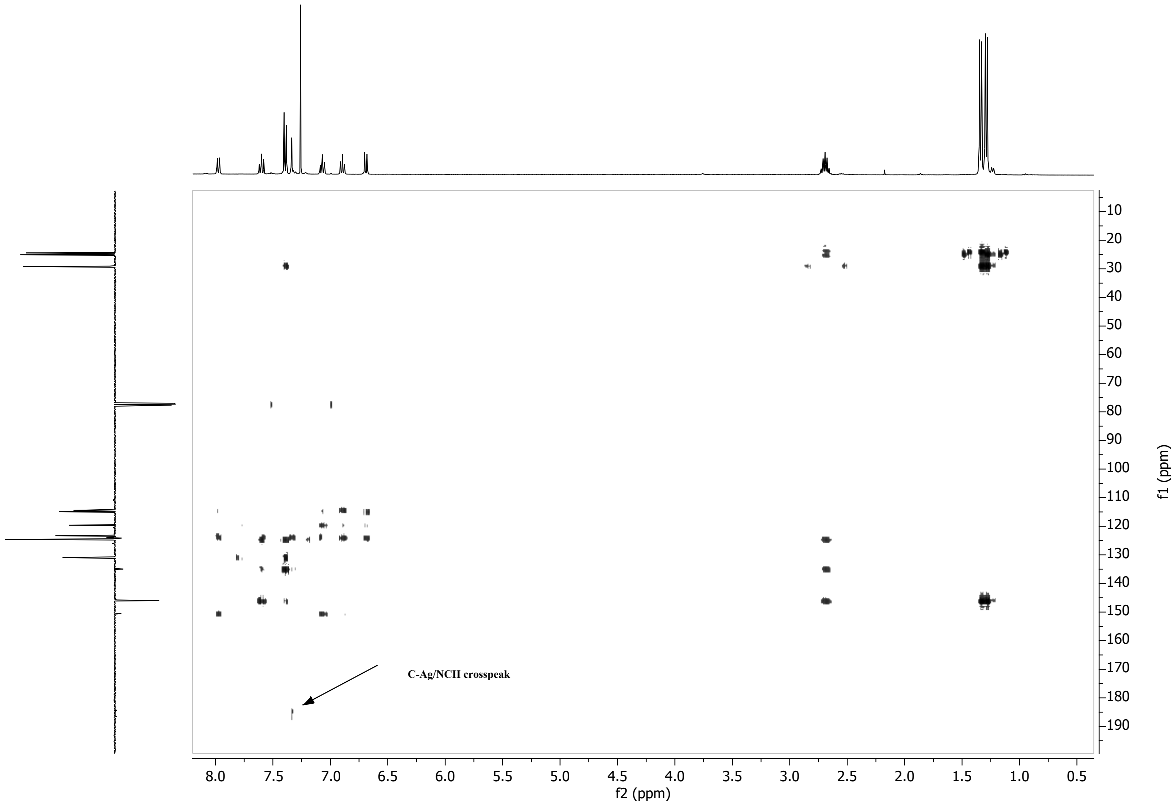
C-Ag/NCH crosspeak
f2 (ppm)
f1 (ppm)

^{1}H NMR, ^{13}C {^{1}H} NMR and 2D-HMBC for [Ag(SIPr)Cbz] (5b):

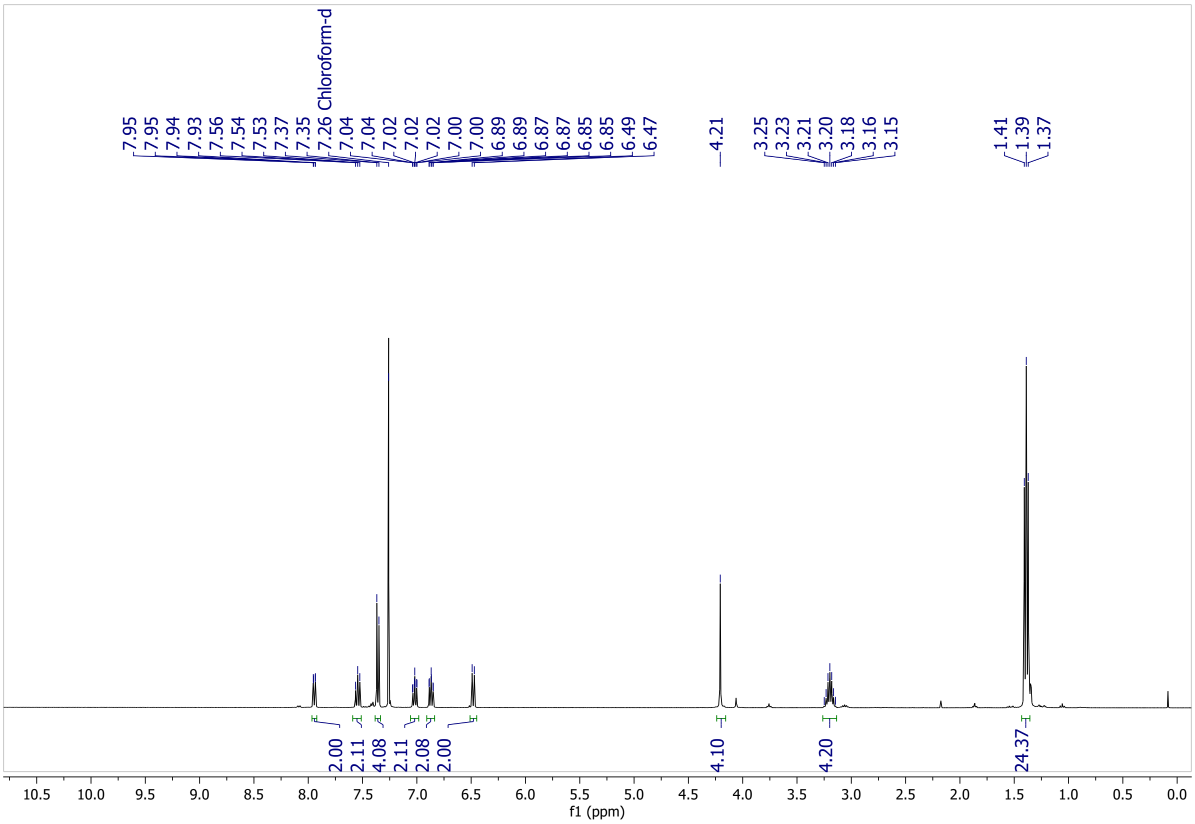

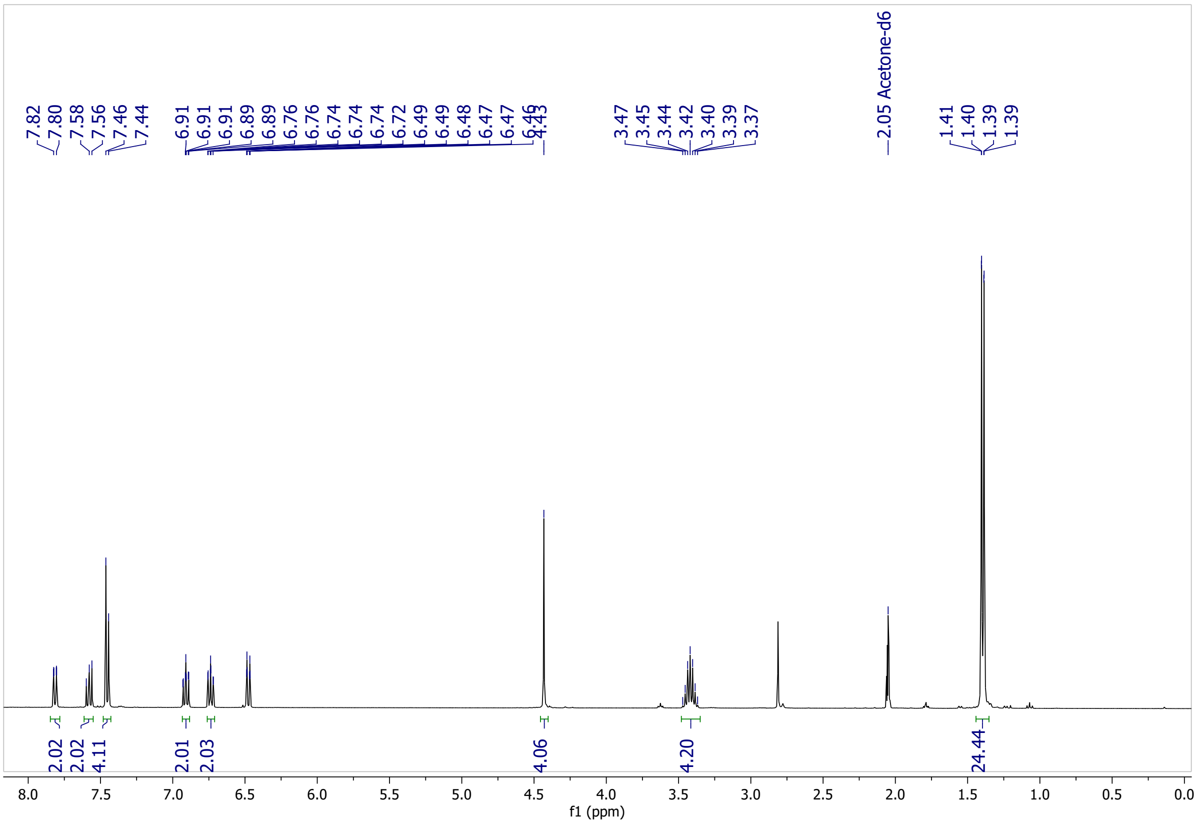

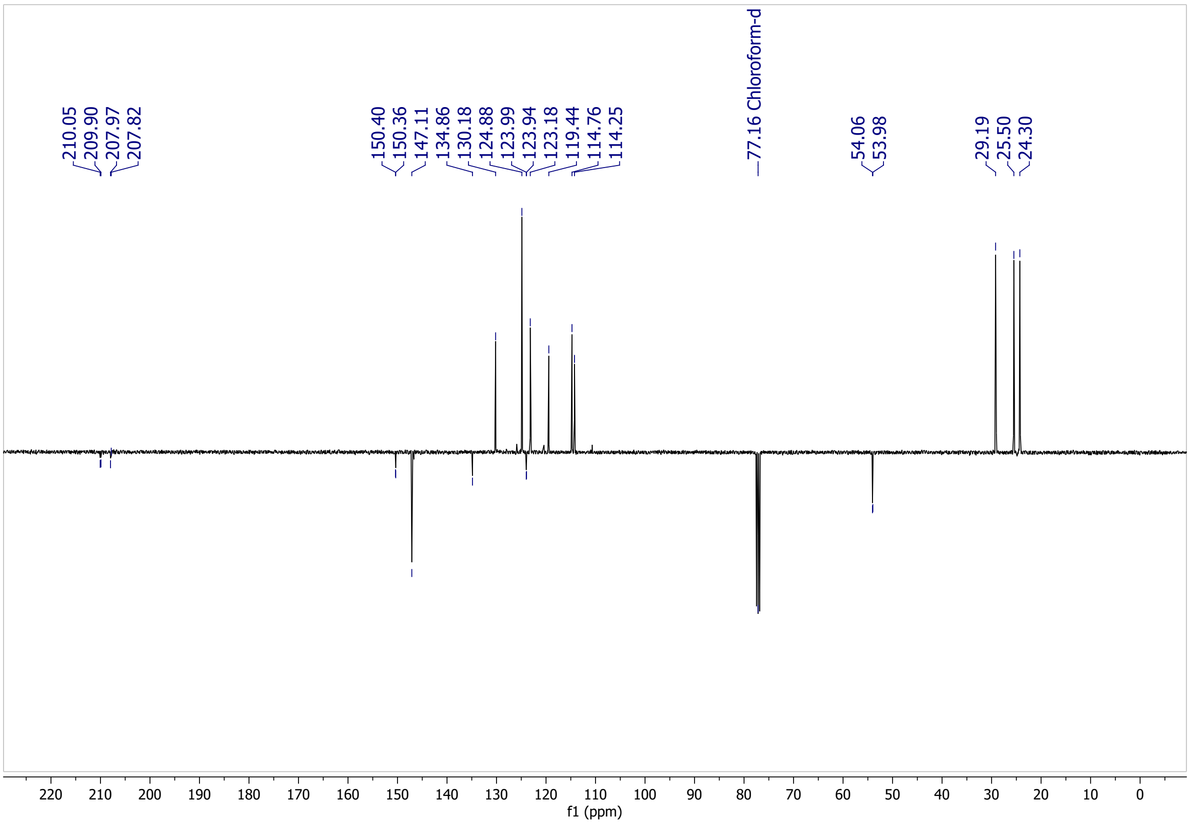
210.05
209.90
207.97
207.82
150.40
150.36
147.11
134.86
130.18
124.88
123.99
123.94
123.18
119.44
114.76
114.25
77.16 Chloroform-d
54.06
53.98
29.19
25.50
24.30
f1 (ppm)

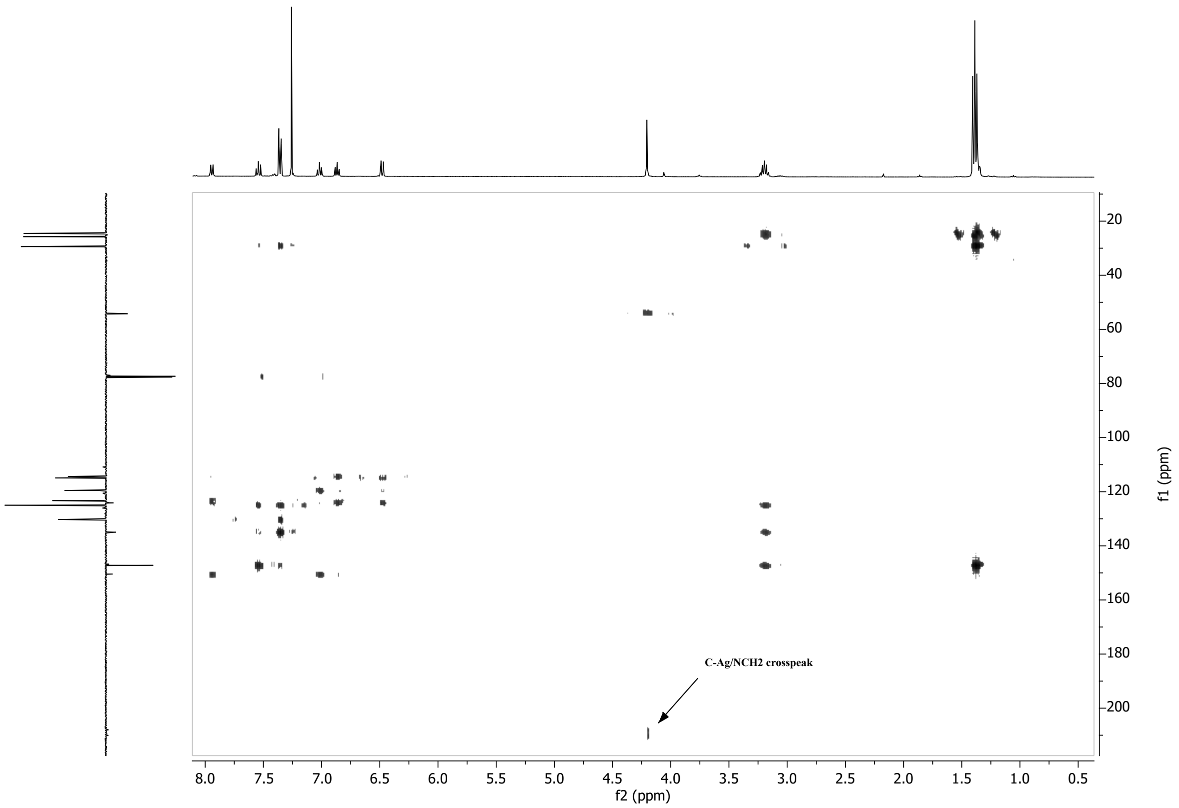
C-Ag/NCH2 crosspeak
f2 (ppm)
f1 (ppm)

^{1}H NMR, ^{13}C {^{1}H} NMR and 2D-HMBC for [Ag(IPr*))Cbz] (5c):

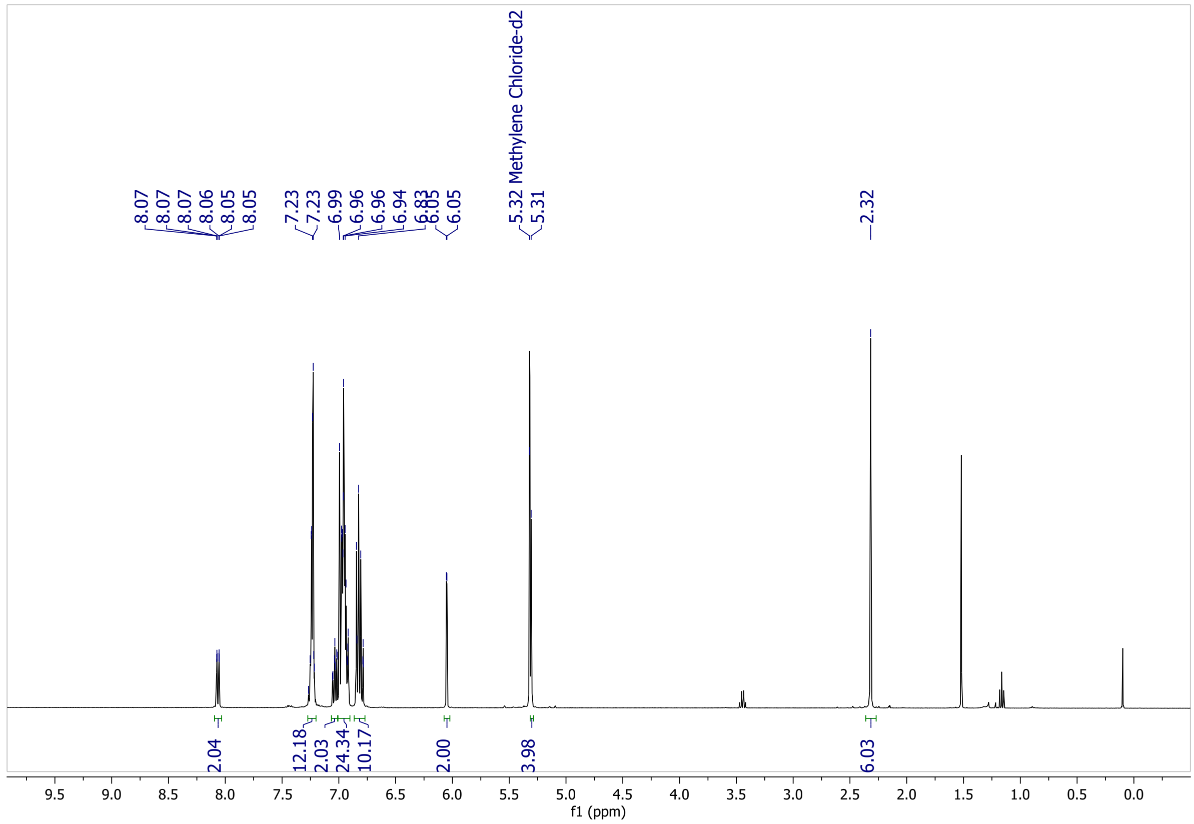

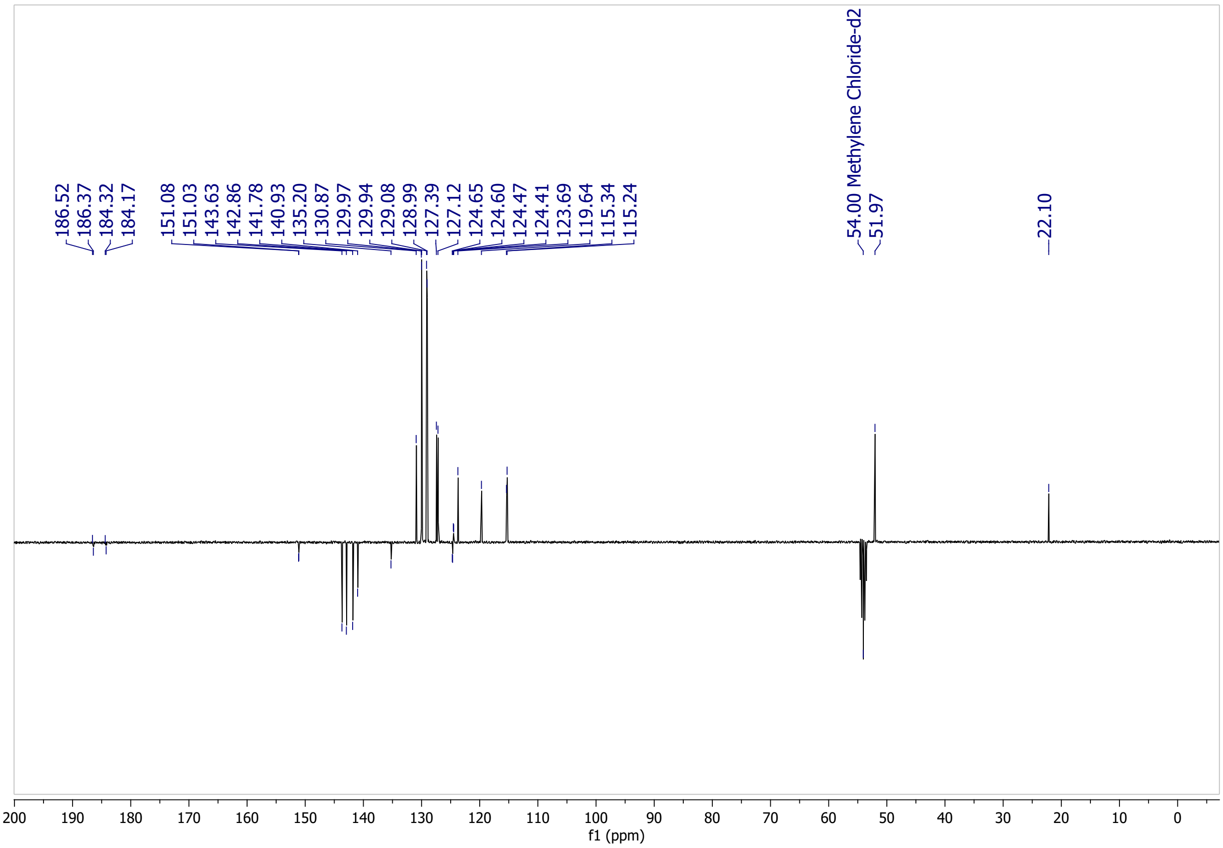

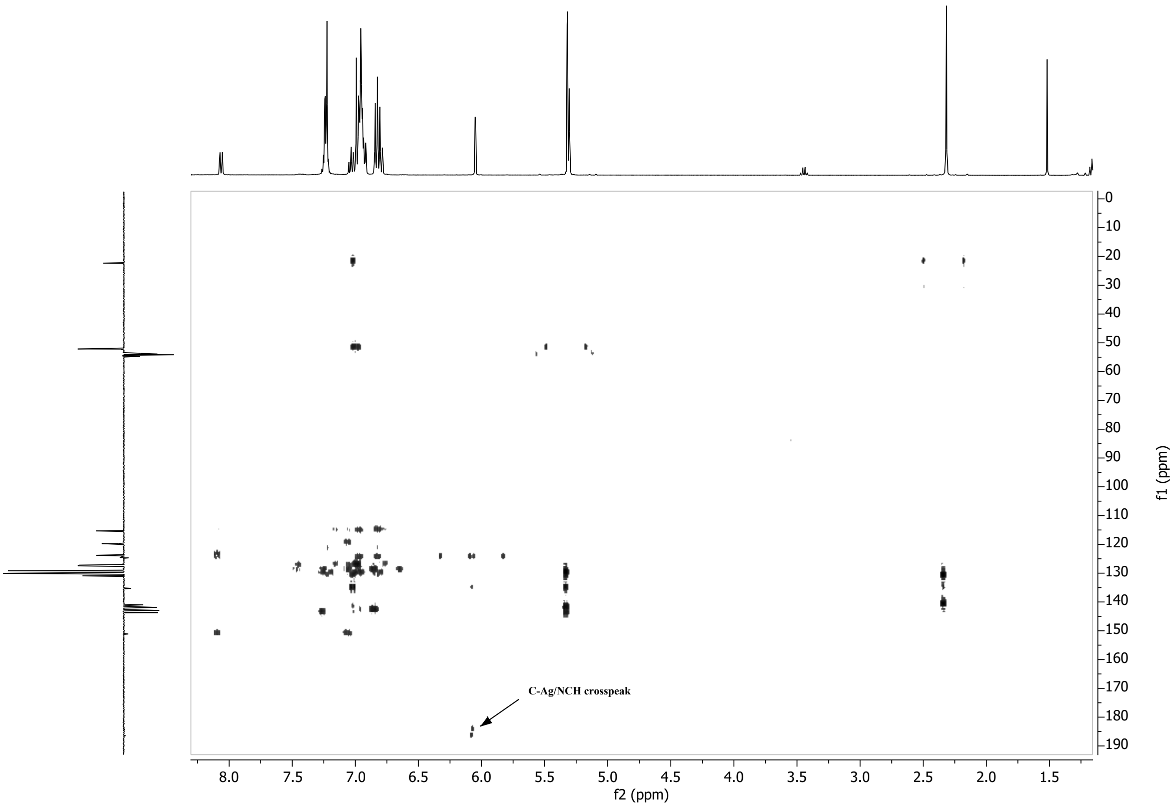
C-Ag/NCH crosspeak
f1 (ppm)
f2 (ppm)
0
10
20
30
40
50
60
70
80
90
100
110
120
130
140
150
160
170
180
190
8.0
7.5
7.0
6.5
6.0
5.5
5.0
4.5
4.0
3.5
3.0
2.5
2.0
1.5

^{1}H NMR and ^{13}C {^{1}H} NMR for [Cu(IPr)Cbz] (6a):

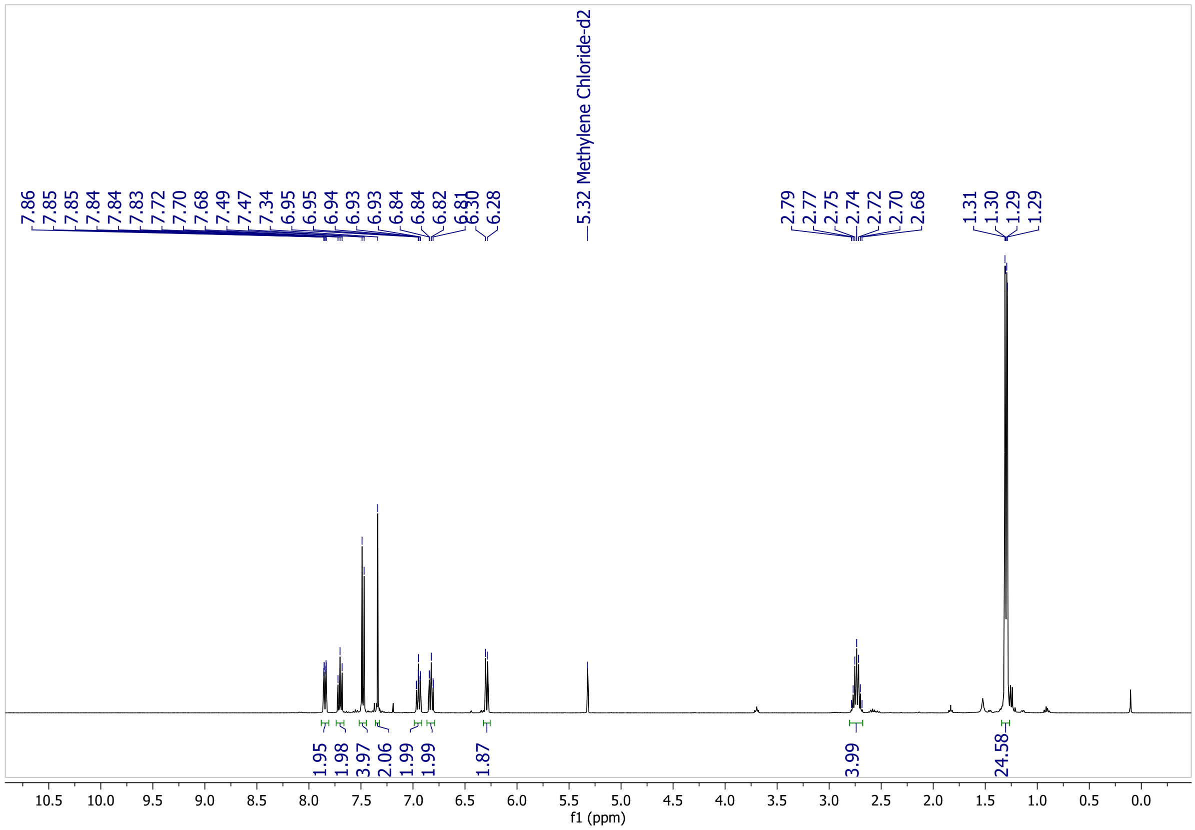

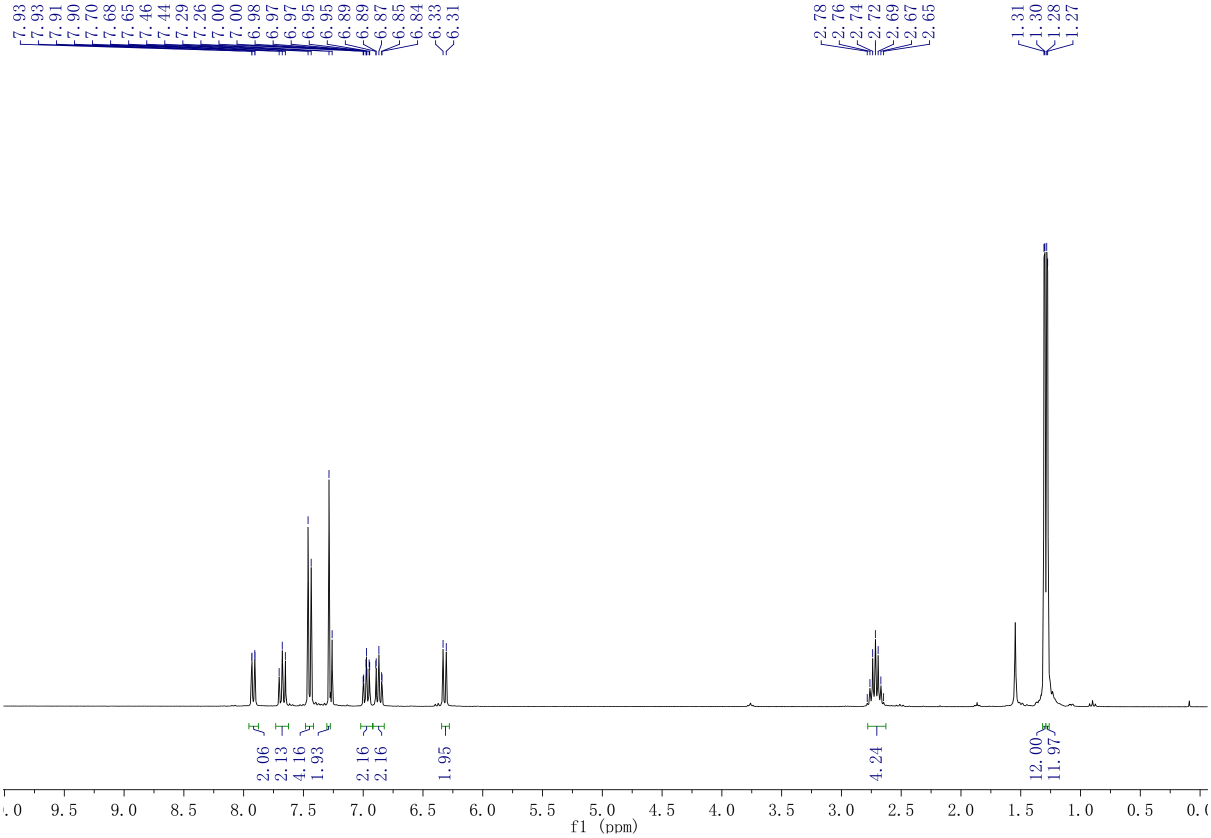

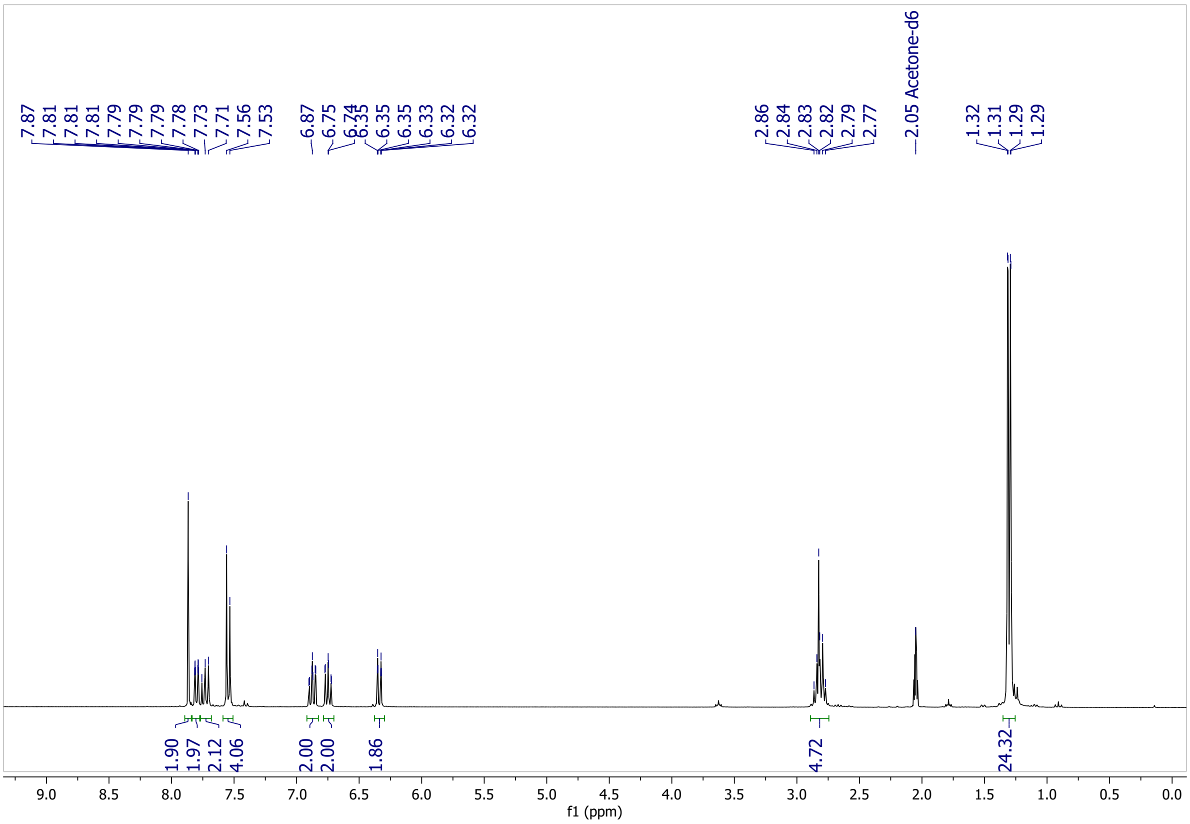
7.87
7.81
7.81
7.81
7.79
7.79
7.79
7.78
7.73
7.71
7.56
7.53
6.87
6.75
6.74
6.35
6.35
6.35
6.33
6.32
6.32
2.86
2.84
2.83
2.82
2.79
2.77
2.05 Acetone-d6
1.32
1.31
1.29
1.29
1.90
1.97
2.12
4.06
2.00
2.00
1.86
4.72
24.32
f1 (ppm)

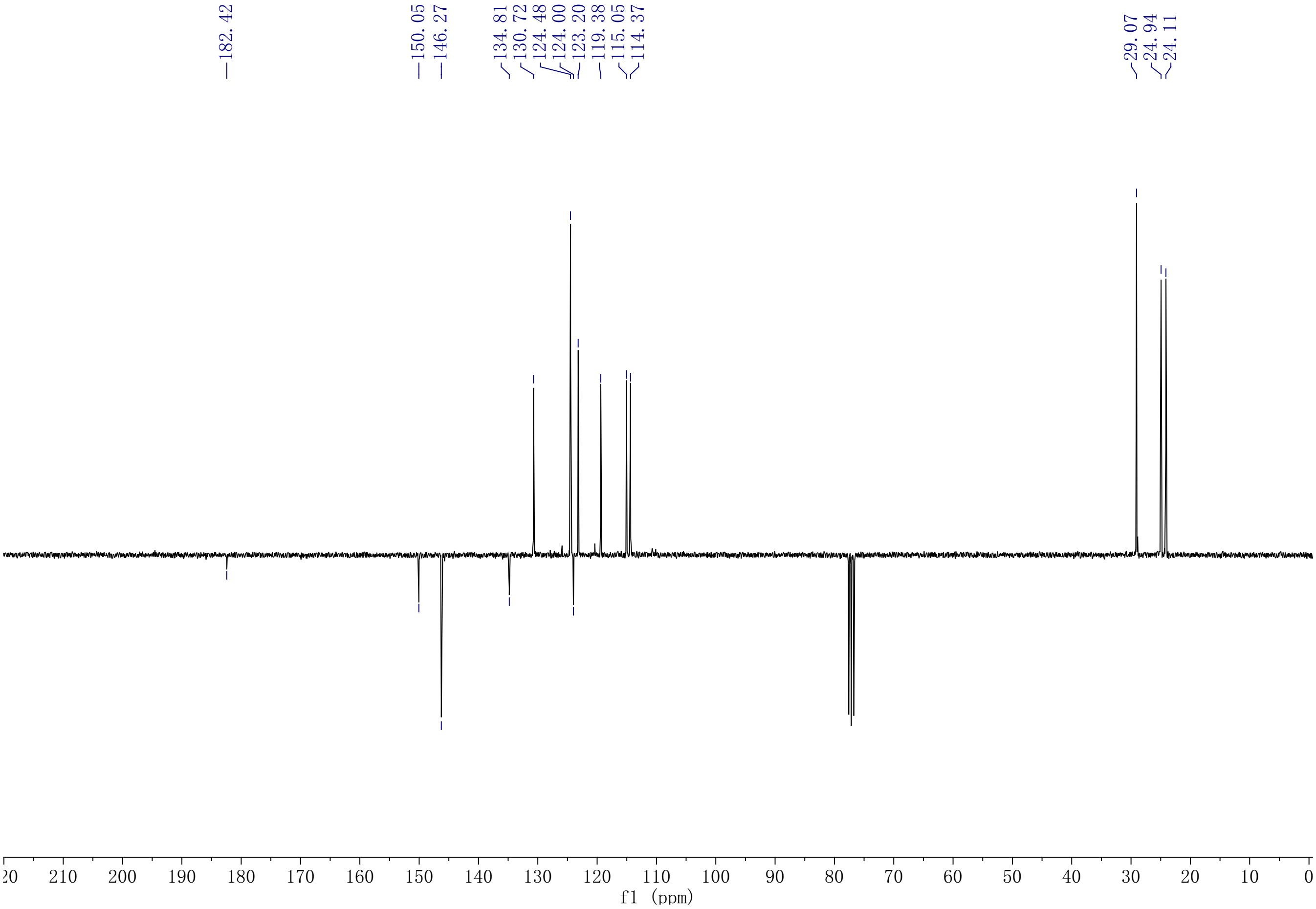
182.42
150.05
146.27
134.81
130.72
124.48
124.00
123.20
119.38
115.05
114.37
29.07
24.94
24.11
f1 (ppm)

^{1}H NMR, ^{13}C {^{1}H} NMR and 2D-HMBC [Cu(IMes)Cbz] (6b):

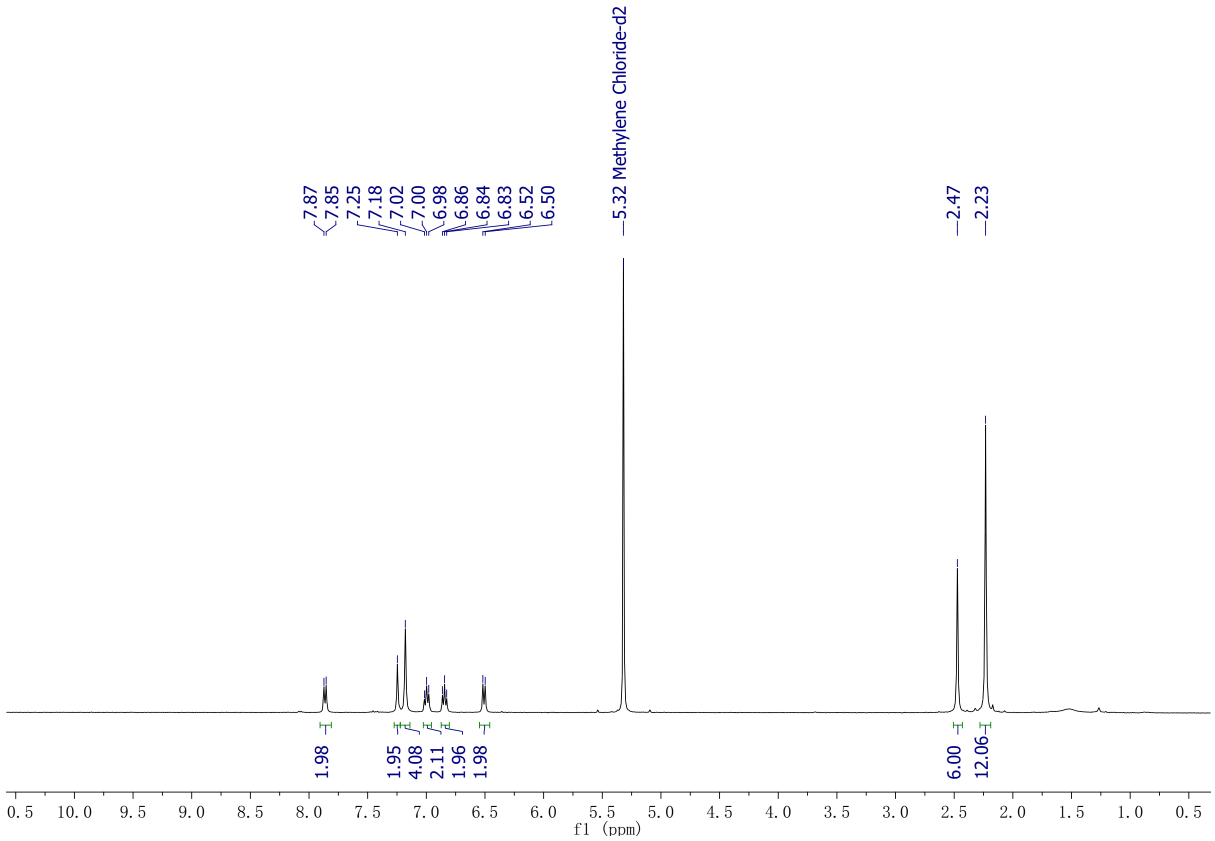

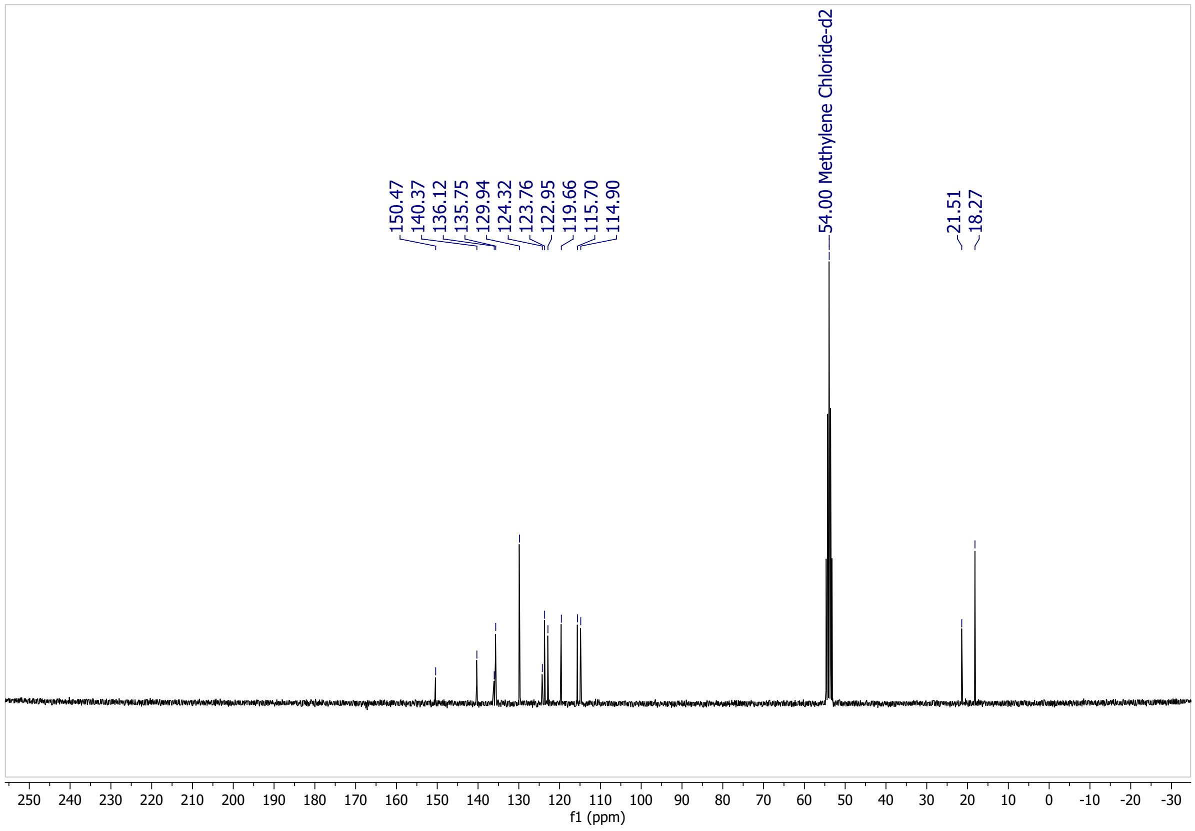

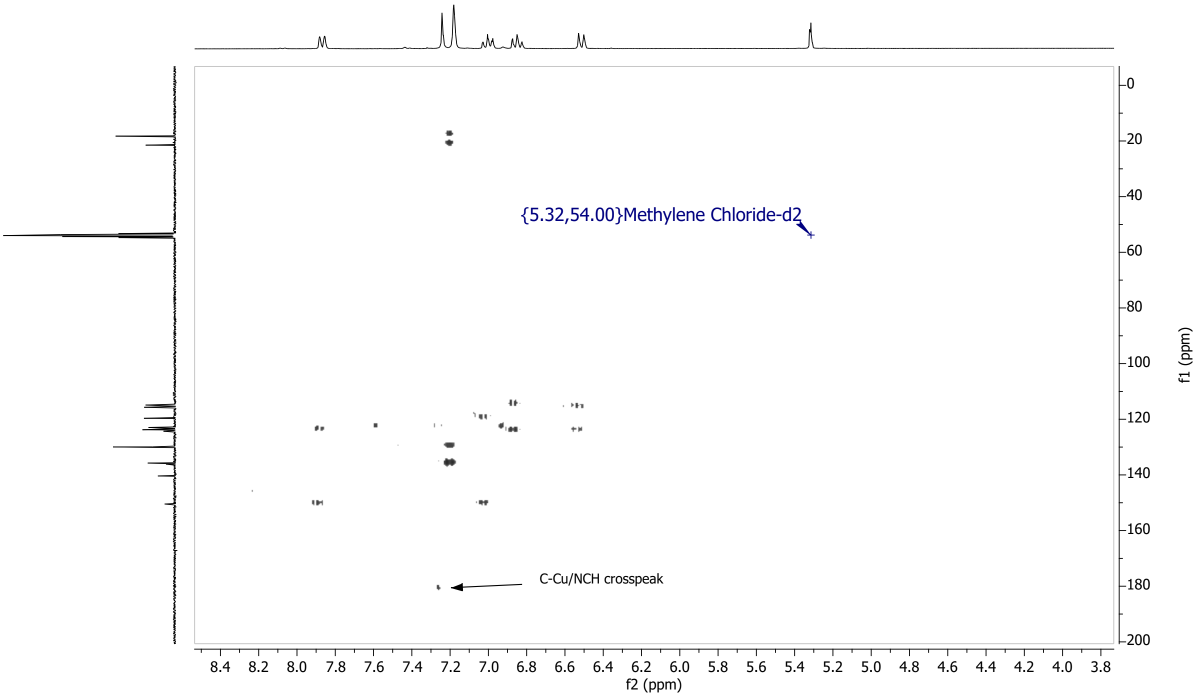
{5.32,54.00}Methylene Chloride-d2
C-Cu/NCH crosspeak
f1 (ppm)
f2 (ppm)
8.4 8.2 8.0 7.8 7.6 7.4 7.2 7.0 6.8 6.6 6.4 6.2 6.0 5.8 5.6 5.4 5.2 5.0 4.8 4.6 4.4 4.2 4.0 3.8
0 20 40 60 80 100 120 140 160 180 200

^{1}H NMR and ^{13}C {^{1}H} NMR (and APT^{13}C {^{1}H} NMR) for [Cu(CAAC^{Cy})(Cbz)] (6c):

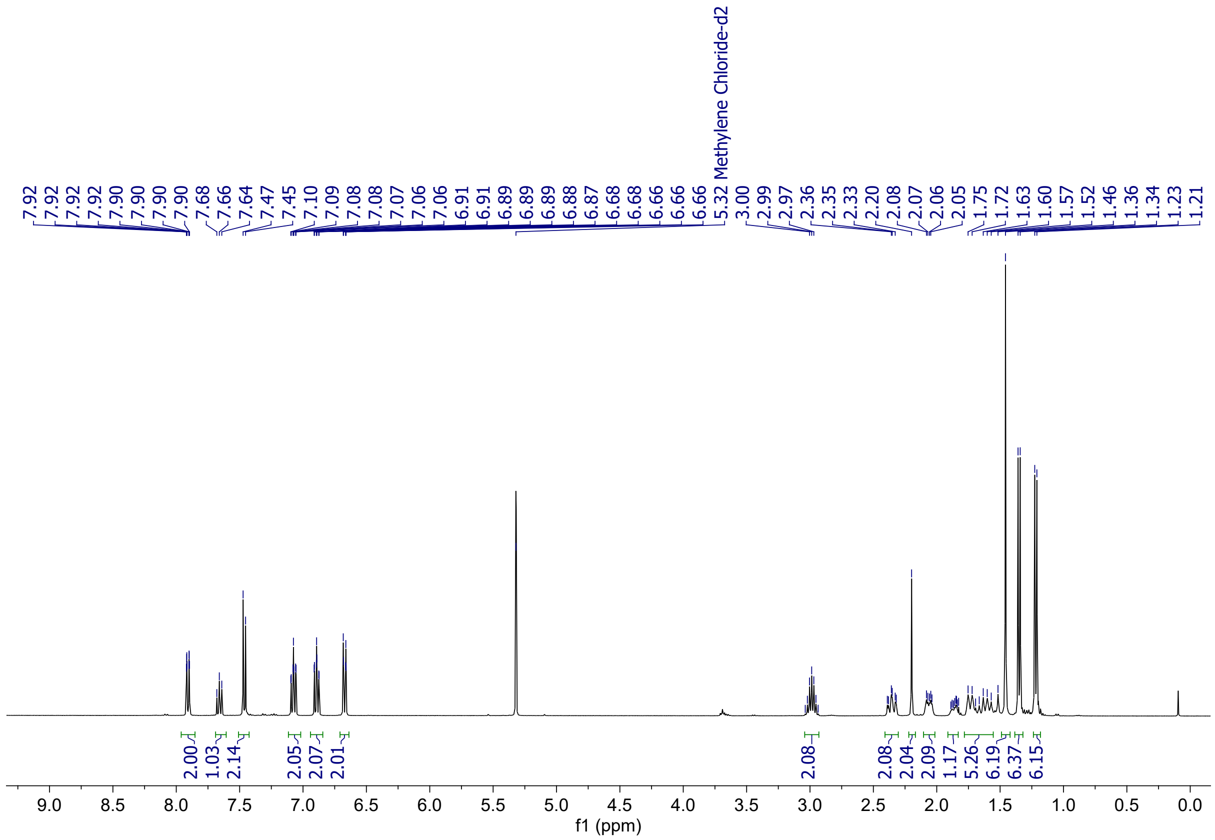

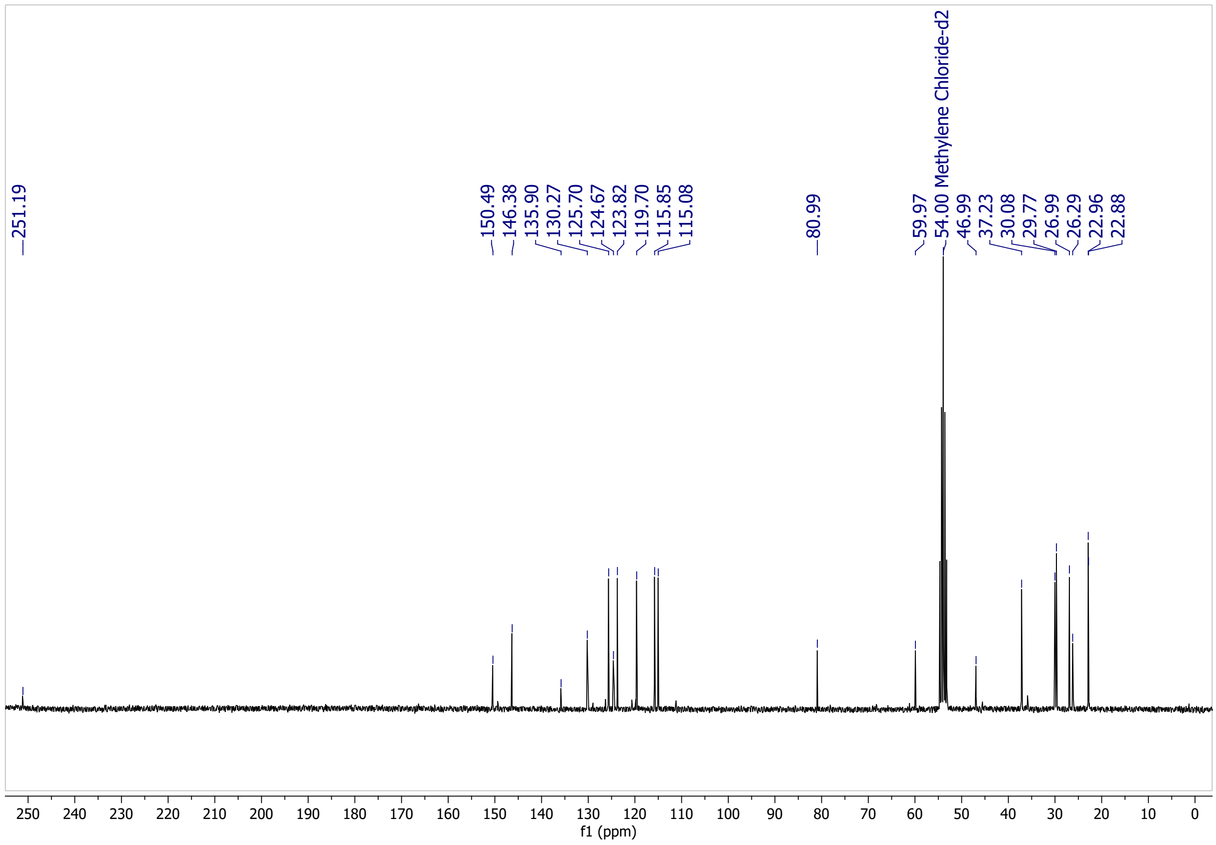

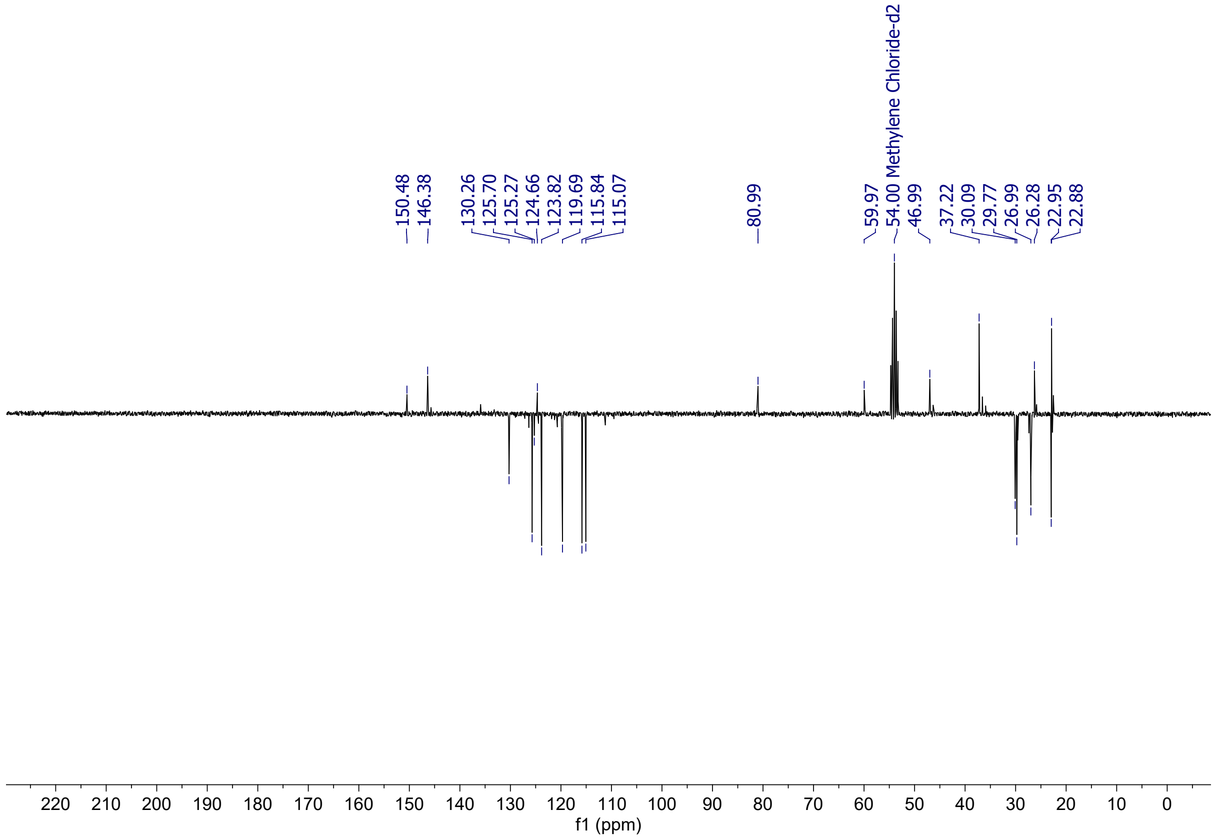
150.48
146.38
130.26
125.70
125.27
124.66
123.82
119.69
115.84
115.07
80.99
59.97
54.00 Methylene Chloride-d2
46.99
37.22
30.09
29.77
26.99
26.28
22.95
22.88
220 210 200 190 180 170 160 150 140 130 120 110 100 90 80 70 60 50 40 30 20 10 0
f1 (ppm)

^{1}H NMR and ^{13}C {^{1}H} NMR for [Au(IPr)(2-AmPy)] (4h):

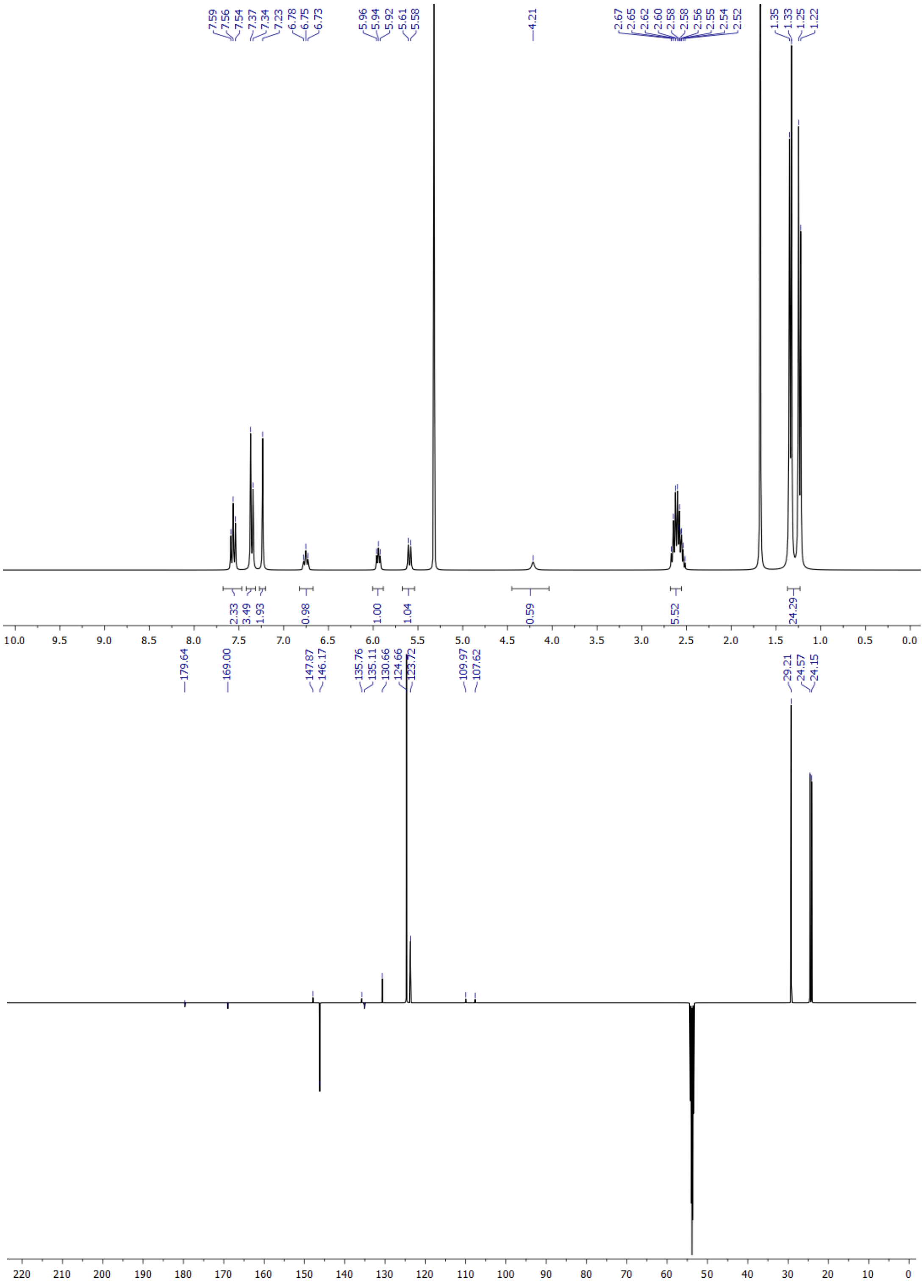

^{1}H NMR and ^{13}C {^{1}H} NMR for [Au(IPr)(3-AmIsoquin)] (4i):

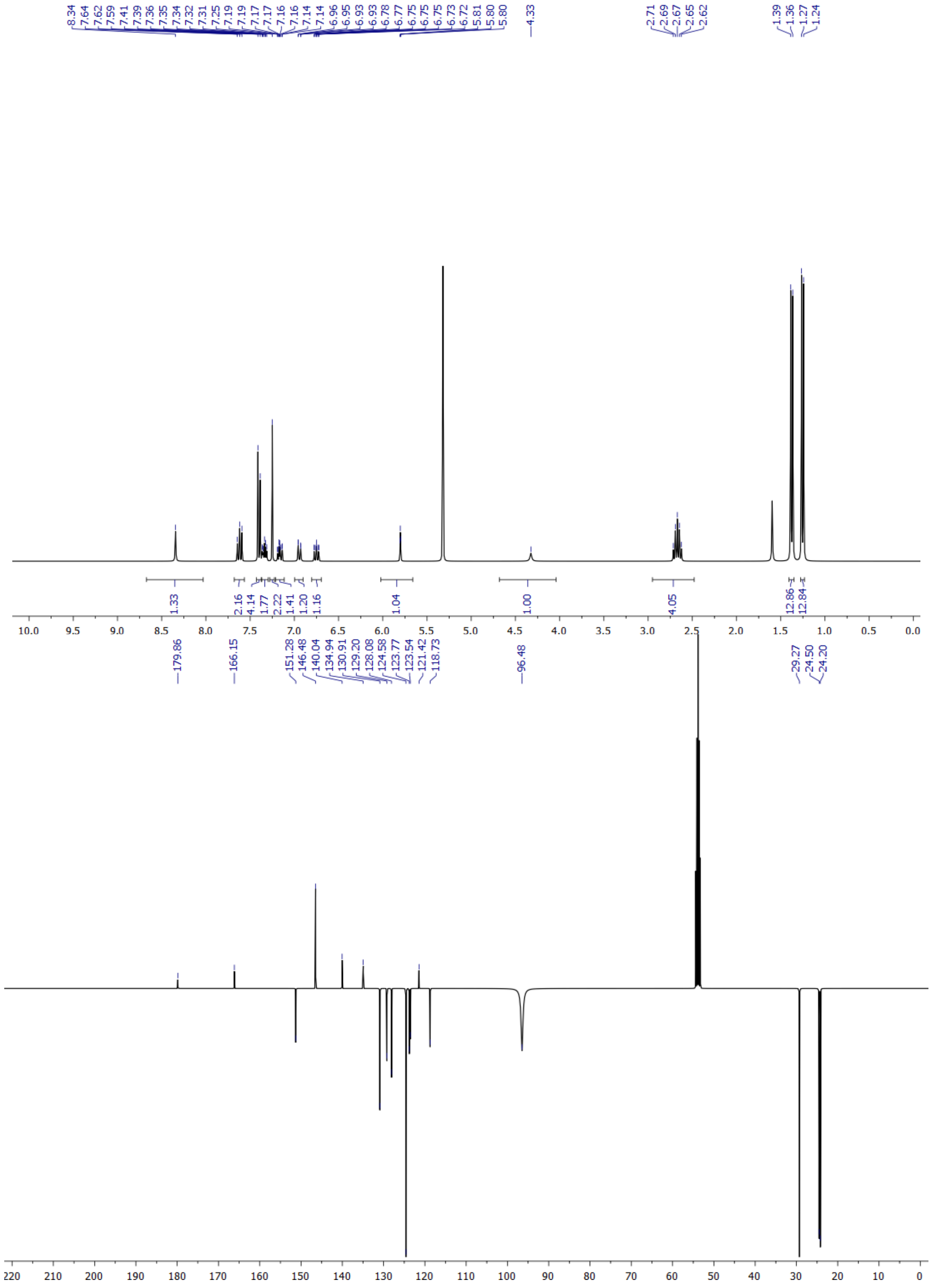

^{1}H NMR and ^{13}C {^{1}H} NMR for [Au(IPr)(NPh_2)] (4j):

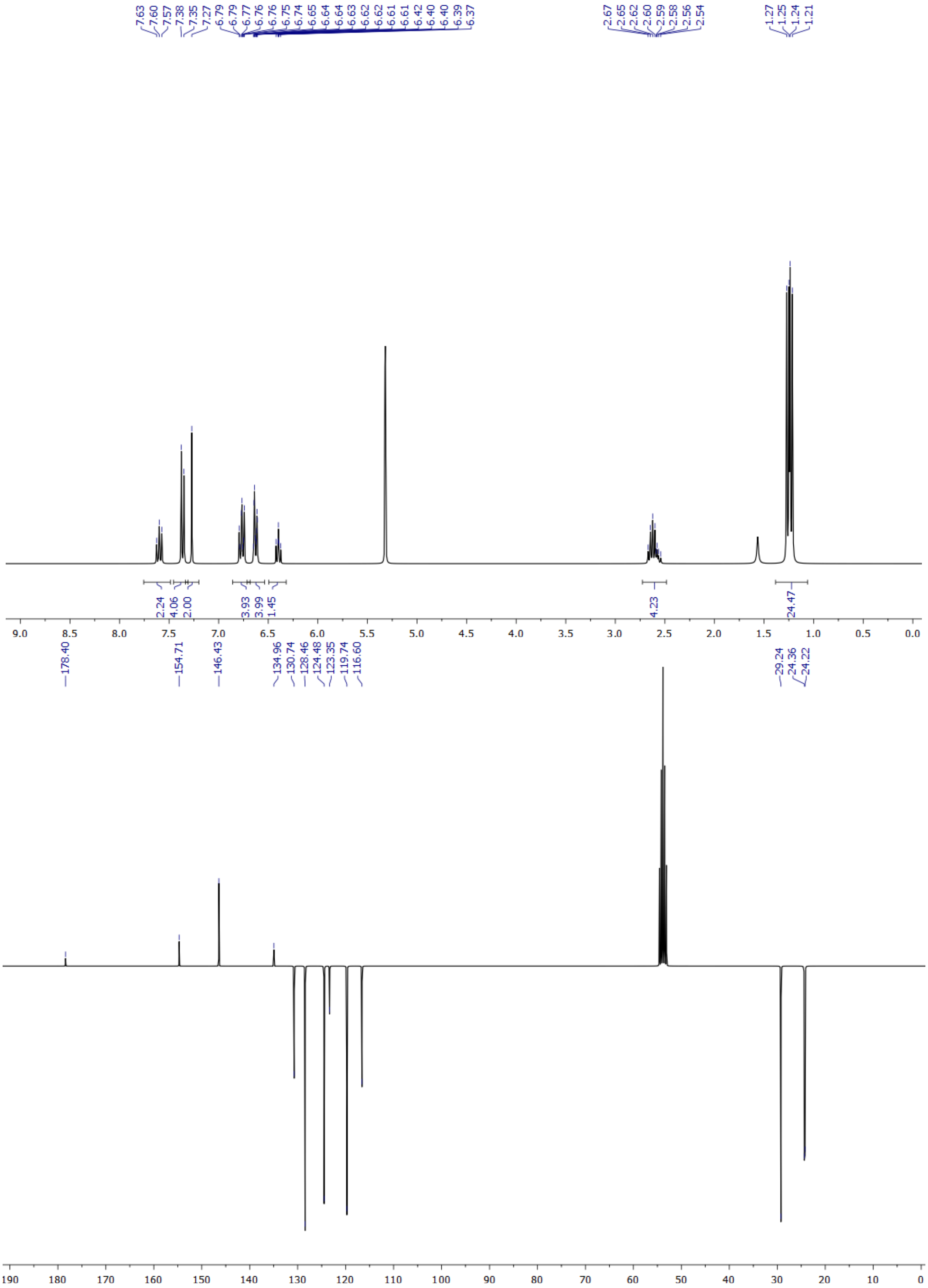

SUPPORTING INFORMATION

Cartesian Coordinates

IPr-Au-Cl

C	3.032115	1.258722	-0.679750
C	2.427354	0.020931	-0.948824
C	3.077780	-1.209176	-0.760880
C	4.395200	-1.172173	-0.297614
C	5.026480	0.038677	-0.032516
C	4.351247	1.239858	-0.219362
N	1.071349	0.010197	-1.406425
C	-0.000169	-0.000012	-0.578942
N	-1.071802	-0.010097	-1.406240
C	-0.679638	-0.007034	-2.730857
C	0.678937	0.007402	-2.730981
C	-2.427718	-0.020792	-0.948389
C	-3.078036	1.209338	-0.760242
C	-4.395396	1.172386	-0.296797
C	-5.026720	-0.038443	-0.031707
C	-4.351595	-1.239653	-0.218757
C	-3.032534	-1.258565	-0.679348
C	-2.372507	2.532499	-0.982212
C	-2.030671	3.185117	0.358671
C	-2.288688	-2.570428	-0.828622
C	-2.047384	-3.212545	0.538110
C	2.372309	-2.532362	-0.982869
C	3.172745	-3.471336	-1.882596
C	2.288124	2.570536	-0.828695
C	2.047093	3.212459	0.538179
C	2.030640	-3.185129	0.357978
C	3.001149	3.522529	-1.787421
C	-3.002027	-3.522253	-1.787283
C	-3.172948	3.471587	-1.881810
Cl	0.001284	-0.000292	3.701696
Au	0.000216	-0.000142	1.402196
H	-1.400130	-0.014170	-3.543274
H	1.399290	0.014714	-3.543519
H	-4.932010	2.108793	-0.128877
H	-6.055634	-0.045214	0.335480
H	-4.853438	-2.182859	0.009118
H	4.931895	-2.108557	-0.129829
H	6.055437	0.045488	0.334545
H	4.853061	2.183075	0.008529
H	-1.421268	2.322840	-1.495574
H	-1.301249	-2.349165	-1.261841
H	1.421022	-2.322728	-1.496153
H	1.300592	2.349249	-1.261688
H	-1.464906	4.116994	0.202783
H	-2.944328	3.432756	0.921600
H	-1.423673	2.513912	0.985716
H	-2.600670	4.390743	-2.080404
H	-3.411085	3.001114	-2.847970
H	-4.122431	3.773919	-1.413281
H	-1.444238	-4.127752	0.433136
H	-1.513631	-2.523894	1.211047
H	-2.997034	-3.488265	1.023042
H	-2.416497	-4.445211	-1.918920
H	-3.993487	-3.813097	-1.406320
H	-3.146374	-3.064241	-2.777759
H	2.600505	-4.390512	-2.081209
H	3.410781	-3.000781	-2.848741
H	4.122282	-3.773648	-1.414162
H	1.464856	-4.116988	0.202046
H	2.944375	-3.432840	0.920751
H	1.423726	-2.514019	0.985206
H	1.443717	4.127546	0.433482
H	1.513691	2.523615	1.211195
H	2.996824	3.488342	1.022862
H	2.415552	4.445488	-1.918757
H	3.992710	3.813346	-1.406699
H	3.145226	3.064663	-2.778004

IPr-Au-Cl CO_3-carbazole

C	-2.181746	3.421160	-1.431758
C	-0.847249	3.713059	-1.176781
C	0.142679	2.738688	-1.316363
C	-0.259155	1.444604	-1.709235
C	-1.587511	1.156769	-2.086304
C	-2.536567	2.168636	-1.909855
N	0.723508	0.399238	-1.712205
C	1.389932	-0.029870	-0.608843
N	2.220976	-0.999988	-1.055127
C	2.050424	-1.214960	-2.406797
C	1.111276	-0.330598	-2.818982
Cl	1.740597	1.659468	3.347284
C	3.270181	-1.582309	-0.276962
C	3.057049	-2.819246	0.352135
C	4.121845	-3.352136	1.088960
C	5.334955	-2.682631	1.196066
C	5.516749	-1.457268	0.561404
C	4.488561	-0.883748	-0.190427
C	1.726299	-3.522554	0.278956
C	1.844492	-5.015737	-0.006033
C	4.700987	0.453441	-0.873042
C	5.813740	0.377627	-1.917644
C	1.604080	3.130611	-1.175481
C	2.116366	3.680249	-2.508995
C	-1.982326	-0.139245	-2.763808
C	-1.933984	0.061372	-4.284189
C	0.922774	-3.255176	1.553183
C	4.946971	1.566124	0.144262
C	1.875642	4.094482	-0.026415
C	-3.349973	-0.670444	-2.360137
O	-0.771433	-2.029983	-0.851392
C	-1.256265	-3.198832	-0.865996
O	-2.415747	-3.396835	-0.175866
H	-2.751526	-2.545166	0.209786
H	2.584406	-1.996635	-2.936565
H	0.664110	-0.177510	-3.793180
H	3.979485	-4.311108	1.592380
H	1.130405	-3.088760	-0.540401
H	6.470689	-0.931181	0.656023
H	3.773788	0.703153	-1.408847
H	-0.567679	4.714554	-0.843590
H	2.179557	2.222401	-0.955052
H	-3.583328	1.947158	-2.122301
H	-1.270032	-0.916701	-2.435858
H	6.148260	-3.116032	1.785503
H	-0.072136	-3.714888	1.463676
H	1.432954	-3.663901	2.444030
H	0.770257	-2.175988	1.702720
H	0.836668	-5.371173	-0.269128
H	2.502614	-5.207793	-0.869883
H	2.235393	-5.586729	0.856005
H	5.030419	2.541725	-0.361910
H	4.116094	1.621268	0.865251
H	5.878291	1.396735	0.709086
H	5.917923	1.341007	-2.443136
H	6.786504	0.141188	-1.455780
H	5.601269	-0.400026	-2.667540
H	-2.952234	4.175236	-1.256336
H	2.959079	4.268876	0.070981
H	1.397797	5.075254	-0.182514
H	1.518720	3.675777	0.926782
H	3.191840	3.919037	-2.447758
H	1.973754	2.948127	-3.319130
H	1.575934	4.599361	-2.790615
H	-3.524516	-1.636547	-2.858519
H	-3.396728	-0.858179	-1.280039

H	-4.176126	0.003117	-2.645184
H	-2.081110	-0.900734	-4.801343
H	-2.735443	0.747012	-4.610188
H	-0.986255	0.500574	-4.638543
O	-0.771931	-4.198247	-1.424483
C	-7.966276	-0.841439	0.538284
C	-7.383992	-2.103315	0.287076
C	-6.012619	-2.301243	0.373282
C	-5.183659	-1.215134	0.724237
C	-5.776858	0.079544	0.964996
C	-7.163622	0.246811	0.873237
H	-9.051812	-0.719783	0.462191
H	-8.031442	-2.943853	0.013020
H	-5.552811	-3.271267	0.166075
H	-7.615528	1.228173	1.059029
C	-3.509303	0.077936	1.161536
C	-2.220878	0.613609	1.360936
C	-2.093981	1.956156	1.679577
C	-3.224051	2.798023	1.786800
C	-4.502194	2.294971	1.569229
C	-4.660680	0.937193	1.255152
H	-1.350003	-0.036269	1.238726
H	-1.094565	2.368580	1.846620
H	-3.085425	3.854828	2.036316
H	-5.376436	2.952332	1.644162
N	-3.834586	-1.205373	0.851604
Au	1.449363	0.704887	1.241691

IPr-Au-Cl CO_3-carbazole-TS

C	0.366426	5.022554	0.744782
C	0.342423	4.049100	1.735040
C	0.947706	2.804912	1.538887
C	1.542179	2.549556	0.285095
C	1.618313	3.536173	-0.720591
C	1.019458	4.771605	-0.454141
N	2.113939	1.256957	0.047043
C	1.441698	0.065921	0.039310
N	2.411987	-0.860352	-0.201076
C	3.651121	-0.270326	-0.352461
C	3.463055	1.059754	-0.193967
Cl	-1.850734	-1.293808	2.349273
C	2.207678	-2.268881	-0.072799
C	2.017393	-3.051169	-1.223690
C	1.822367	-4.424978	-1.038587
C	1.817037	-4.989755	0.231355
C	2.015827	-4.191195	1.352987
C	2.215568	-2.815201	1.225095
C	2.054716	-2.448098	-2.608173
C	3.446983	-2.605718	-3.222046
C	2.473486	-1.959410	2.449716
C	3.922543	-2.120928	2.914060
C	1.028223	1.817196	2.688671
C	2.381454	1.938893	3.391208
C	2.365594	3.349129	-2.028133
C	3.751315	3.998846	-1.944192
C	0.985061	-3.012588	-3.534002
C	1.479377	-2.217231	3.577080
C	-0.133615	1.910625	3.667637
C	1.608827	3.905896	-3.231094
O	-0.142659	1.755140	-2.219629
C	0.345650	0.705201	-2.682636
O	-0.465867	-0.394720	-2.724290
H	-1.234529	-0.214026	-2.122047
H	4.543849	-0.849202	-0.564824
H	4.162345	1.885498	-0.243456
H	1.655270	-5.055817	-1.914526
H	1.854905	-1.361411	-2.545899
H	1.998697	-4.638150	2.349751
H	2.339019	-0.912338	2.147431
H	-0.160277	4.251946	2.681541
H	0.959007	0.806739	2.260384
H	1.042680	5.541239	-1.228312
H	2.445501	2.265927	-2.229431
H	1.645043	-6.063443	0.350897
H	0.961071	-2.410751	-4.454333
H	1.168489	-4.067211	-3.811277
H	-0.007912	-2.915671	-3.075603
H	3.484734	-2.080631	-4.189496
H	4.228093	-2.168913	-2.580011
H	3.705330	-3.667638	-3.388091
H	1.663876	-1.512170	4.404643
H	0.441991	-2.068734	3.230508
H	1.578381	-3.236312	3.988247
H	4.130810	-1.456986	3.769354
H	4.124163	-3.158353	3.230723
H	4.631693	-1.870755	2.109495
H	-0.127593	5.984270	0.910549
H	-0.092951	1.063655	4.368656
H	-0.116823	2.842396	4.259481
H	-1.092856	1.836411	3.134861
H	2.468260	1.187605	4.193905
H	3.214003	1.778433	2.688102
H	2.507660	2.938722	3.841780
H	2.130647	3.610131	-4.155232
H	0.608005	3.449921	-3.235791
H	1.542910	5.008988	-3.221329
H	4.330390	3.788961	-2.858921
H	3.663704	5.095128	-1.845472
H	4.345549	3.653197	-1.083154
O	1.515934	0.527152	-3.104541
C	-5.670333	-2.870440	-0.769482
C	-4.381229	-3.362494	-1.062081
C	-3.272945	-2.526847	-1.085631
C	-3.447302	-1.156836	-0.817774
C	-4.757332	-0.654961	-0.507879
C	-5.857329	-1.519463	-0.489312
H	-6.522942	-3.557219	-0.754927
H	-4.250602	-4.430650	-1.266305
H	-2.271348	-2.912500	-1.292597
H	-6.856482	-1.137261	-0.251519
C	-3.165421	0.975209	-0.477844
C	-2.627150	2.268285	-0.335353
C	-3.478408	3.300536	0.032736
C	-4.856764	3.084981	0.252103
C	-5.402734	1.815154	0.094113
C	-4.566580	0.751598	-0.270686
H	-1.570107	2.424961	-0.562383
H	-3.067862	4.309543	0.147172
H	-5.497814	3.923852	0.543407
H	-6.472894	1.646889	0.259991
N	-2.500415	-0.176043	-0.805120
Au	-0.411323	-0.423869	0.589674

IPr-Au-carbazole

C	3.049068	1.928555	1.279286
C	2.898773	1.307015	0.028677
C	2.878119	2.016083	-1.182655
C	3.037166	3.403326	-1.115305
C	3.200763	4.046669	0.106338
C	3.204012	3.316903	1.290634
N	2.699478	-0.110100	-0.004059
C	1.472446	-0.677594	-0.008865
N	1.704485	-2.010560	-0.025474
C	3.059984	-2.277746	-0.029628
C	3.690835	-1.073121	-0.018444
C	0.645023	-2.974067	-0.049168
C	0.189240	-3.431479	-1.296521
C	-0.882422	-4.327764	-1.298728
C	-1.473384	-4.739251	-0.108612
C	-1.006914	-4.259300	1.110124
C	0.062794	-3.361377	1.168373
C	0.770400	-2.921471	-2.599968

C	-0.195517	-1.933346	-3.257069
C	0.524389	-2.808105	2.501314
C	1.014981	-3.916570	3.431335
Au	-0.305394	0.201026	0.002145
N	-2.144858	1.019653	0.010686
C	2.651416	1.336442	-2.517886
C	3.821668	1.565930	-3.473309
C	2.966638	1.149350	2.576870
C	4.126280	1.460871	3.519990
C	1.321235	1.776268	-3.130777
C	1.611420	1.383662	3.247805
C	-0.575414	-1.965701	3.148398
C	1.154791	-4.054815	-3.548210
H	4.745205	-0.813177	-0.017909
H	3.447231	-3.292217	-0.041800
H	3.313909	3.837264	2.244397
H	3.316753	5.132523	0.137368
H	3.020362	3.989581	-2.036908
H	-1.272792	-4.695293	-2.250128
H	-2.317250	-5.432574	-0.132552
H	-1.493068	-4.574316	2.036095
H	3.027782	0.078124	2.329746
H	2.583960	0.252780	-2.337163
H	1.693112	-2.368896	-2.363545
H	1.377587	-2.139423	2.309171
H	1.518622	0.771510	4.158417
H	1.489561	2.439917	3.534654
H	0.782280	1.123424	2.571906
H	4.070428	0.824424	4.416319
H	5.099460	1.286401	3.036352
H	4.104451	2.507040	3.862564
H	1.128305	1.231349	-4.067689
H	0.483412	1.581510	-2.443385
H	1.322428	2.852899	-3.361906
H	3.663042	1.016758	-4.414149
H	3.932502	2.631573	-3.728091
H	4.772613	1.226584	-3.034986
H	1.641104	-3.650568	-4.449154
H	1.850439	-4.762233	-3.071695
H	0.273672	-4.624479	-3.882038
H	0.245080	-1.509995	-4.173218
H	-1.141013	-2.426930	-3.531269
H	-0.438926	-1.103653	-2.575305
H	-0.213501	-1.509851	4.082790
H	-0.902657	-1.157608	2.476006
H	-1.459369	-2.575599	3.391333
H	1.396373	-3.489099	4.371452
H	0.203419	-4.613998	3.691370
H	1.823566	-4.502596	2.968398
C	-2.470964	2.354396	-0.045730
C	-3.885575	2.526313	-0.017383
C	-3.323477	0.312198	0.075063
C	-3.507213	-1.076034	0.146101
C	-4.805956	-1.565651	0.207449
C	-5.915882	-0.701067	0.197812
C	-5.738366	0.676259	0.124958
C	-4.440600	1.196241	0.062935
H	-2.645926	-1.750564	0.151008
H	-4.967515	-2.645698	0.263226
H	-6.924626	-1.117932	0.246948
H	-6.602860	1.345606	0.116449
C	-4.430590	3.814054	-0.068029
C	-3.578052	4.909809	-0.145814
C	-2.182966	4.729847	-0.172910
C	-1.615869	3.462339	-0.123541
H	-5.514436	3.956521	-0.047033
H	-3.991909	5.920153	-0.186223
H	-1.531167	5.605601	-0.233725
H	-0.530971	3.322723	-0.143046

IPr-Au-Cl HCO_3-carbazole

C	3.172782	-2.192028	1.768319
C	2.982384	-0.888577	2.212344
C	1.701408	-0.337378	2.271467
C	0.633627	-1.157385	1.858350
C	0.794025	-2.473750	1.390688
C	2.100529	-2.968307	1.352357
N	-0.692434	-0.613102	1.952541
C	-1.440657	-0.174377	0.915219
N	-2.597670	0.252471	1.482817
C	-2.573560	0.092028	2.854400
C	-1.367325	-0.456267	3.148315
Au	-1.125770	-0.069621	-1.042332
Cl	-1.090960	0.176126	-3.349110
C	-3.697984	0.771853	0.734586
C	-4.672843	-0.128281	0.274130
C	-5.734890	0.399098	-0.464461
C	-5.810996	1.761715	-0.734247
C	-4.820197	2.625099	-0.279445
C	-3.736157	2.148307	0.462101
C	-4.552143	-1.621778	0.502477
C	-5.808651	-2.211923	1.138555
C	-2.623852	3.082574	0.893055
C	-3.142027	4.219211	1.771929
C	1.465759	1.090228	2.740184
C	2.622245	1.650082	3.561285
C	-0.382027	-3.322373	0.927859
C	-1.308518	-3.713340	2.084011
C	-4.182614	-2.336613	-0.798533
C	-1.853749	3.604744	-0.320838
C	1.154577	2.031466	1.575330
C	0.042482	-4.572053	0.165452
H	2.980414	-0.477343	-2.049678
H	-3.412640	0.375069	3.482228
H	-0.927583	-0.764436	4.091957
H	-6.505716	-0.272588	-0.849477
H	-3.723282	-1.787096	1.206840
H	-4.877072	3.689322	-0.520207
H	-1.913185	2.499982	1.497262
H	3.852144	-0.290284	2.485764
H	0.580679	1.076112	3.400100
H	2.285589	-3.958936	0.943252
H	-0.954892	-2.699277	0.219381
H	-6.645188	2.153300	-1.321672
H	-4.013580	-3.409243	-0.615231
H	-4.983827	-2.239160	-1.548581
H	-3.262944	-1.917001	-1.234743
H	-5.660524	-3.281800	1.352503
H	-6.058970	-1.705112	2.083508
H	-6.681486	-2.128405	0.471512
H	-0.983812	4.198266	-0.000639
H	-1.483345	2.773835	-0.940650
H	-2.491185	4.241586	-0.955389
H	-2.306046	4.850547	2.111130
H	-3.844892	4.867545	1.224571
H	-3.664521	3.835122	2.661922
H	4.185491	-2.592015	1.709472
H	0.858273	3.024374	1.950041
H	2.040466	2.161972	0.941182
H	0.353011	1.653444	0.927297
H	2.351589	2.638824	3.963086
H	2.885768	0.994924	4.406098
H	3.520584	1.786790	2.939444
H	-0.843742	-5.038709	-0.291656
H	0.761870	-4.329407	-0.632435
H	0.494579	-5.315615	0.844690
H	-2.149753	-4.314665	1.702999
H	-0.765452	-4.328275	2.820611
H	-1.734488	-2.851416	2.616357
C	2.986949	4.279861	-0.746884
C	2.062719	3.635864	-1.594705
C	2.169721	2.285177	-1.899874

C	3.235585	1.571426	-1.331102
C	4.176228	2.206923	-0.462704
C	4.042158	3.572744	-0.180748
H	2.868182	5.345007	-0.531064
H	1.236279	4.211465	-2.021075
H	1.446087	1.778192	-2.543128
H	4.752767	4.073416	0.483346
C	4.642728	-0.018943	-0.704641
C	5.294957	-1.246377	-0.525356
C	6.397518	-1.271111	0.318349
C	6.847572	-0.111098	0.982308
C	6.199416	1.106799	0.802561
C	5.089455	1.167976	-0.049840
H	4.894458	-2.141314	-1.008984
H	6.923448	-2.216296	0.482480
H	7.716378	-0.172215	1.643176
H	6.552272	2.004318	1.319180
N	3.541418	0.249371	-1.459294
O	0.402899	-2.745699	-2.553991
C	1.726879	-2.438705	-2.317324
O	2.103057	-1.346156	-2.823406
O	2.372321	-3.247728	-1.649140
H	0.043483	-1.976460	-3.027414

IPr-Au-Cl HCO_3-carbazole-TS

C	3.919232	2.782463	-1.253763
C	3.554232	1.879504	-2.244046
C	2.223416	1.488530	-2.399128
C	1.274616	2.074251	-1.539453
C	1.608152	3.004195	-0.536074
C	2.963045	3.327408	-0.405757
N	-0.095912	1.701679	-1.737174
C	-0.811831	0.860448	-0.949090
N	-2.002419	0.728770	-1.591533
C	-2.037928	1.478217	-2.750910
C	-0.833219	2.094202	-2.842032
Au	-0.495476	0.100268	0.857514
Cl	-1.228909	-0.164112	3.166860
C	-3.088127	-0.047281	-1.080077
C	-3.993642	0.573401	-0.203543
C	-5.045831	-0.202828	0.286376
C	-5.173445	-1.541424	-0.070743
C	-4.247028	-2.133689	-0.921538
C	-3.179722	-1.398875	-1.445388
C	-3.839797	2.022989	0.209030
C	-4.888658	2.898733	-0.477320
C	-2.137141	-2.067542	-2.317171
C	-2.758544	-2.729779	-3.545432
C	1.823662	0.428578	-3.412454
C	2.824145	0.270824	-4.551799
C	0.563599	3.633290	0.373643
C	-0.415555	4.523980	-0.397861
C	-3.855956	2.190101	1.726550
C	-1.302984	-3.051649	-1.498683
C	1.582060	-0.921919	-2.733241
C	1.167183	4.423984	1.528352
H	1.943160	-0.600138	2.668067
H	-2.912016	1.503028	-3.394179
H	-0.429430	2.779128	-3.581457
H	-5.763944	0.244838	0.977150
H	-2.850836	2.359906	-0.133837
H	-4.339499	-3.193288	-1.170864
H	-1.454135	-1.285036	-2.679736
H	4.321066	1.442371	-2.883374
H	0.870725	0.746667	-3.866476
H	3.276749	4.004004	0.387611
H	-0.005732	2.798428	0.819819
H	-5.995522	-2.135864	0.335747
H	-3.627295	3.234208	1.992871
H	-4.843365	1.949476	2.152940
H	-3.104804	1.540614	2.202718
H	-4.743008	3.957816	-0.211408
H	-4.832739	2.812122	-1.573874
H	-5.907837	2.611027	-0.171419
H	-0.495216	-3.482747	-2.108679
H	-0.836531	-2.562262	-0.631763
H	-1.915606	-3.881060	-1.112761
H	-1.971601	-3.159150	-4.184941
H	-3.436688	-3.551120	-3.263923
H	-3.335455	-2.009649	-4.147062
H	4.970390	3.051432	-1.124037
H	1.167262	-1.643756	-3.455162
H	2.522297	-1.331285	-2.339114
H	0.888797	-0.846037	-1.885076
H	2.418881	-0.416806	-5.309959
H	3.050315	1.229767	-5.044127
H	3.769285	-0.166979	-4.194495
H	0.364483	4.729470	2.215867
H	1.883904	3.828615	2.110936
H	1.663268	5.341643	1.167840
H	-1.151399	4.956849	0.298081
H	0.118266	5.357893	-0.883360
H	-0.977448	3.985689	-1.173198
C	0.159096	-5.445656	1.027789
C	-0.531121	-4.599154	1.917084
C	-0.141992	-3.282314	2.125817
C	0.968488	-2.785181	1.419321
C	1.680714	-3.650145	0.528262
C	1.268820	-4.974114	0.336136
H	-0.176700	-6.477035	0.888121
H	-1.398697	-4.986909	2.459491
H	-0.688007	-2.627652	2.806405
H	1.815203	-5.628045	-0.350768
C	2.587666	-1.559534	0.592073
C	3.508455	-0.537801	0.301433
C	4.566242	-0.819758	-0.553362
C	4.727942	-2.088709	-1.143636
C	3.820590	-3.107125	-0.869759
C	2.753140	-2.851450	-0.001684
H	3.408338	0.452007	0.750885
H	5.293693	-0.033016	-0.769410
H	5.574701	-2.272721	-1.810781
H	3.943604	-4.097564	-1.318723
N	1.506298	-1.514955	1.442280
O	1.126642	1.735247	3.155272
C	2.387826	1.281067	3.174817
O	2.537127	-0.019513	3.293599
O	3.304095	2.067895	3.106943
H	0.465714	1.020967	3.301242

IPr-Au-Cl NMe_3-carbazole

C	-2.400656	3.264408	1.613384
C	-2.463889	1.991798	2.175089
C	-1.318205	1.203473	2.298678
C	-0.111620	1.747668	1.817567
C	-0.013274	3.029320	1.256095
C	-1.190698	3.779948	1.166845
N	1.078118	0.956958	1.922566
C	1.638318	0.278568	0.892838
N	2.725604	-0.323941	1.426690
C	2.851911	-0.026160	2.769323
C	1.811097	0.788532	3.081835
Au	1.089462	0.193438	-1.014116
Cl	0.565338	0.107373	-3.271702
C	3.631298	-1.141530	0.677396
C	4.748963	-0.527486	0.089047
C	5.613209	-1.341684	-0.646842
C	5.365513	-2.703258	-0.787847
C	4.243921	-3.278846	-0.201257
C	3.346511	-2.509763	0.544662
C	4.989779	0.966228	0.185146
C	6.395265	1.293407	0.684875

C	2.096953	-3.136153	1.129125
C	2.420789	-4.313373	2.046891
C	-1.369231	-0.184612	2.913304
C	-2.583815	-0.401458	3.809532
C	1.301271	3.615822	0.779883
C	1.696174	4.828450	1.622859
C	4.690619	1.643580	-1.153500
C	1.124703	-3.534258	0.017801
C	-1.307202	-1.274719	1.843370
C	1.264482	3.948422	-0.710647
H	-3.306843	0.466399	-1.464375
H	3.667098	-0.417815	3.370135
H	1.525684	1.265434	4.014831
H	6.488557	-0.899481	-1.127766
H	4.280491	1.374043	0.921917
H	4.052284	-4.345964	-0.334063
H	1.594434	-2.373915	1.743400
H	-3.422810	1.603618	2.517675
H	-0.471517	-0.288202	3.546829
H	-1.154403	4.784647	0.739004
H	2.080747	2.851882	0.918480
H	6.050960	-3.321956	-1.371782
H	4.806113	2.735640	-1.068971
H	5.376900	1.289958	-1.938983
H	3.663367	1.429410	-1.487025
H	6.513208	2.380479	0.812374
H	6.602558	0.810977	1.652225
H	7.167697	0.962310	-0.026564
H	0.180293	-3.913216	0.436440
H	0.879840	-2.677321	-0.627983
H	1.552778	-4.322304	-0.622107
H	1.498535	-4.704494	2.503163
H	2.890107	-5.142390	1.494359
H	3.106348	-4.019518	2.856477
H	-3.311227	3.863137	1.529483
H	-1.246985	-2.269704	2.310191
H	-2.211868	-1.254497	1.225654
H	-0.450611	-1.160325	1.166780
H	-2.497884	-1.371668	4.321063
H	-2.676522	0.381259	4.578004
H	-3.517996	-0.430057	3.226823
H	2.244209	4.324025	-1.043778
H	1.023036	3.056421	-1.308201
H	0.515518	4.723982	-0.935915
H	2.678241	5.213688	1.307410
H	0.967492	5.647403	1.515551
H	1.755538	4.573309	2.691947
C	-2.483071	-4.361394	-1.229387
C	-1.832989	-3.447545	-2.078821
C	-2.134662	-2.091615	-2.060176
C	-3.118871	-1.659668	-1.165103
C	-3.782100	-2.568077	-0.296017
C	-3.454543	-3.928634	-0.334996
H	-2.217711	-5.420258	-1.273843
H	-1.061797	-3.805950	-2.765204
H	-1.595713	-1.389012	-2.698391
H	-3.954977	-4.637445	0.329692
C	-4.520283	-0.427658	0.070893
C	-5.253843	0.612291	0.653699
C	-6.130762	0.296877	1.685887
C	-6.287160	-1.025545	2.136988
C	-5.569897	-2.061884	1.549365
C	-4.682057	-1.773522	0.507475
H	-5.134749	1.641158	0.310758
H	-6.712195	1.094883	2.154799
H	-6.983495	-1.237997	2.951169
H	-5.696444	-3.090266	1.897017
N	-3.577981	-0.384531	-0.925043
N	-3.125838	1.901698	-2.589217
C	-1.948463	2.700707	-2.316037
H	-1.886018	3.595294	-2.972776
H	-1.958342	3.037113	-1.269503
H	-1.047160	2.093349	-2.476126
C	-3.067874	1.281055	-3.898204
H	-2.125428	0.723701	-3.998922
H	-3.908068	0.579827	-4.019985
H	-3.117131	2.027253	-4.719564
C	-4.345253	2.653870	-2.402479
H	-5.220993	1.994747	-2.508777
H	-4.364526	3.091435	-1.392351
H	-4.448295	3.485275	-3.132794

IPr-Au-Cl NMe3-carbazole-TS

C	-1.872546	4.625337	-0.046605
C	-1.721867	4.086360	1.227799
C	-0.623030	3.284916	1.544335
C	0.321958	3.057805	0.527623
C	0.203414	3.593638	-0.764172
C	-0.922353	4.380145	-1.031162
N	1.434070	2.207000	0.814585
C	1.453774	0.890004	0.493519
N	2.639404	0.438685	0.967700
C	3.352364	1.454101	1.576266
C	2.593483	2.576256	1.474061
Au	0.068059	-0.157731	-0.457679
Cl	0.651617	-1.443722	-2.720736
C	3.053768	-0.924413	0.830316
C	3.857772	-1.268867	-0.268755
C	4.212954	-2.612391	-0.403261
C	3.770315	-3.564487	0.509104
C	2.961841	-3.194236	1.577679
C	2.581968	-1.862150	1.762557
C	4.339472	-0.228669	-1.260290
C	5.767097	0.208615	-0.923528
C	1.696134	-1.473380	2.929416
C	2.418886	-1.682748	4.260162
C	-0.491955	2.634724	2.908649
C	-0.940845	3.546458	4.046743
C	1.240420	3.350896	-1.843012
C	1.862119	4.666353	-2.313065
C	4.222950	-0.693961	-2.708698
C	0.357221	-2.206328	2.888556
C	-1.236866	1.297844	2.941693
C	0.666678	2.545223	-3.007466
H	-2.300128	-1.086758	-1.884834
H	4.326059	1.284633	2.025399
H	2.767119	3.594820	1.808105
H	4.829455	-2.921116	-1.249402
H	3.685092	0.650703	-1.154681
H	2.608505	-3.953939	2.278756
H	1.477003	-0.398888	2.840671
H	-2.481807	4.279006	1.986821
H	0.576318	2.418432	3.069205
H	-1.050729	4.810952	-2.027113
H	2.047461	2.746687	-1.402273
H	4.050311	-4.612439	0.377544
H	4.480735	0.134062	-3.387734
H	4.917549	-1.520165	-2.929734
H	3.195521	-1.022284	-2.930407
H	6.105778	0.996500	-1.614611
H	5.847605	0.599264	0.102430
H	6.464991	-0.639542	-1.010258
H	-0.294620	-1.864805	3.707080
H	-0.165656	-2.022553	1.939202
H	0.481290	-3.295241	2.992480
H	1.787892	-1.349060	5.098844
H	2.656102	-2.745908	4.424131
H	3.364879	-1.120865	4.297999
H	-2.743870	5.244664	-0.274015
H	-1.085173	0.797401	3.911005
H	-2.317933	1.443661	2.794909
H	-0.886777	0.624946	2.144756

H	-0.708990	3.080391	5.016393
H	-0.440546	4.526343	4.009151
H	-2.028234	3.718965	4.027016
H	1.436584	2.383780	-3.777480
H	0.329208	1.551377	-2.676024
H	-0.179941	3.068393	-3.481175
H	2.665152	4.472153	-3.040644
H	1.118639	5.312568	-2.805991
H	2.291521	5.233022	-1.472630
C	-2.383098	-4.647157	1.418297
C	-1.442319	-4.585147	0.375769
C	-1.243609	-3.418160	-0.354870
C	-2.017428	-2.303615	-0.018577
C	-2.970971	-2.351883	1.027555
C	-3.150404	-3.536131	1.751252
H	-2.508212	-5.578577	1.975529
H	-0.843100	-5.468503	0.141079
H	-0.491812	-3.338811	-1.145349
H	-3.876806	-3.585732	2.566367
C	-2.915700	-0.263027	0.093970
C	-3.248140	1.077815	-0.113578
C	-4.249059	1.636062	0.675483
C	-4.898167	0.883858	1.668647
C	-4.554552	-0.446885	1.888781
C	-3.561769	-1.030613	1.094919
H	-2.708736	1.679370	-0.849598
H	-4.518874	2.684926	0.531356
H	-5.674302	1.352243	2.278329
H	-5.054775	-1.027345	2.668082
N	-1.972816	-1.026726	-0.608533
N	-2.821012	-1.165278	-3.034777
C	-2.287163	-0.062595	-3.836465
H	-2.742858	-0.056816	-4.841610
H	-2.507321	0.890384	-3.335672
H	-1.197002	-0.192248	-3.904541
C	-2.459665	-2.468354	-3.602351
H	-1.362497	-2.523267	-3.656319
H	-2.828957	-3.263377	-2.940763
H	-2.907331	-2.587585	-4.604131
C	-4.263071	-1.053600	-2.817506
H	-4.585210	-1.832902	-2.112766
H	-4.497992	-0.075507	-2.378442
H	-4.812344	-1.172797	-3.766861

IPr-Au-Cl OAc-carbazole

C	-3.275440	-1.213638	-1.822281
C	-2.850871	0.064534	-2.165799
C	-1.493314	0.392855	-2.168930
C	-0.587028	-0.621111	-1.806365
C	-0.987298	-1.911014	-1.420135
C	-2.355082	-2.183565	-1.450784
N	0.817049	-0.323391	-1.886350
C	1.652319	-0.093472	-0.847266
N	2.850364	0.182244	-1.422281
C	2.772031	0.120575	-2.799967
C	1.486227	-0.203458	-3.089816
Au	1.408540	-0.232331	1.118483
Cl	1.394458	-0.475221	3.418763
C	4.036684	0.468947	-0.680412
C	4.886238	-0.596803	-0.343598
C	6.041863	-0.293833	0.380723
C	6.325607	1.014144	0.759027
C	5.453502	2.045991	0.428808
C	4.285769	1.796460	-0.296950
C	4.545661	-2.032376	-0.689747
C	5.636964	-2.689810	-1.532280
C	3.305793	2.914086	-0.591142
C	3.965870	4.079828	-1.324597
C	-1.014852	1.801475	-2.477040
C	-2.016241	2.615122	-3.289248
C	0.010638	-2.961074	-0.968513
C	0.674059	-3.657247	-2.158071
C	4.239281	-2.833164	0.576339
C	2.610885	3.370002	0.693193
C	-0.653286	2.541352	-1.187661
C	-0.601862	-3.970368	-0.001916
H	-3.848344	-0.983760	1.171544
H	3.632320	0.308041	-3.434924
H	0.978079	-0.360764	-4.036479
H	6.721638	-1.099610	0.668358
H	3.627151	-2.022002	-1.295210
H	5.673889	3.065459	0.753826
H	2.526055	2.510929	-1.254123
H	-3.597133	0.825280	-2.398097
H	-0.097058	1.719448	-3.085297
H	-2.732624	-3.158734	-1.149485
H	0.799198	-2.433820	-0.407355
H	7.230018	1.229714	1.333319
H	3.906633	-3.850617	0.317501
H	5.129438	-2.919956	1.219675
H	3.444202	-2.350284	1.165159
H	5.340981	-3.712624	-1.812973
H	5.828095	-2.123479	-2.457018
H	6.587898	-2.760356	-0.980343
H	1.836777	4.119741	0.469389
H	2.122324	2.524105	1.200982
H	3.330774	3.816693	1.397814
H	3.214332	4.843205	-1.578846
H	4.734478	4.568806	-0.704904
H	4.447550	3.748876	-2.257739
H	-4.335768	-1.465931	-1.770560
H	-0.240829	3.538611	-1.413296
H	-1.542905	2.671354	-0.555947
H	0.082027	1.993447	-0.582830
H	-1.571132	3.580698	-3.575802
H	-2.323800	2.091518	-4.207614
H	-2.922127	2.834545	-2.703180
H	0.193437	-4.561851	0.477781
H	-1.194914	-3.471224	0.780136
H	-1.268856	-4.676476	-0.522281
H	1.418995	-4.392732	-1.812226
H	-0.076702	-4.192337	-2.761959
H	1.189255	-2.943077	-2.819300
C	-1.680411	3.324792	2.195469
C	-1.205578	2.042928	2.535332
C	-1.903791	0.893323	2.185701
C	-3.104285	1.040837	1.477242
C	-3.583079	2.333358	1.103993
C	-2.864876	3.475210	1.482057
H	-1.107590	4.206924	2.494133
H	-0.268890	1.932060	3.087371
H	-1.531084	-0.095848	2.458634
H	-3.228242	4.470766	1.210388
C	-4.991120	0.687240	0.363953
C	-6.087718	0.097360	-0.281848
C	-6.980217	0.941702	-0.930756
C	-6.805176	2.340144	-0.943730
C	-5.721326	2.923504	-0.296410
C	-4.800502	2.101052	0.365242
H	-6.188556	-0.993044	-0.269677
H	-7.841148	0.508036	-1.447996
H	-7.530234	2.969893	-1.466595
H	-5.587042	4.009242	-0.304691
N	-3.968553	0.079660	1.036419
C	-4.419192	-3.288565	0.859409
O	-3.639900	-2.492669	1.457209
C	-4.313368	-4.753236	1.300765
H	-4.221573	-4.817767	2.394900
H	-5.176903	-5.333811	0.948010
H	-3.395425	-5.191505	0.875282
O	-5.215094	-3.014455	-0.055087

IPr-Au-Cl OAc-carbazole-TS

C	3.801247	3.032392	-1.068491
C	3.523862	2.172674	-2.125204
C	2.218673	1.742970	-2.374647
C	1.206172	2.238863	-1.533227
C	1.452764	3.119673	-0.464414
C	2.780929	3.494366	-0.245725
N	-0.134758	1.801932	-1.771780
C	-0.786994	0.894416	-0.994865
N	-1.987803	0.731224	-1.615814
C	-2.084580	1.517412	-2.749352
C	-0.914389	2.195840	-2.847199
Au	-0.281629	0.092975	0.747686
Cl	-1.198720	0.125742	3.058656
C	-3.026638	-0.096438	-1.089992
C	-3.894157	0.455872	-0.131585
C	-4.882132	-0.378875	0.393217
C	-4.987467	-1.705615	-0.012784
C	-4.106294	-2.227059	-0.952809
C	-3.103204	-1.431522	-1.513931
C	-3.776322	1.904844	0.297359
C	-4.717859	2.783160	-0.530101
C	-2.095485	-2.019239	-2.480308
C	-2.758473	-2.805240	-3.609010
C	1.905685	0.736106	-3.468757
C	2.952303	0.700180	-4.576563
C	0.325187	3.640557	0.409982
C	-0.536969	4.660515	-0.338531
C	-3.986445	2.104673	1.794150
C	-1.070018	-2.863998	-1.725617
C	1.691965	-0.665563	-2.890949
C	0.798165	4.206170	1.742056
H	1.759040	-0.905800	2.697352
H	-2.972175	1.520085	-3.374458
H	-0.565278	2.923386	-3.573837
H	-5.564074	0.011280	1.150864
H	-2.744976	2.221314	0.082487
H	-4.182026	-3.277501	-1.242274
H	-1.550887	-1.181143	-2.942560
H	4.340172	1.803583	-2.746685
H	0.956139	1.046474	-3.935338
H	3.020207	4.142823	0.596656
H	-0.306427	2.769163	0.654477
H	-5.756747	-2.347077	0.424715
H	-3.787692	3.155687	2.058335
H	-5.022949	1.884069	2.099303
H	-3.292950	1.470089	2.369882
H	-4.594050	3.844367	-0.260676
H	-4.525684	2.684266	-1.609535
H	-5.770045	2.507015	-0.350672
H	-0.294399	-3.245070	-2.407107
H	-0.570852	-2.273713	-0.944773
H	-1.539700	-3.725076	-1.225461
H	-1.999118	-3.144715	-4.330688
H	-3.271133	-3.704343	-3.231992
H	-3.500027	-2.196437	-4.150284
H	4.833367	3.333721	-0.871951
H	1.321559	-1.348411	-3.672413
H	2.632489	-1.071991	-2.492809
H	0.971307	-0.665332	-2.062654
H	2.608513	0.047670	-5.393935
H	3.149790	1.699658	-4.995445
H	3.905162	0.283790	-4.213792
H	-0.075683	4.429269	2.373207
H	1.422381	3.470214	2.270447
H	1.361260	5.146842	1.614164
H	-1.372658	4.989829	0.299616
H	0.055401	5.551146	-0.607324
H	-0.970184	4.250129	-1.262246
C	0.020740	-5.459799	0.874315
C	-0.711565	-4.591525	1.708903
C	-0.319538	-3.275066	1.914026
C	0.837311	-2.817166	1.262763
C	1.588178	-3.691941	0.419673
C	1.170261	-5.014974	0.230196
H	-0.318562	-6.490176	0.735127
H	-1.613727	-4.961773	2.204943
H	-0.888221	-2.585594	2.543503
H	1.738284	-5.688209	-0.419373
C	2.497625	-1.599580	0.495769
C	3.417177	-0.569798	0.240072
C	4.502363	-0.847155	-0.581961
C	4.685503	-2.118176	-1.162907
C	3.777770	-3.143640	-0.914143
C	2.682656	-2.895521	-0.077849
H	3.264031	0.411249	0.695827
H	5.228741	-0.055612	-0.787481
H	5.549138	-2.298704	-1.809254
H	3.922254	-4.132680	-1.359784
N	1.379095	-1.548559	1.299347
C	2.055868	0.774247	3.603151
O	1.973336	-0.527430	3.635865
C	1.830568	1.406958	4.951203
H	2.229903	0.778525	5.758629
H	2.259610	2.416261	4.985005
H	0.737195	1.466885	5.074985
O	2.255267	1.437648	2.599534

IPr-Ag-Cl

C	3.024449	-1.260564	-0.533973
C	2.422291	-0.022720	-0.809653
C	3.073910	1.206671	-0.619152
C	4.388892	1.168844	-0.148935
C	5.017463	-0.042289	0.121174
C	4.341249	-1.242696	-0.066525
N	1.067514	-0.010785	-1.270838
C	-0.000220	-0.000043	-0.440612
N	-1.068077	0.010043	-1.270667
C	-0.679978	0.006535	-2.596640
C	0.679196	-0.008417	-2.596748
C	-2.422769	0.022128	-0.809242
C	-3.074289	-1.207219	-0.618104
C	-4.389188	-1.169265	-0.147681
C	-5.017771	0.041953	0.122051
C	-4.341648	1.242311	-0.066252
C	-3.024935	1.260051	-0.533967
C	-2.370118	-2.530982	-0.840878
C	-2.014026	-3.174653	0.500692
C	-2.279638	2.571240	-0.682440
C	-2.028355	3.206154	0.685979
Ag	0.000433	0.000682	1.616826
Cl	0.001438	0.001665	3.922366
C	2.369823	2.530392	-0.842481
C	2.014217	3.174992	0.498761
C	2.279023	-2.571762	-0.681668
C	2.996877	-3.530935	-1.629524
C	3.178524	3.476275	-1.727329
C	2.028185	-3.206070	0.687120
C	-2.997900	3.530004	-1.630410
C	-3.178924	-3.477524	-1.724910
H	-1.400253	0.013792	-3.409476
H	1.399339	-0.016237	-3.409694
H	-4.840837	2.185763	0.166344
H	-6.044677	0.049577	0.494706
H	-4.925988	-2.105129	0.022563
H	4.840438	-2.186072	0.166374
H	6.044431	-0.049818	0.493659
H	4.925758	2.104754	0.020840
H	-1.295968	2.350206	-1.124062
H	-1.424805	-2.323488	-1.365805
H	1.295191	-2.350864	-1.122993
H	1.424339	2.322681	-1.367018

H	-1.423341	4.120248	0.582815
H	-2.974123	3.481557	1.178430
H	-1.493647	2.515225	1.355937
H	-2.411216	4.452292	-1.761464
H	-3.150299	3.077475	-2.622143
H	-3.985877	3.821115	-1.240759
H	-1.448608	-4.106919	0.346380
H	-1.401866	-2.500665	1.119527
H	-2.921314	-3.418593	1.075242
H	-2.606468	-4.396185	-1.925166
H	-4.121725	-3.780027	-1.243239
H	-3.429813	-3.013232	-2.690765
H	2.410079	-4.453224	-1.760060
H	3.148977	-3.078767	-2.621468
H	3.984965	-3.821981	-1.240104
H	1.422964	-4.120098	0.584588
H	2.974095	-3.481442	1.179317
H	1.493875	-2.514732	1.356972
H	1.448844	4.107210	0.343987
H	1.402194	2.501511	1.118287
H	2.921711	3.419250	1.072852
H	2.606117	4.394860	-1.928069
H	4.121455	3.778988	-1.246044
H	3.429165	3.011319	-2.692927

IPr-Ag-Cl CO_3-carbazole

C	-3.239155	1.117503	1.752794
C	-2.624731	-0.082384	2.085108
C	-1.230907	-0.182815	2.141691
C	-0.484776	0.965745	1.838738
C	-1.077768	2.202469	1.513753
C	-2.471491	2.245880	1.478390
N	0.942153	0.848444	1.775885
C	1.581504	0.351840	0.687385
N	2.894231	0.451139	1.015003
C	3.074768	0.963433	2.288975
C	1.829777	1.207634	2.772642
Ag	0.839444	-0.613539	-1.052733
Cl	1.320188	-2.781706	-2.111146
C	3.954606	0.058152	0.145523
C	4.297135	0.903913	-0.928245
C	5.390348	0.520278	-1.709674
C	6.088321	-0.657044	-1.450848
C	5.679383	-1.504067	-0.428958
C	4.587852	-1.170318	0.379411
C	3.475538	2.136228	-1.243738
C	3.792400	3.300218	-0.304313
C	4.063724	-2.151410	1.408188
C	5.074580	-2.396725	2.527333
C	-0.575377	-1.495291	2.528523
C	-0.958468	-1.884017	3.957949
C	-0.241717	3.439350	1.234510
C	0.131156	4.143323	2.544683
C	3.562860	2.573022	-2.699125
C	3.617203	-3.451246	0.737275
C	-0.890288	-2.613295	1.539396
C	-0.906604	4.439176	0.293973
O	-1.674998	2.687965	-2.162145
C	-0.676460	1.960404	-2.011497
O	-0.872193	0.596829	-2.092012
H	-1.850321	0.322841	-2.003350
H	4.059847	1.108026	2.722963
H	1.498629	1.592223	3.731675
H	5.684745	1.146826	-2.553548
H	2.407495	1.879962	-1.110222
H	6.189700	-2.458503	-0.274896
H	3.167743	-1.703160	1.861914
H	-3.239045	-0.969635	2.256278
H	0.515155	-1.344769	2.513764
H	-2.971426	3.165418	1.173102
H	0.674315	3.096710	0.727608
H	6.937264	-0.934918	-2.082149
H	2.754845	3.294494	-2.885729
H	4.533171	3.036734	-2.956497
H	3.387324	1.721348	-3.373128
H	3.121877	4.144304	-0.528080
H	3.640301	3.032046	0.751881
H	4.836473	3.641957	-0.423028
H	3.122760	-4.106487	1.473297
H	2.908473	-3.257395	-0.088168
H	4.478970	-4.003044	0.324710
H	4.661084	-3.088688	3.279060
H	6.003118	-2.845634	2.136570
H	5.346846	-1.459620	3.039160
H	-4.326557	1.167354	1.663593
H	-0.407166	-3.552985	1.854662
H	-1.971676	-2.796462	1.450703
H	-0.517073	-2.374140	0.532479
H	-0.434913	-2.805587	4.262359
H	-0.702234	-1.087488	4.675430
H	-2.040914	-2.072351	4.040536
H	-0.183019	5.232893	0.045351
H	-1.230248	3.960916	-0.648290
H	-1.772213	4.928487	0.775381
H	0.775579	5.015089	2.341800
H	-0.778756	4.506112	3.052121
H	0.665482	3.491155	3.249662
O	0.506575	2.303988	-1.788090
C	-4.228384	-4.181987	-0.222938
C	-3.036406	-4.007547	-0.960988
C	-2.656208	-2.768177	-1.454507
C	-3.490255	-1.655850	-1.211908
C	-4.713267	-1.833205	-0.467489
C	-5.065126	-3.097710	0.023882
H	-4.490349	-5.174575	0.157654
H	-2.380847	-4.866869	-1.133735
H	-1.711372	-2.646614	-1.992373
H	-5.990068	-3.231269	0.596918
C	-4.369565	0.321842	-1.118916
C	-4.616839	1.707024	-1.226905
C	-5.761011	2.221525	-0.634561
C	-6.674179	1.398408	0.066486
C	-6.443323	0.030062	0.178207
C	-5.297020	-0.522616	-0.409492
H	-3.866328	2.327143	-1.728320
H	-5.958039	3.297398	-0.698922
H	-7.564134	1.842685	0.524378
H	-7.146203	-0.606087	0.728550
N	-3.299440	-0.365458	-1.591101

IPr-Ag-Cl CO_3-carbazole-TS

C	-2.061960	4.268749	1.229082
C	-1.854428	3.122785	1.983514
C	-0.729765	2.317722	1.784558
C	0.178553	2.697762	0.777912
C	-0.027659	3.836771	-0.028421
C	-1.157231	4.615603	0.234198
N	1.380327	1.938375	0.603651
C	1.461181	0.628310	0.247408
N	2.796916	0.380214	0.246904
C	3.534863	1.497484	0.589035
C	2.634659	2.482403	0.822658
Ag	0.161436	-1.063652	-0.055571
Cl	0.143387	-3.329767	0.827647
C	3.367332	-0.917938	0.060049
C	3.597250	-1.383295	-1.245935
C	4.188371	-2.643115	-1.379332
C	4.521662	-3.405206	-0.264356
C	4.247715	-2.933470	1.013667
C	3.652917	-1.683571	1.201786
C	3.211899	-0.552532	-2.445598
C	4.358339	0.362984	-2.873232

C	3.298153	-1.202917	2.595225
C	4.546934	-0.769495	3.364301
C	-0.546740	1.094961	2.663804
C	-0.305050	1.495249	4.119347
C	0.913704	4.245395	-1.148848
C	1.801047	5.421444	-0.729925
C	2.694109	-1.379425	-3.614492
C	2.487227	-2.243488	3.365856
C	-1.716196	0.121756	2.523857
C	0.164014	4.587675	-2.435468
O	-1.193343	1.876645	-2.367735
C	-0.220591	1.109192	-2.490432
O	-0.477816	-0.243563	-2.372333
H	-1.415489	-0.387820	-2.033579
H	4.619430	1.485273	0.635777
H	2.769042	3.514845	1.123858
H	4.367229	-3.038686	-2.381348
H	2.365067	0.110528	-2.184066
H	4.463747	-3.559106	1.882954
H	2.655634	-0.317410	2.480706
H	-2.592687	2.825712	2.731219
H	0.346966	0.558034	2.318342
H	-1.344678	5.493879	-0.387137
H	1.526980	3.365888	-1.399687
H	4.969395	-4.394597	-0.394654
H	2.226307	-0.696428	-4.338648
H	3.486894	-1.953380	-4.128924
H	1.910021	-2.074028	-3.279229
H	4.014617	1.028536	-3.680361
H	4.695688	1.001836	-2.041663
H	5.230531	-0.212712	-3.233834
H	2.099546	-1.804353	4.300148
H	1.640117	-2.605541	2.756537
H	3.107028	-3.113245	3.643060
H	4.278032	-0.394919	4.365876
H	5.240363	-1.617216	3.495354
H	5.091316	0.029666	2.837000
H	-2.954838	4.877279	1.395548
H	-1.514174	-0.803368	3.084870
H	-2.661378	0.555354	2.887616
H	-1.876454	-0.160065	1.472130
H	-0.123892	0.599487	4.735819
H	0.568876	2.160334	4.213877
H	-1.175591	2.023311	4.543027
H	0.889330	4.726173	-3.253606
H	-0.501096	3.742415	-2.687401
H	-0.414747	5.524152	-2.339081
H	2.516656	5.666272	-1.532232
H	1.188513	6.320372	-0.543121
H	2.379585	5.230912	0.187397
O	0.975565	1.410094	-2.705421
C	-4.749460	-4.546278	-0.552031
C	-3.482651	-4.555635	-1.175088
C	-2.759447	-3.389364	-1.373857
C	-3.309184	-2.162945	-0.950709
C	-4.596729	-2.154090	-0.308086
C	-5.303283	-3.347969	-0.114466
H	-5.289219	-5.487535	-0.404853
H	-3.050109	-5.510289	-1.491946
H	-1.765142	-3.409527	-1.824475
H	-6.283508	-3.336635	0.375912
C	-3.698287	-0.070940	-0.519293
C	-3.636247	1.334065	-0.419757
C	-4.687138	2.000867	0.194823
C	-5.806926	1.315230	0.715825
C	-5.889924	-0.069396	0.608389
C	-4.847751	-0.771792	-0.010946
H	-2.775696	1.851779	-0.854710
H	-4.646470	3.092331	0.273728
H	-6.613857	1.877977	1.197374
H	-6.761063	-0.605653	1.002224
N	-2.775361	-0.914133	-1.066294

IPr-Ag-carbazole

C	-2.446326	-2.276605	1.350991
C	-2.878650	-1.416299	0.329267
C	-3.472964	-1.873630	-0.859275
C	-3.650189	-3.251878	-0.998911
C	-3.242743	-4.129833	0.001160
C	-2.644987	-3.647619	1.159389
N	-2.662938	-0.008935	0.482811
C	-1.484637	0.588876	0.200245
N	-1.681344	1.890900	0.506375
C	-2.961708	2.110202	0.975556
C	-3.585999	0.901725	0.962381
C	-0.649395	2.876625	0.379815
C	-0.526410	3.562498	-0.839711
C	0.511903	4.490607	-0.953002
C	1.390172	4.713988	0.102186
C	1.253558	4.006483	1.291110
C	0.232277	3.066697	1.456824
C	-1.424579	3.265982	-2.023704
C	-0.642785	2.505238	-3.096309
C	0.135260	2.264352	2.738663
C	-0.091661	3.165874	3.951353
Ag	0.312398	-0.239366	-0.317825
N	2.207014	-0.953161	-0.598459
C	-3.820474	-0.921623	-1.986587
C	-5.154891	-1.242919	-2.652904
C	-1.771726	-1.774128	2.611872
C	-2.599509	-2.104851	3.853502
C	-2.677519	-0.887344	-3.004465
C	-0.342520	-2.304686	2.724230
C	1.363982	1.371038	2.915057
C	-2.078046	4.526434	-2.586121
H	-4.592359	0.608894	1.247320
H	-3.307665	3.095638	1.273472
H	-2.317440	-4.348591	1.930424
H	-3.385160	-5.205061	-0.129590
H	-4.102401	-3.646985	-1.910544
H	0.645026	5.036040	-1.889859
H	2.201541	5.436838	-0.009461
H	1.965920	4.171970	2.102231
H	-1.706109	-0.677715	2.544447
H	-3.906162	0.088795	-1.556583
H	-2.232881	2.604472	-1.675968
H	-0.740615	1.602534	2.656221
H	0.155137	-1.882617	3.610601
H	-0.322403	-3.401251	2.819347
H	0.258310	-2.037660	1.841030
H	-2.126004	-1.685178	4.754440
H	-3.618456	-1.695101	3.779568
H	-2.686552	-3.192730	4.001649
H	-2.882569	-0.153046	-3.799040
H	-1.724390	-0.617331	-2.525311
H	-2.544982	-1.873510	-3.476510
H	-5.405958	-0.467219	-3.392129
H	-5.124568	-2.202649	-3.191508
H	-5.973278	-1.293653	-1.918875
H	-2.770215	4.267614	-3.402015
H	-2.646268	5.065680	-1.813127
H	-1.331157	5.221928	-2.999255
H	-1.300291	2.223206	-3.933092
H	0.176201	3.120679	-3.500137
H	-0.195606	1.587179	-2.685371
H	1.257420	0.742893	3.812848
H	1.507772	0.707961	2.047958
H	2.283594	1.965682	3.026749
H	-0.213095	2.560273	4.862683
H	0.760902	3.843050	4.116634
H	-0.992764	3.786329	3.829810
C	2.624312	-2.260186	-0.626256

C	4.049525	-2.343520	-0.614683
C	3.337540	-0.174349	-0.560158
C	3.431414	1.224820	-0.504482
C	4.693653	1.803071	-0.457860
C	5.860240	1.015750	-0.469090
C	5.775643	-0.371280	-0.526129
C	4.515821	-0.979293	-0.569888
H	2.528022	1.842588	-0.496783
H	4.781395	2.892345	-0.413530
H	6.838692	1.500796	-0.433414
H	6.683888	-0.980318	-0.533817
C	4.675485	-3.594890	-0.635871
C	3.894875	-4.745351	-0.664073
C	2.490463	-4.656087	-0.671495
C	1.844387	-3.426320	-0.653351
H	5.766600	-3.667304	-0.628214
H	4.372538	-5.727895	-0.680734
H	1.896014	-5.573625	-0.694299
H	0.751772	-3.360801	-0.661255

IPr-Cu-Cl

C	3.002999	-1.284024	-0.348733
C	2.423692	-0.044969	-0.663629
C	3.102516	1.176835	-0.521386
C	4.421358	1.128386	-0.064145
C	5.028118	-0.085824	0.241529
C	4.325030	-1.277153	0.105021
N	1.068601	-0.020608	-1.122455
C	0.000080	-0.000273	-0.289630
N	-1.068521	0.019263	-1.122370
C	-0.679463	0.013019	-2.447817
C	0.679404	-0.015533	-2.447872
C	-2.423574	0.044169	-0.663441
C	-3.102733	-1.177407	-0.520719
C	-4.421588	-1.128429	-0.063587
C	-5.028041	0.086068	0.241581
C	-4.324627	1.277147	0.104659
C	-3.002568	1.283495	-0.349033
C	-2.410730	-2.503981	-0.763898
C	-1.956942	-3.105494	0.568355
C	-2.228273	2.582873	-0.439081
C	-1.978529	3.153950	0.957652
Cu	0.000138	0.000954	1.581180
Cl	0.000195	0.002439	3.691359
C	2.410150	2.503102	-0.765230
C	1.956117	3.105150	0.566686
C	2.228847	-2.583547	-0.438067
C	2.917268	-3.596602	-1.350786
C	3.267355	3.482030	-1.562738
C	1.977372	-3.152683	0.959151
C	-2.915651	3.594595	-1.354066
C	-3.268232	-3.483100	-1.560827
H	-1.399325	0.027642	-3.260881
H	1.399184	-0.030802	-3.260996
H	-4.806137	2.222287	0.365777
H	-6.059136	0.102318	0.602341
H	-4.979886	-2.057511	0.068172
H	4.806782	-2.222052	0.366545
H	6.059189	-0.101668	0.602360
H	4.979400	2.057664	0.067285
H	-1.244874	2.357944	-0.879229
H	-1.505676	-2.305240	-1.359210
H	1.245983	-2.359159	-0.879710
H	1.505127	2.303730	-1.360397
H	-1.351053	4.056788	0.900482
H	-2.923958	3.431001	1.450138
H	-1.468968	2.422699	1.604480
H	-2.306459	4.507247	-1.443676
H	-3.069135	3.185096	-2.364186
H	-3.900149	3.893578	-0.961460
H	-1.400392	-4.041508	0.404341
H	-1.306152	-2.411187	1.122061
H	-2.821729	-3.330047	1.212217
H	-2.692036	-4.391926	-1.792260
H	-4.158994	-3.801010	-0.997063
H	-3.609780	-3.043389	-2.510210
H	2.308064	-4.509273	-1.439995
H	3.072156	-3.188477	-2.361217
H	3.901170	-3.895278	-0.956504
H	1.349820	-4.055502	0.902540
H	2.922204	-3.429142	1.453064
H	1.467218	-2.420476	1.604389
H	1.399087	4.040775	0.402107
H	1.305677	2.410851	1.120858
H	2.820803	3.330515	1.210387
H	2.691041	4.390752	-1.794295
H	4.158309	3.800193	-0.999396
H	3.608625	3.042041	-2.512093

IPr-Cu-Cl CO_3-carbazole

C	3.106371	0.928429	1.864183
C	2.217072	1.989510	1.760551
C	0.837215	1.766230	1.771237
C	0.394254	0.439533	1.891867
C	1.270916	-0.657186	1.996591
C	2.639127	-0.377680	1.979482
N	-1.014004	0.198074	1.810573
C	-1.627153	-0.071866	0.623708
N	-2.947908	-0.122708	0.961651
C	-3.148779	0.127799	2.310863
C	-1.919297	0.336286	2.845695
Cl	-1.128208	1.381389	-2.728451
C	-4.009057	-0.273784	0.021839
C	-4.281601	-1.548487	-0.512356
C	-5.378202	-1.654668	-1.372502
C	-6.152519	-0.544506	-1.701284
C	-5.823216	0.709716	-1.203948
C	-4.732731	0.871860	-0.342825
C	-3.386627	-2.727575	-0.202092
C	-3.584086	-3.250950	1.220886
C	-4.319509	2.258357	0.111049
C	-5.406323	2.926422	0.952222
C	-0.129381	2.926125	1.614135
C	0.074862	3.966170	2.716378
C	0.743135	-2.077817	2.091128
C	0.117595	-2.354063	3.461854
C	-3.488506	-3.862195	-1.210965
C	-3.898489	3.118150	-1.080543
C	-0.045311	3.554687	0.224429
C	1.792825	-3.138017	1.786402
O	1.626408	-3.470292	-1.448856
C	0.628951	-2.757649	-1.284191
O	0.781340	-1.391954	-1.522133
H	1.757988	-1.080555	-1.590177
H	-4.138654	0.127239	2.757918
H	-1.607750	0.568817	3.859733
H	-5.615401	-2.626701	-1.807851
H	-2.337113	-2.386373	-0.294358
H	-6.399429	1.587567	-1.508753
H	-3.430912	2.147109	0.748385
H	2.603676	3.000434	1.609241
H	-1.150329	2.531823	1.729639
H	3.359092	-1.196080	1.997974
H	-0.038136	-2.186905	1.317911
H	-7.003210	-0.656492	-2.379924
H	-2.636731	-4.536880	-1.047938
H	-4.433497	-4.431035	-1.128287
H	-3.389514	-3.482506	-2.238639
H	-2.854172	-4.050791	1.419822
H	-3.426620	-2.468073	1.977225
H	-4.599800	-3.663178	1.361186
H	-3.536309	4.098941	-0.730022

H	-3.083993	2.632062	-1.647230
H	-4.745474	3.297341	-1.764611
H	-5.069532	3.914767	1.305262
H	-6.330262	3.078236	0.369337
H	-5.665601	2.319300	1.834552
H	4.180296	1.108898	1.797612
H	-0.750929	4.399824	0.147103
H	0.968920	3.930259	0.019728
H	-0.296738	2.831444	-0.568295
H	-0.675933	4.769831	2.634901
H	-0.009342	3.514857	3.718381
H	1.070189	4.433518	2.644590
H	1.309383	-4.125770	1.753032
H	2.252249	-2.986741	0.801054
H	2.582243	-3.167896	2.557424
H	-0.285060	-3.379705	3.492381
H	0.875447	-2.262793	4.259207
H	-0.708828	-1.669708	3.698614
O	-0.523083	-3.075592	-0.915993
C	4.342678	3.633584	-1.239873
C	3.069935	3.323220	-1.769982
C	2.619569	2.015515	-1.883061
C	3.467222	0.970598	-1.458562
C	4.773715	1.282438	-0.933655
C	5.194085	2.614478	-0.824350
H	4.658737	4.679095	-1.162956
H	2.414335	4.137041	-2.096728
H	1.622184	1.793972	-2.276406
H	6.182711	2.851067	-0.413907
C	4.328911	-0.950996	-0.943792
C	4.555872	-2.326740	-0.726117
C	5.758775	-2.714744	-0.152964
C	6.752810	-1.775663	0.206457
C	6.546952	-0.417270	-0.013955
C	5.342445	0.009625	-0.589998
H	3.766409	-3.031906	-1.008442
H	5.943494	-3.779370	0.028040
H	7.688319	-2.121737	0.658377
H	7.318032	0.311272	0.262639
N	3.217742	-0.364500	-1.460062
Cu	-0.775520	-0.153509	-1.062965

IPr-Cu-Cl CO_3-carbazole-TS

C	-3.788306	0.337304	2.271066
C	-3.137096	-0.885494	2.377630
C	-1.745923	-0.964459	2.287826
C	-1.041816	0.233145	2.069549
C	-1.670342	1.486228	1.966218
C	-3.063589	1.506189	2.073582
N	0.382910	0.159756	1.966506
C	1.075587	0.137825	0.791182
N	2.372494	0.006819	1.203538
C	2.477902	-0.054519	2.582489
C	1.215593	0.038796	3.065585
Cl	1.818192	-0.910236	-2.628397
C	3.506478	-0.178804	0.354416
C	4.046554	0.919040	-0.338312
C	5.230836	0.702807	-1.046392
C	5.833034	-0.551428	-1.086516
C	5.231892	-1.633840	-0.458034
C	4.043377	-1.470605	0.259438
C	3.330453	2.250967	-0.367684
C	3.505148	3.052253	0.921319
C	3.308272	-2.680915	0.801765
C	4.128350	-3.450681	1.834447
C	-1.019014	-2.294688	2.372911
C	-1.740756	-3.316037	3.247293
C	-0.871132	2.756016	1.741015
C	-0.120097	3.184834	3.004754
C	3.664003	3.095606	-1.588048
C	2.847111	-3.567594	-0.355961
C	-0.754364	-2.869427	0.982025
C	-1.698909	3.923686	1.221748
O	-1.263562	3.858000	-2.073873
C	-0.229876	3.324188	-1.657381
O	-0.040621	1.977151	-2.063309
H	-0.865144	1.610500	-2.431575
H	3.437299	-0.154727	3.081205
H	0.830620	0.040675	4.081023
H	5.672013	1.532463	-1.601178
H	2.250359	2.045466	-0.459544
H	5.667331	-2.632268	-0.553604
H	2.398760	-2.321652	1.303802
H	-3.722667	-1.797880	2.500684
H	-0.040795	-2.103192	2.845308
H	-3.595105	2.449658	1.954912
H	-0.121810	2.546128	0.959066
H	6.758088	-0.693111	-1.653175
H	2.920871	3.904111	-1.641330
H	4.681786	3.525912	-1.545762
H	3.566577	2.497172	-2.506484
H	2.858911	3.941579	0.871874
H	3.208600	2.472732	1.809105
H	4.553592	3.373317	1.057452
H	2.183406	-4.367658	0.010461
H	2.306575	-2.960263	-1.102243
H	3.706639	-4.040855	-0.860462
H	3.557219	-4.311883	2.218286
H	5.063289	-3.840043	1.397648
H	4.400768	-2.813197	2.691190
H	-4.878687	0.378755	2.297031
H	-0.174407	-3.804519	1.052160
H	-1.697964	-3.087518	0.460881
H	-0.195050	-2.167206	0.347758
H	-1.106843	-4.206673	3.385359
H	-1.988628	-2.908983	4.241115
H	-2.675371	-3.658102	2.775395
H	-1.015335	4.719957	0.896313
H	-2.281988	3.644914	0.335157
H	-2.380476	4.322205	1.995004
H	0.468567	4.091980	2.795086
H	-0.827900	3.414596	3.820545
H	0.577930	2.416420	3.365407
O	0.679702	3.758525	-0.934075
C	-2.986905	-4.214843	-1.768577
C	-1.720502	-3.830532	-2.264392
C	-1.326952	-2.501456	-2.324548
C	-2.221538	-1.504267	-1.878154
C	-3.523904	-1.894336	-1.396678
C	-3.887908	-3.246647	-1.336761
H	-3.257239	-5.275202	-1.727635
H	-1.022060	-4.604828	-2.600032
H	-0.332750	-2.210671	-2.677842
H	-4.875129	-3.536523	-0.958289
C	-3.207743	0.359895	-1.386893
C	-3.570563	1.717870	-1.252148
C	-4.844788	2.028448	-0.796655
C	-5.786393	1.029146	-0.466865
C	-5.455031	-0.314449	-0.615216
C	-4.180188	-0.659778	-1.079382
H	-2.865121	2.509137	-1.531972
H	-5.125666	3.082460	-0.695714
H	-6.782036	1.314110	-0.110506
H	-6.187821	-1.095531	-0.380782
N	-2.030232	-0.154679	-1.842306
Cu	0.581084	0.255272	-1.038704

IPr-Cu-carbazole

C	2.107149	-2.356923	1.425452
C	1.265187	-2.688916	0.351217
C	1.748599	-3.158443	-0.881069
C	3.130241	-3.323520	-1.009550

C	3.987592	-3.022780	0.043518
C	3.481109	-2.539852	1.244799
N	-0.143378	-2.474267	0.495359
C	-0.724134	-1.286816	0.207392
N	-2.033500	-1.470262	0.494280
C	-2.272266	-2.750243	0.956506
C	-1.070593	-3.389010	0.956179
C	-2.999794	-0.423845	0.346132
C	-3.702429	-0.331400	-0.867292
C	-4.604438	0.725457	-1.009617
C	-4.785480	1.653001	0.011677
C	-4.064656	1.544242	1.194972
C	-3.152271	0.502801	1.390200
C	-3.424460	-1.275977	-2.019125
C	-2.483743	-0.601734	-3.020785
C	-2.342978	0.428973	2.669330
C	-3.243882	0.309701	3.897971
Cu	0.130162	0.290844	-0.292325
N	1.023960	1.873617	-0.588376
C	0.832368	-3.400972	-2.063293
C	1.019141	-4.789872	-2.669343
C	1.580561	-1.756673	2.713249
C	1.999560	-2.570100	3.936377
C	1.021557	-2.295943	-3.104400
C	2.006293	-0.292016	2.830396
C	-1.388486	1.618872	2.778372
C	-4.695348	-1.784585	-2.693450
H	-0.794502	-4.400394	1.239800
H	-3.264870	-3.087163	1.240786
H	4.166894	-2.280809	2.054340
H	5.065486	-3.150826	-0.079518
H	3.543028	-3.678036	-1.956552
H	-5.162224	0.835273	-1.941821
H	-5.488454	2.478391	-0.122452
H	-4.201083	2.290492	1.980718
H	0.480650	-1.776532	2.669191
H	-0.205699	-3.337430	-1.702197
H	-2.897683	-2.153797	-1.613091
H	-1.726886	-0.482330	2.625977
H	1.580225	0.162577	3.737999
H	3.101604	-0.195874	2.885557
H	1.670370	0.296788	1.962585
H	1.552899	-2.146687	4.849097
H	1.678234	-3.619551	3.852897
H	3.092179	-2.564149	4.073232
H	0.309831	-2.416569	-3.935625
H	0.865937	-1.300732	-2.660311
H	2.039667	-2.316544	-3.523652
H	0.296336	-4.952795	-3.483430
H	2.026118	-4.915870	-3.096559
H	0.872513	-5.580830	-1.918260
H	-4.444279	-2.528541	-3.464759
H	-5.375902	-2.257976	-1.969658
H	-5.246953	-0.974029	-3.194222
H	-2.210705	-1.295887	-3.830679
H	-2.960434	0.281516	-3.474185
H	-1.557636	-0.263274	-2.531124
H	-0.775766	1.538456	3.689311
H	-0.708112	1.672634	1.914327
H	-1.936555	2.572545	2.823883
H	-2.636433	0.193919	4.808775
H	-3.868020	1.207314	4.030690
H	-3.917104	-0.557853	3.822467
C	2.390136	2.032665	-0.604459
C	2.745011	3.413504	-0.641616
C	0.477017	3.135105	-0.611845
C	-0.878569	3.497318	-0.595618
C	-1.201842	4.848355	-0.612546
C	-0.203713	5.839846	-0.647958
C	1.141668	5.488193	-0.665912
C	1.494630	4.134154	-0.646687
H	-1.658976	2.730481	-0.569403
H	-2.253743	5.147031	-0.600326
H	-0.491418	6.893833	-0.662399
H	1.914802	6.261005	-0.692707
C	4.094071	3.785661	-0.652746
C	5.072224	2.797388	-0.625005
C	4.713890	1.436963	-0.588268
C	3.382481	1.040635	-0.579371
H	4.376444	4.841695	-0.680843
H	6.128581	3.076588	-0.632791
H	5.498569	0.675525	-0.568305
H	3.110379	-0.018940	-0.550904

Carbazole

N	1.352071	0.942737	0.000000
C	0.057717	1.414315	0.000000
C	-0.827477	0.304879	0.000000
C	1.347220	-0.434982	0.000000
C	2.416860	-1.332398	0.000000
C	2.120439	-2.691319	0.000000
C	0.793500	-3.151100	0.000000
C	-0.268681	-2.254116	0.000000
C	0.000000	-0.881745	0.000000
H	3.451346	-0.981738	0.000000
H	2.938818	-3.415248	0.000000
H	0.597655	-4.225465	0.000000
H	-1.300024	-2.614725	0.000000
C	-0.414909	2.728450	0.000000
C	-1.792360	2.919970	0.000000
C	-2.682823	1.833761	0.000000
C	-2.208283	0.527023	0.000000
H	0.272496	3.577421	0.000000
H	-2.189482	3.937888	0.000000
H	-3.758516	2.022021	0.000000
H	-2.903182	-0.316123	0.000000
H	2.179176	1.520389	0.000000

IPr-Au-Cl CO_3-amine-TS

C	5.288196	1.317715	-0.983859
C	4.394545	2.058274	-0.218461
C	3.299892	1.452238	0.402067
C	3.105958	0.068421	0.197113
C	4.007802	-0.707674	-0.555501
C	5.099627	-0.048652	-1.133383
N	2.000474	-0.569847	0.846238
C	0.677166	-0.327434	0.607317
N	0.029001	-1.074661	1.542615
C	0.919605	-1.778776	2.331112
C	2.159096	-1.464105	1.889259
Cl	-0.404086	3.208220	-1.608945
C	-1.373818	-1.006216	1.813678
C	-2.213583	-2.050526	1.391680
C	-3.569447	-1.953001	1.721446
C	-4.071676	-0.861725	2.417227
C	-3.219128	0.160695	2.816741
C	-1.852843	0.105053	2.532622
C	-1.695747	-3.244783	0.622771
C	-1.412324	-4.418303	1.562609
C	-0.942514	1.215973	3.017993
C	-0.913021	1.273865	4.545758
C	2.416807	2.257137	1.337976
C	2.883649	2.080302	2.783947
C	3.880322	-2.207926	-0.718309
C	4.874924	-2.934072	0.190806
C	-2.644841	-3.659160	-0.497097
C	-1.308409	2.563270	2.400421
C	2.282758	3.725065	0.957908
C	4.054313	-2.650800	-2.167786
O	1.388791	-1.243649	-2.292446
C	0.657411	-2.145851	-1.822780
O	-0.653456	-2.157345	-2.164306

H	-0.960138	-1.236312	-2.425915
H	0.586243	-2.438753	3.124707
H	3.138328	-1.799985	2.211044
H	-4.246828	-2.739959	1.384314
H	-0.740510	-2.979866	0.134593
H	-3.619050	1.026394	3.351676
H	0.077346	0.978448	2.686854
H	4.548055	3.131614	-0.095120
H	1.402313	1.843070	1.244284
H	5.803011	-0.630557	-1.733486
H	2.840877	-2.507636	-0.476588
H	-5.142364	-0.794561	2.629994
H	-2.110951	-4.330773	-1.184406
H	-3.539855	-4.185617	-0.112367
H	-2.949857	-2.770131	-1.070118
H	-0.979866	-5.252509	0.987549
H	-0.690826	-4.155528	2.352333
H	-2.334705	-4.776004	2.055860
H	-0.616150	3.346797	2.750226
H	-1.238837	2.516547	1.301521
H	-2.331884	2.869632	2.672718
H	-0.198482	2.040343	4.889371
H	-1.902789	1.528145	4.960348
H	-0.609001	0.305902	4.974983
H	6.136585	1.812389	-1.466618
H	1.573046	4.215561	1.642816
H	3.237542	4.274720	1.029213
H	1.867445	3.819871	-0.057821
H	2.208619	2.611357	3.475958
H	2.894512	1.018590	3.075676
H	3.902637	2.481086	2.925356
H	3.837610	-3.728341	-2.241745
H	3.304193	-2.115046	-2.770089
H	5.076151	-2.480043	-2.554138
H	4.726496	-4.024412	0.121077
H	5.919693	-2.716747	-0.097070
H	4.771048	-2.651364	1.251218
O	1.007711	-3.064874	-1.031969
Au	-0.110642	0.944853	-0.699734
C	-3.006374	0.499582	-2.188235
C	-3.753234	1.678643	-2.557230
C	-5.079601	1.804102	-2.212890
C	-5.717194	0.782131	-1.485444
C	-4.917632	-0.308678	-1.139554
N	-3.645146	-0.463576	-1.453896
H	-5.630076	2.709729	-2.497010
H	-3.226753	2.477428	-3.085495
H	-6.766965	0.838915	-1.186296
H	-5.359056	-1.127436	-0.547235
N	-1.733163	0.308607	-2.527635
H	-1.413304	1.086466	-3.102129

IPr-Au-amido

N	3.130278	1.633489	-0.344884
C	2.941797	1.581786	-1.681891
C	3.920563	2.130760	-2.560747
C	5.055209	2.713496	-2.033485
C	5.235962	2.760406	-0.644267
C	4.230562	2.199429	0.135653
N	1.811780	1.001941	-2.128027
Au	0.421166	0.241014	-0.873918
C	-0.911394	-0.487334	0.409244
N	-0.921298	-1.716212	0.978496
C	-1.952406	-1.839093	1.889032
C	-2.610480	-0.650061	1.887906
N	-1.958651	0.157272	0.976320
C	0.062105	-2.719459	0.706045
C	1.245306	-2.711695	1.461103
C	2.196241	-3.693384	1.168616
C	1.972642	-4.631352	0.167114
C	0.793090	-4.606012	-0.570333
C	-0.189428	-3.645464	-0.318923
C	1.511167	-1.686972	2.546011
C	2.722318	-0.816444	2.211667
C	-1.437757	-3.568497	-1.174628
C	-1.099797	-2.975220	-2.543812
C	-2.288038	1.522368	0.701575
C	-3.237920	1.794221	-0.295924
C	-3.528326	3.135533	-0.555880
C	-2.891381	4.153302	0.147529
C	-1.949852	3.850952	1.124542
C	-1.624691	2.525213	1.425689
C	-3.868046	0.686350	-1.116333
C	-3.177415	0.585639	-2.478201
C	-0.590910	2.211850	2.488767
C	0.787997	2.750984	2.108603
C	-5.379515	0.843621	-1.258886
C	-1.042214	2.717651	3.859239
C	1.649184	-2.359361	3.912143
C	-2.147686	-4.914193	-1.299426
H	-3.473958	-0.303524	2.447762
H	-2.118844	-2.753980	2.449763
H	-4.256432	3.388227	-1.329559
H	-3.687586	-0.263095	-0.588276
H	-1.449107	4.659628	1.661773
H	-0.502557	1.116952	2.558795
H	3.132362	-3.715716	1.731142
H	0.638330	-1.018758	2.597999
H	0.637548	-5.339024	-1.364996
H	-2.136932	-2.874466	-0.682756
H	-3.127767	5.196658	-0.074465
H	-3.579490	-0.260362	-3.057604
H	-3.332075	1.505534	-3.064097
H	-2.092210	0.441698	-2.358997
H	-5.804601	-0.023192	-1.787754
H	-5.872680	0.921561	-0.277998
H	-5.645411	1.739922	-1.840574
H	1.520632	2.497137	2.890856
H	1.154984	2.324563	1.161649
H	0.776010	3.848098	2.009582
H	-0.313719	2.429730	4.632925
H	-1.125311	3.815852	3.875510
H	-2.023013	2.304476	4.141303
H	2.731401	-5.387384	-0.048350
H	2.882670	-0.072610	3.008263
H	3.640692	-1.419850	2.133648
H	2.590998	-0.268029	1.265205
H	1.779794	-1.603119	4.701598
H	0.761233	-2.962114	4.158329
H	2.524777	-3.026858	3.944956
H	-2.011894	-2.842428	-3.146787
H	-0.608198	-1.995834	-2.434891
H	-0.415857	-3.635039	-3.100839
H	-3.085463	-4.800522	-1.864628
H	-1.532080	-5.652740	-1.836252
H	-2.392129	-5.335699	-0.312500
H	5.808893	3.136594	-2.704173
H	3.762740	2.085480	-3.641287
H	6.118670	3.212258	-0.189726
H	4.319654	2.206913	1.230600
H	1.717751	0.993386	-3.135402

Amine

N	-0.290524	-1.183889	0.000306
C	1.038705	-1.209796	0.001854
C	1.841378	-0.073454	0.004694
C	1.196811	1.166614	0.003064
C	-0.186700	1.218877	-0.004937
C	-0.903664	0.002532	-0.007794
H	1.497460	-2.206078	0.006515
H	2.929236	-0.155108	0.010236
H	1.775877	2.093837	0.006944

H	-0.716378	2.174268	-0.013920
N	-2.270264	-0.019138	-0.052319
H	-2.696265	-0.915865	0.140602
H	-2.783595	0.801495	0.232425
